# Clonal medicine targeting DNA damage response eradicates leukemia

**DOI:** 10.1038/s41375-024-02138-5

**Published:** 2024-01-16

**Authors:** Monika M. Toma, Adam Karami, Margaret Nieborowska-Skorska, Kumaraswamy Naidu Chirtala, Monika Pepek, Emir Hadzijusufovic, Tomasz Stoklosa, Peter Valent, Tomasz Skorski

**Affiliations:** 1 Fels Cancer Institute for Personalized Medicine and Department of Cancer and Cellular Biology, Temple University Lewis Katz School of Medicine, Philadelphia, PA 19140, USA.; 2 Department of Tumor Biology and Genetics, Medical University of Warsaw, Warsaw, Poland.; 3 Ludwig Boltzmann Institute for Hematology and Oncology and Department of Internal Medicine I, Division of Hematology and Hemostaseology, Medical University of Vienna, Vienna, Austria.; 4 Department for Companion Animals & Horses, Clinic for Internal Medicine and Infectious Diseases, University of Veterinary Medicine Vienna, Vienna, Austria.

## TO THE EDITOR:

Clonal diversity plays a key role in poor therapeutic outcomes in acute myeloid leukemia (AML) and myeloproliferative neoplasm (MPN) patients [1, 2]. Therefore, targeting all clones is required to eradicate these diseases. To achieve this goal, we integrated genetic clonal landscape of individual patient samples with the response to DNA double-strand break repair (DSBR) inhibitors to track individual clones’ sensitivity to these drugs. We decided to target DSBR pathways because AML and MPN accumulate high numbers of DSBs, the most lethal of all DNA lesions resulting from altered metabolism [3, 4]. Thus, survival and proliferation of AML and MPN cells depend on DSBR mechanisms which represent a promising targetable vulnerability (Supplementary Fig. S1) [5].

We reported that AML/MPN - associated somatic mutations, e.g., *FLT3(ITD)* and *JAK2(V617F)* accompanied by *TET2mut* and/or *DNMT3Amut* can be applied to track clonal sensitivity to PARP and Polθ inhibitors [4, 6]. Thus, AML and MPN clones may respond differently to DSBR inhibitors depending on their mutational profile, and the treatment should be tailored to the mutation sets carried by malignant clones.

To integrate clonal composition with response to DSBR inhibitors we developed a single-cell targeted DNA sequencing (sctDNA-seq) myeloid panel which illustrated clone-by-clone response to DSBR inhibitors and unraveled the clonal landscape of AML and MPN at a single-cell resolution before and after the treatment (Supplementary Fig. S2). We used AML and MPN somatic mutations as trackable clonal biomarkers to design a patient-tailored therapeutic regimen (“clonal attack”) utilizing DSBR inhibitors simultaneously targeting all malignant clones.

**Fig. 1:**
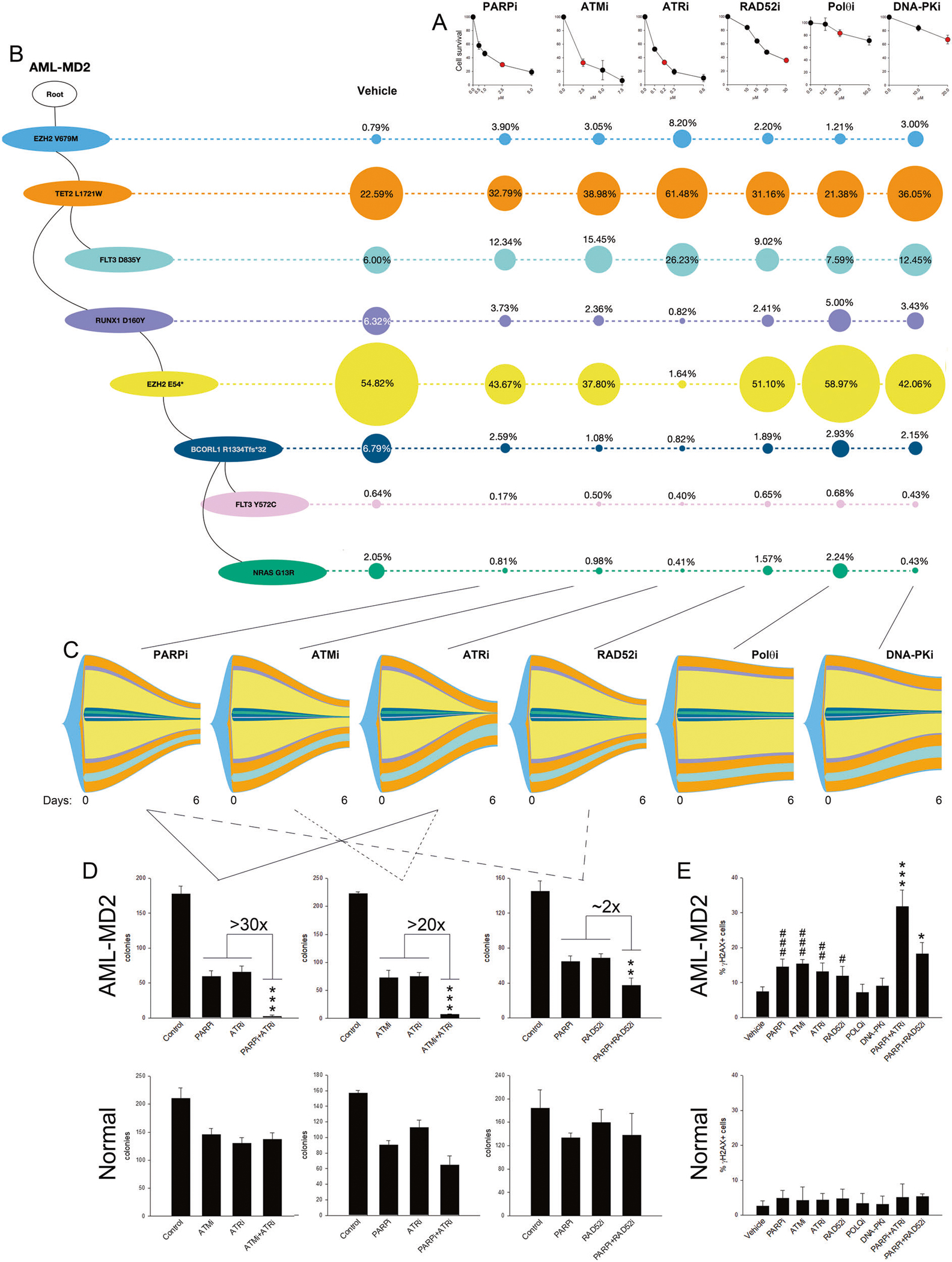
Clonal sensitivity of AML-MD2 cells to DSBR inhibitors. Lin-CD34+ AML patient cells were treated with PARPi olaparib, RAD52i 6-hydroxy-DL-dopa, ATMi KU-60019, ATRi VE-821 and DNA-PKi NU7026 for 6 days following cell survival analysis and sctDNA-seq. **A** Sensitivity to the indicated concentrations of the inhibitors. Results represent mean % ± SD of living cells compared to vehicle-treated control. **B** *Left* - The phylogenetic tree visualizes the predicted clonal structure based on sctDNA-seq data. *Right* - The proportion of clones with a different combination of mutations after the treatment with the red-marked concentrations of the inhibitors indicated in panel **A. C** The fish plots reflect number of cells before (0 days) and 6 days after the treatment and the inferred clonal evolution pattern based on sctDNA-seq data. Chi-Square goodness of fit *p*-value = 0.01578 showing treatment-induced clonal diversity. **D** Sensitivity of Lin-CD34+ AML-MD2 cells and normal counterparts to 2.5 μM olaparib, 30 μM 6-hydroxy-DL-dopa, 0.2 μM VE-821, 25 μM ART558, 2.5 μM KU-60019, and the indicated combinations. Results represent mean number of colonies ± SD; ***p < 0.001 and **p < 0.01 compared to other groups using one-way Anova. **E** Mean % of γH2AX-positive cells in Ki67+ cells ± SD; ***p < 0.001 and *p < 0.05 compared to corresponding individual inhibitors groups using one-way Anova and ^###^*p* < 0.001, ^##^*p* < 0.01 and ^#^*p* < 0.05 compared to vehicle using one-way Anova.

Incubation of Lin-CD34 + cells from AML-MD2 patient with six DNA damage response (DDR) inhibitors were overall sensitive to PARPi, ATMi, ATRi and RAD52i, but resistant to Polθi and DNA-PKi (Fig. 1A). sctDNA-seq followed by phylogenetic tree analysis revealed branched multi-clonal architecture with eight clones carrying specific sets of mutations (Fig. 1B-left). Fish plot analysis demonstrated staggering differences between the sensitivity of various clones to ATRi and to PARPi, ATMi and RAD52i (Fig. 1B-right, C). For example, three clones carrying *EZH2(V679M)*, *EZH2(V679M)* + *TET2(L1721W)* and *EZH2(V679M)* + *TET2(L1721W)* + *FLT3(D835Y)* were resistant to ATRi (~ 8%, 61% and 26% survivors, respectively) but more sensitive to PARPi (~ 4%, 33% and 12% survivors, respectively) and ATMi (~3%, 39% and 15% survivors, respectively). Conversely, three other clones carrying *EZH2(V679M)* + *TET2(L1721W)* + *RUNX1(D160Y)*, *EZH2(V679M)* + *TET2(L1721W)* + *RUNX1(D160Y)* + EZH2(E54*), and *EZH2(V679M)* + *TET2(L1721W)* + *RUNX1(D160Y)* + EZH2(E54*) + BCOR1(R1334Tfs*32) + *NRAS(G13R)* were sensitive to ATRi (~1%, 2% and 0.5% survivors, respectively) while less responsive to PARPi (~4%, 44% and 1% survivors, respectively) and ATMi (~2%, 38% and 1% survivors, respectively).

Based on this observation, we hypothesized that simultaneous treatment with ATRi + PARPi or ATRi + ATMi should result in elimination of numerous, if not all AML-MD2 clones. In agreement with this hypothesis, these drug combinations were 20–30× more effective in eliminating clonogenic growth of AML-MD2 cells when compared to individual inhibitors (Fig. 1D). Remarkably, combinations of these inhibitors (ATRi + PARPi and ATRi + ATMi) were only modestly toxic to normal hematopoietic cells (Fig. 1D) bolstering their therapeutic potential. Moreover, combination of the inhibitors displaying similar clonal targeting specificity such as PARPi + RAD52i was only 2× better than individual inhibitors against clonogenic activity of AML-MD2 cells. The effect might result from the induction of intracellular dual synthetic lethality [7].

As expected, AML cells accumulated approximately 3× more spontaneous DSBs detected by γH2AX immunofluorescence when compared to normal counterparts (Fig. 1E, Vehicle). Only the DSBR inhibitors which reduced survival of AML-MD2 cells (PARPi, ATRi, ATMi, RAD52i) increased the percentage of γH2AX-positive leukemia cells. Moreover, the magnitude of sensitivity of leukemia cells to the combinations of PARPi + ATRi and PARPi + RAD52i was proportional to detection of cells with DSBs (γH2AX+). A low number of γH2AX + cells was detected in normal hematopoietic cell populations treated with these DSBR inhibitors.

PARPi and ATRi - mediated accumulation of γH2AX+ AML-MD2 cells and reduction of cell survival was accompanied by induction of differentiation (increased of CD14+/CD11b+, CD11c+, and HLA-DR+ living cells) (Supplementary Fig. S3) which might contribute to strong anti-leukemia activity of the combination [8].

**Fig. 2:**
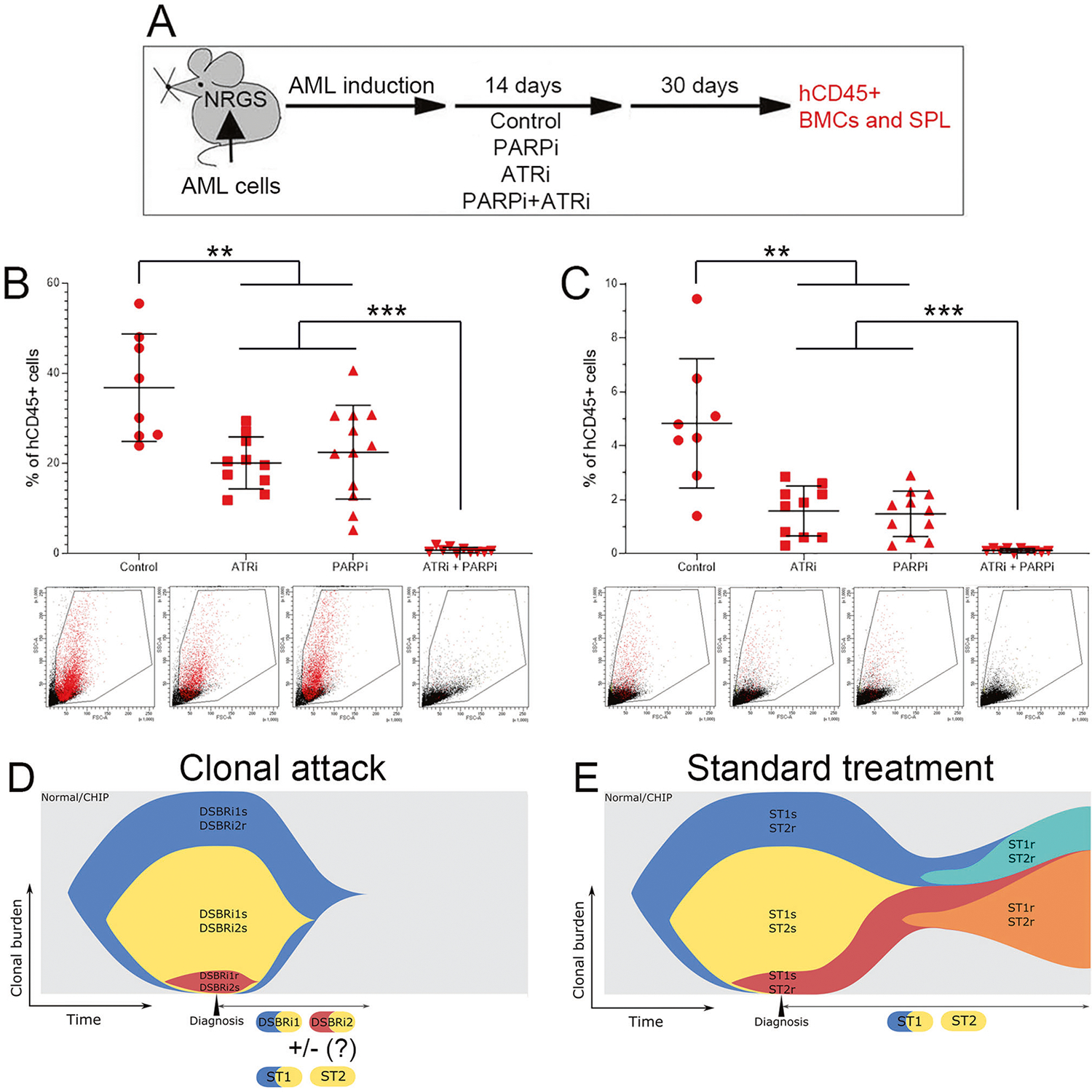
sctDNA-seq/DSBR inhibitors - tailored combination of ATRi + PARPi eradicated AML-MD2 PLX in mice. **A** Experimental protocol. Humanized NRGS immunodeficient mice bearing primary AML-MD2 xenograft were treated for 14 consecutive days with vehicle, PARPi olaparib (30 mg/kg i.p.), ATRi VE-822 (40 mg/kg oral gavage) and the combination of these drugs. The efficiency of these drugs was evaluated by detecting hCD45+ AML-MD2 cells in bone marrow and spleen one month after the end of the treatment to allow regrowth of cells that survived treatment. hCD45+ AML-MD2 patient cells were detected by immunofluorescence in bone marrow (**B**) and spleen (**C**). Results represent mean number of colonies ± SD; ***p < 0.001 and **p < 0.01 using one-way Anova. **D** “Clonal attack” by DDR1 inhibitor + DDR2 inhibitor (e.g., PARPi + ATRi) eventually combined with standard treatment (ST) drugs (ST1, ST2). **E** Standard treatment with ST1 drug + ST2 drug (e.g., quizartinib + azacytidine). Normal/CHIP normal hematopoiesis/clonal hematopoiesis of indeterminate potential.

To determine if the “clonal attack” with DSBR inhibitors eradicates AML in vivo, we treated mice bearing AML-MD2 primary leukemia xenografts with PARPi and/or ATRi (Fig. 2A). Individual drugs reduced the number of hCD45+ AML-MD2 cells in bone marrow and spleen by approximately 2× and 3×, respectively (Fig. 2B, C). Remarkably, the combination of PARPi + ATRi eliminated > 99% of leukemia cells in bone marrow and in spleen of 7/10 and 10/10 mice, respectively. No obvious toxicity in mice was attributed to treatment with PARPi + ATRi in concordance with other studies showing high efficacy and tolerable toxicity of low doses of ATRi + PARP1i [9].

Altogether, these results highlight the remarkable efficiency of a “clonal attack” with the DSBR inhibitors tailored to attack all AML clones in a patient thus reducing the probability of development of time-dependent acquired resistance (Fig. 2D). In comparison, standard treatment (Fig. 2E) often employs chemotherapeutics with or without tyrosine kinase inhibitor (TKi). Chemotherapy dosages are limited by side effects and TKi requires prolonged treatment to effectively eliminate malignant clones. Thus, clones that are inherently resistant to the treatment, not detected by next generation sequencing, and/or those emerging during the prolonged treatment cause the disease relapse. The potential combinatorial effects of DSBR inhibitors and FDA approved drugs (e.g., tyrosine kinase inhibitors, and hypomethylating, genotoxic and pro-apoptotic agents) need to be tested.

To test if clonal targeting by DSBR inhibitors may have broader applicability, additional patient samples were tested. Lin-CD34+ cells from MPN P349 patient were sensitive to PARPi, ATMi, ATRi, RAD52i and DNA-PKi (Supplementary Fig. S4A). sctDNA-seq followed by fish plot analysis revealed similarities and differences between the sensitivity of the clones to these inhibitors (Supplementary Fig. S4B, C). For example, the clone carrying *KMT2A(L2373H)* + *SETBP1(H1100R)* was more sensitive to ATRi (24% survivors) than RAD52i (59% survivors), conversely clones with *KMT2A(L2373H)* and *KMT2A(L2373H)* + *SETBP1(H1100R)* + *FLT3(R834L)* were more sensitive to RAD52i (~40% and 1% survivors, respectively) than ATRi (72% and 4% survivors, respectively). Remarkably, the combination of RAD52i + ATRi was >100× more effective than individual inhibitors against clonogenic growth of P349 cells (Supplementary Fig. S4D). On the other hand, combination of RAD52i + ATMi, the two DSBR inhibitors displaying similar pattern of clonal targeting was only 2x better than individual inhibitors.

Lin-CD34+ cells from MPN P350 patient were sensitive to ATMi, ATRi, and RAD52i, and modestly sensitive to PARPi (Supplementary Fig. S4E). Again, sctDNA-seq followed by fish plot analysis revealed clonal similarities and differences in response to DSBR inhibitors (Supplementary Fig. S4F, G). Clone carrying *TET2(P363L)* + *NRAS(G12D)* was more sensitive to ATRi (~51% survivors) than PARPi (~ 79% survivors), whereas clones with *TET2(P363L)* + *NRAS(G12D)* + *DNMT3A(W330C)* and *TET2(P363L)* + *NRAS(G12D)* + *DNMT3A(W330C)* + *IDH1(R132C)* responded better to PARPi (~0.1% and 0% survivors, respectively) than ATRi (~22% and 3% survivors, respectively). The combination of PARPi + ATRi was >9x more effective in inhibiting clonogenic growth of P349 cells, whereas RAD52i + ATMi displaying similar pattern of clonal targeting were only 2× better than individual inhibitors (Supplementary Fig. S4H).

Clonal targeting by DSBR inhibitors may not be applicable to all AML and MPN samples. For example, Lin-CD34+ cells from AML V18 patient were sensitive to ATMi, ATRi and DNA-PKi (Supplementary Fig. S5A) but no significant differences in major clones’ response were detected (Supplementary Fig. S5B, C). As expected in this context, combinations of ATMi + ATRi and ATMi + DNA-PKi did not exert more potent anti-leukemia effect than individual inhibitors (Supplementary Fig. S5D).

Moreover, Lin-CD34+ cells from AML V20 patient sample were overall resistant to all tested DSBR inhibitors (Supplementary Fig. S5E) and combinations of the inhibitors did not exert significant anti-leukemia effect when compared to individual inhibitors (Supplementary Fig. S5F–H). AML V20 cells accumulated spontaneous DSBs (Supplementary Fig. S5I) therefore resistance to DSBR inhibitors was likely associated with a DSB repair mechanism(s) not targeted by these inhibitors.

The mechanistic aspects of clonal response to DSBR inhibitors are scarcely available. For example, the presence of *DNMT3A(R882S)* in AML V20 was associated with resistance to DSBR inhibitors of almost all clones (Supplementary Fig. S5E–H). DNMT3A mutations (e.g., at the R882) are frequently detected in AML and were associated with resistance to anthracyclines [10]. Remarkably, acquisition of TET2(P562Qfs*6) reversed the resistant phenotype and rendered the clone to be sensitive to multiple DSBR inhibitors. This observation is supported by our report that while *DNMT3Amut* promoted resistance to PARPi in FLT3(ITD)-positive cells, *DNMT3Amut*;*TET2mut;*FLT3(ITD)-positive counterparts were HR-deficient and highly sensitive to PARPi [6]. In conclusion, we postulate that acquisition of a mutation known to modulate DSBR may be a biomarker of the clonal response to DSBR inhibitor(s).

Altogether, “clonal attack” by the combinations of DSBR inhibitors revealed remarkable efficiency in simultaneous eradication of malignant clones from a cohort of AML and MPN patients. This novel approach which, instead of using genotoxic agents to induce DNA damage, takes advantage of metabolic/replication stress-induced DSBs and targets clone-specific vulnerabilities in DSBR pathways. While PARP inhibitors are widely used to treat patients with homologous recombination-deficient tumors including AML [11], ATR, Polθ, ATM, DNA-PKcs kinase inhibitors have been evaluated in cancer clinical trials (NCT04991480) [12–14] and RAD52 inhibitor still awaits clinical development. Moreover, newly developed inhibitors should broaden the spectrum of AML and MPN clones sensitive to DSBR inhibitors [15].

## Supplementary Material

1

## References

[1] Morita K, Wang F, Jahn K, Hu T, Tanaka T, Sasaki Y, Clonal evolution of acute myeloid leukemia revealed by high-throughput single-cell genomics. Nat Commun. 2020;11:5327. 33087716 10.1038/s41467-020-19119-8 PMC7577981

[2] Campbell PJ, Green AR. The myeloproliferative disorders. N Engl J Med. 2006;355:2452–66. 17151367 10.1056/NEJMra063728

[3] Nieborowska-Skorska M, Maifrede S, Dasgupta Y, Sullivan K, Flis S, Le BV, .Ruxolitinib-induced defects in DNA repair cause sensitivity to PARP inhibitors in myeloproliferative neoplasms. Blood. 2017;130:2848–59. 29042365 10.1182/blood-2017-05-784942 PMC5746670

[4] Vekariya U, Toma M, Nieborowska-Skorska M, Le BV, Caron MC, Kukuyan AM, DNA polymerase theta protects leukemia cells from metabolically induced DNA damage. Blood. 2023;141:2372–89. 36580665 10.1182/blood.2022018428 PMC10273171

[5] Trenner A, Sartori AA. Harnessing DNA double-strand break repair for cancer treatment. Front Oncol. 2019;9:1388. 31921645 10.3389/fonc.2019.01388 PMC6921965

[6] Maifrede S, Le BV, Nieborowska-Skorska M, Golovine K, Sullivan-Reed K, Dunuwille WMB, TET2 and DNMT3A mutations exert divergent effects on DNA repair and sensitivity of leukemia cells to PARP inhibitors. Cancer Res. 2021;81:5089–101. 34215619 10.1158/0008-5472.CAN-20-3761 PMC8487956

[7] Sullivan-Reed K, Bolton-Gillespie E, Dasgupta Y, Langer S, Siciliano M, Nieborowska-Skorska M, Simultaneous targeting of PARP1 and RAD52 triggers dual synthetic lethality in BRCA-deficient tumor cells. Cell Rep. 2018;23:3127–36. 29898385 10.1016/j.celrep.2018.05.034 PMC6082171

[8] Stubbins RJ, Karsan A. Differentiation therapy for myeloid malignancies: beyond cytotoxicity. Blood Cancer J. 2021;11:193. 34864823 10.1038/s41408-021-00584-3 PMC8643352

[9] Zimmermann M, Bernier C, Kaiser B, Fournier S, Li L, Desjardins J, Guiding ATR and PARP inhibitor combinationswith chemogenomic screens. Cell Rep. 2022;40:111081. 35830811 10.1016/j.celrep.2022.111081

[10] Guryanova OA, Shank K, Spitzer B, Luciani L, Koche RP, Garrett-Bakelman FE, DNMT3A mutations promote anthracycline resistance in acute myeloid leukemia via impaired nucleosome remodeling. Nat Med. 2016;22:1488–95. 27841873 10.1038/nm.4210 PMC5359771

[11] Le BV, Podszywałow-Bartnicka P, Piwocka K, Skorski T. Pre-existing and acquired resistance to PARP inhibitor-induced synthetic lethality. Cancers. 2022;14:5795. 36497275 10.3390/cancers14235795 PMC9741207

[12] Barnieh FM, Loadman PM, Falconer RA. Progress towards a clinically-successful ATR inhibitor for cancer therapy. Curr Res Pharmacol Drug Discov. 2021;2:100017. 34909652 10.1016/j.crphar.2021.100017 PMC8663972

[13] Lavin MF, Yeo AJ. Clinical potential of ATM inhibitors. Mutat Res. 2020;821:111695. 32304909 10.1016/j.mrfmmm.2020.111695

[14] Hu S, Hui Z, Lirussi F, Garrido C, Ye XY, Xie T. Small molecule DNA-PK inhibitors as potential cancer therapy: a patent review (2010-present). Expert Opin Ther Pat. 2021;31:435–52. 33347360 10.1080/13543776.2021.1866540

[15] Groelly FJ, Fawkes M, Dagg RA, Blackford AN, Tarsounas M. Targeting DNA damage response pathways in cancer. Nat Rev Cancer. 2023;23:78–94. 36471053 10.1038/s41568-022-00535-5

