# Supplementary material for: Clonal medicine targeting DNA damage response eradicates leukemia
Source: Leukemia. Author manuscript; Available in PMC 2024 Mar 13. (PMC10912018; doi:10.1038/s41375-024-02138-5)
Supplement: 1 [file NIHMS1962913-supplement-1.pdf]

Supplemental Figures and Tables.

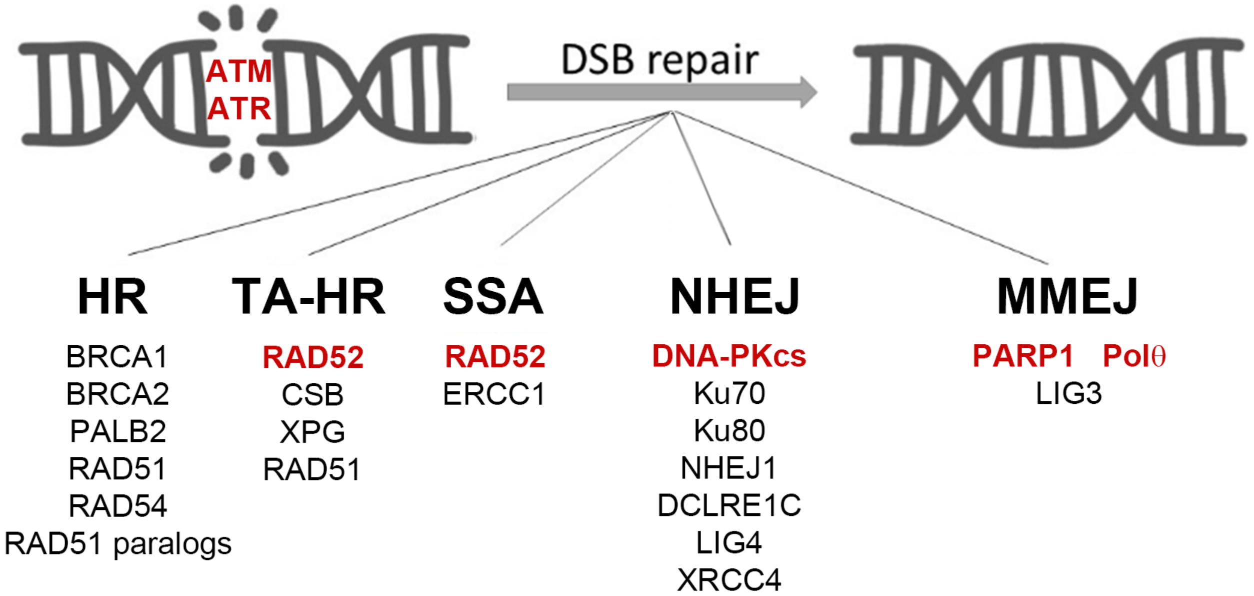

Supplemental Figure S1. DSBR mechanisms. DSBR pathways that sense (ATM and ATR kinases) and repair (RAD51-mediated homologous recombination = HR, RAD52-mediated transcription associated homologous recombination = TA-HR and single strand annealing = SSA, DNA-PK -mediated non-homologous end-joining = NHEJ, PARP1/Polθ-dependent microhomology-mediated end-joining = MMEJ) DSBs (1). Proteins highlighted in red were targeted with small molecule inhibitors: ATMi KU-60019, ATRi VE-821 and VE-822, DNA-PKi NU7026, PARPi olaparib (all from Selleckchem), RAD52i 6-hydroxy-DL-Dopa (Sigma), and Polθi ART558 (MedChemExpress). HR = homologous recombination, TA-HR = transcription associated HR, SSA = single strand annealing, NHEJ = non-homologous end-joining, MMEJ = microhomology-mediated end-joining. RAD51-dependent HR was not targeted because of the anticipated toxicity to normal cells (2).

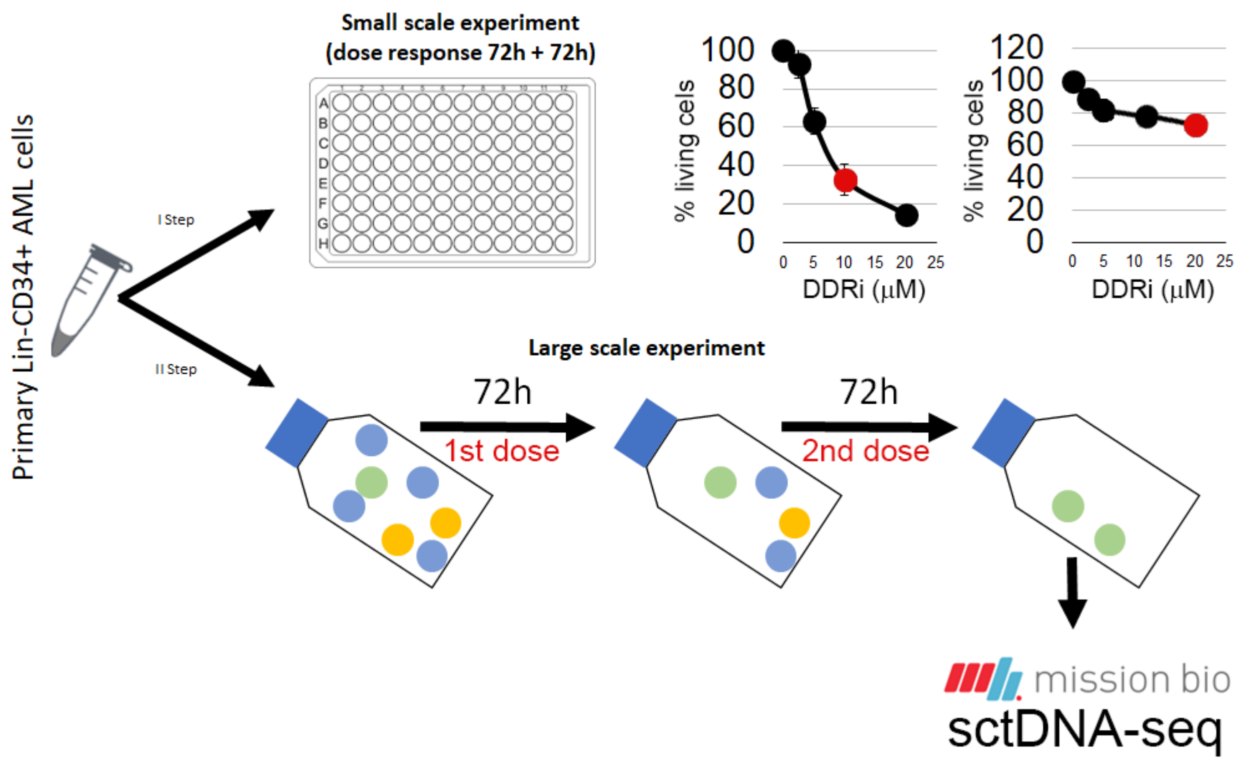

Supplemental Figure S2. Experimental protocol. Clonal fitness did not significantly affect clonal composition after 6 days of tissue culture (3). Initially, DSBR inhibitors were tested in a small-scale experiment to determine the concentrations eliminating 60-80% of malignant cells (if cells were sensitive) or the highest concentrations tested (if cells were resistant). The cells were seeded in 96-well plate at the density of $2.5x10^5$ cells/ml and treated with olaparib, KU-60019, VE-821, NU7026, ART558 and 6-hydroxy-DL-Dopa for 72h after which media was replaced and fresh dose of the inhibitors was added for another 72h. Viability was measured using a trypan blue exclusion test. Large scale experiments were performed to collect the sample for sctDNA-seq. The cells were seeded at the density of $2.5x10^5$/ml and treated with selected doses of the inhibitors for 72h + 72h as in the small-scale experiment. Predetermined drug concentrations in a small-scale experiment were applied to treat cells in a large-scale experiment (marked by red dots in Figures 1A, 2A and 3A). After that the cells were spun down in the Ficoll (Sigma) gradient, washed twice with PBS and frozen in FBS + 10% DMSO. Development of sctDNA-seq custom panel: To design our custom sctDNA-seq panel detecting the most frequently mutated loci among the myeloid malignancies we performed a literature search as well as deep analysis of VarSome (https://doi.org/10.1093/bioinformatics/bty897), COSMIC (https://cancer.sanger.ac.uk) and ClinVar (http://www.ncbi.nlm.nih.gov/clinvar/) databases exploring pathogenicity and frequency of somatic mutations in human cancer cases. The core of our panel was based on myeloid (MYE) panel (Mission Bio Inc). We decided to add the variants within already included genes in addition to adding new genes (*SRSF2, ANKRD26, GNB1, BCORL1, TTN, MUC16, CEBPA, NOTCH1, CUX1*) to the analysis. Our search of clinically relevant variants was focused on single nucleotide variants (SNVs) (excluding synonymous mutations), copy number variants (CNVs) and indels (excluding in frame mutations) mostly within coding regions of the genes. Sorting Intolerant from Tolerant (SITF) and PolyPhen-2 databases were used to predict functional impact and pathogenicity of the variants by analysis of evolutional conservation of the gene regions. Total of 54 genes (Supplemental Table S1) and 1396 of targets within their sequences (Supplemental Table S2 and S3) were analyzed by sctDNA-seq. Normal bone marrow cells from

generated from the custom made Tapestri platform was processed using Mission Bio's Tapestri Pipeline (Tapestri 2.0.2) for adapter trimming (Cutadapt), sequence alignment (reference genome hg19), barcode correction, cell finding, and variant calling (GATK). Annotations for the filtered variants were curated using the Integrative Genomics Viewer (4). Initial clonal architectures were determined using the genotype clustering analysis including zygosity information with the Tapestri Insight software package. Sensitivity of the clones was visualized using the fishplot package in R. We used the SCITE software to infer phylogenetic trees of the driver mutations from sctDNA-seq data. SCITE phylogenetic inference is based on Bayesian approach which allows to quantify uncertainty in the inferred clonal architectures by sampling trees based on the model's posterior distribution. Steps were performed using the methodology described previously (5). SCITE software was run with a chain length of 900000 for each repetition. We used an estimated allele dropout (ADO) rate of 4.5% and a false-positive rate (FPR) of 1.0%. Further data analysis was performed using a customized R script. Graphical visualization of the phylogenies was inferred using the Graphviz (https://graphviz.org/), an open-source graph visualization software.

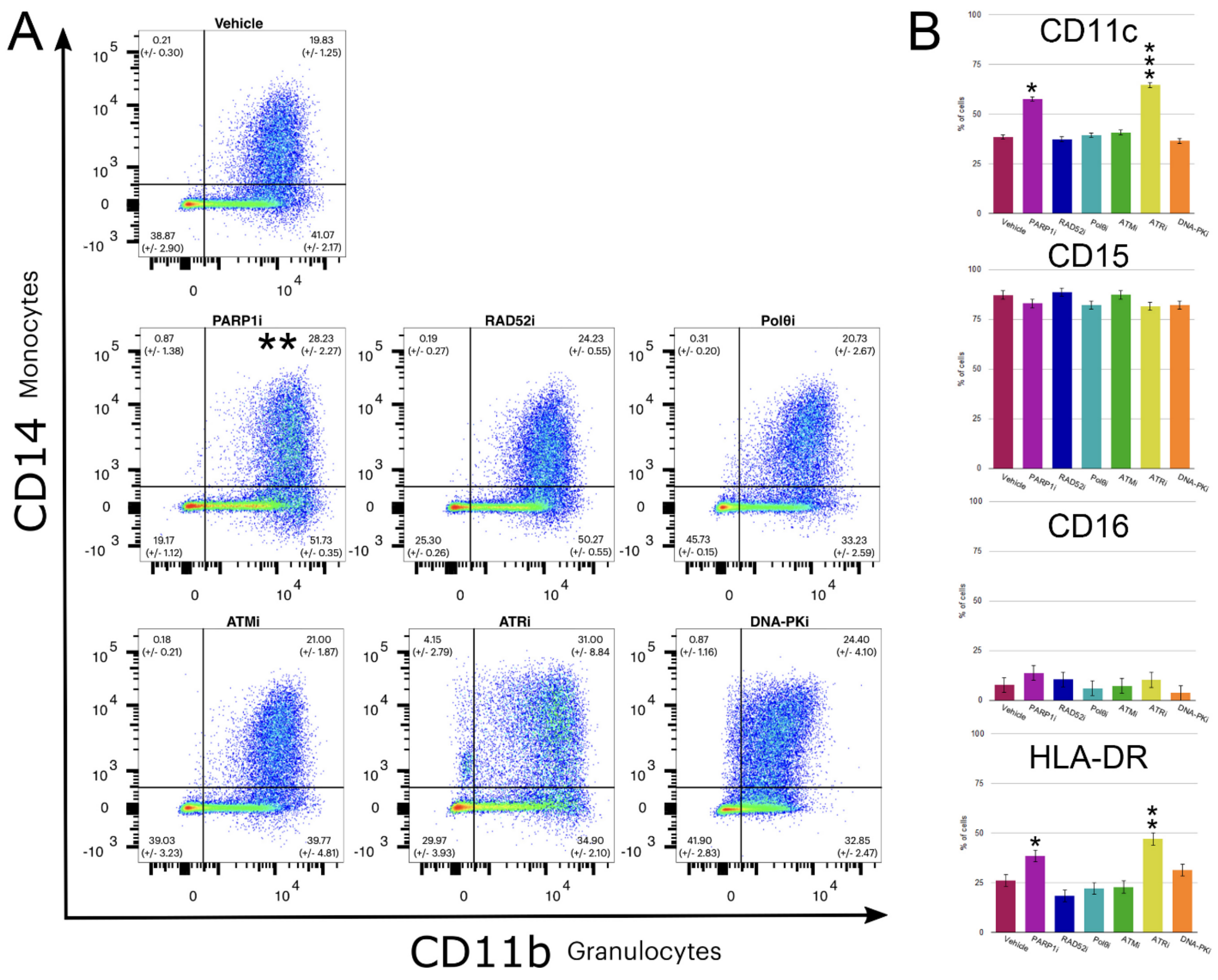

Supplemental Figure S3. Differentiation status of AML-MD2 cells treated with DSBR inhibitors. Since DNA damage can induce hematopoietic cell differentiation (6), we tested myeloid markers in Lin-CD34+ cells treated with the indicated inhibitors as described in Figure 1. (**A**) CD14+ and/or CD11c+ cells, and (**B**) CD11c+, CD15+, CD16+ and HLA-DR+ cells. Results represent mean % of positive cells ± SD from 3-5 experiments. The following antibodies were employed: BUV737-anti-human CD11b (BD Biosciences), APC-anti-human CD11c, BV421-anti-human CD14, BV605-anti-human CD16 and PE-Cy5-anti-human CD15 (BioLegend), and PerCP-eFluor710-anti-human HLA-DR (Thermo Fisher). Immunostained cells were analyzed on FACS Symphony A5; graph preparation using FlowJo v10. Dead cells were excluded from the analysis using PI staining (BD Biosciences).

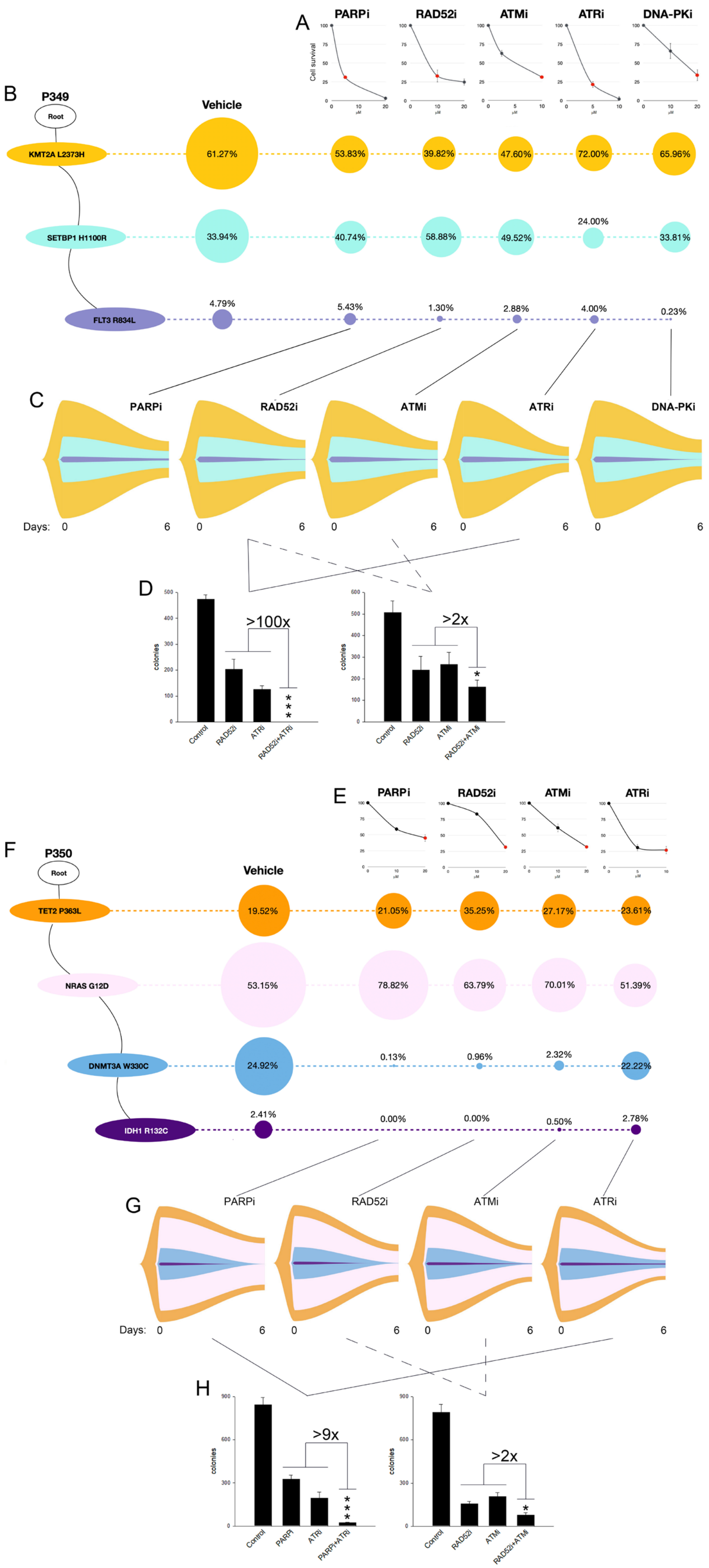
A
PARPi
RAD52i
ATMi
ATRi
DNA-PKi
Cell survival
B
P349
Root
Vehicle
KMT2A L2373H
61.27%
53.83%
39.82%
47.60%
72.00%
65.96%
SETBP1 H1100R
33.94%
40.74%
58.88%
49.52%
24.00%
33.81%
FLT3 R834L
4.79%
5.43%
1.30%
2.88%
4.00%
0.23%
C
PARPi
RAD52i
ATMi
ATRi
DNA-PKi
Days: 0
6
D
colonies
>100x
Control
RAD52i
ATRi
RAD52i+ATRi
>2x
Control
RAD52i
ATMi
RAD52i+ATMi
E
PARPi
RAD52i
ATMi
ATRi
F
P350
Root
Vehicle
TET2 P363L
19.52%
21.05%
35.25%
27.17%
23.61%
NRAS G12D
53.15%
78.82%
63.79%
70.01%
51.39%
DNMT3A W330C
24.92%
0.13%
0.96%
2.32%
22.22%
IDH1 R132C
2.41%
0.00%
0.00%
0.50%
2.78%
G
PARPi
RAD52i
ATMi
ATRi
Days: 0
6
H
colonies
>9x
Control
PARPi
ATRi
PARPi+ATRi
>2x
Control
RAD52i
ATMi
RAD52i+ATMi

Supplemental Figure S4. Clonal sensitivity of MPN P349 and P350 cells to DSBR inhibitors. Lin-CD34+ MPN patient cells were treated with PARPi olaparib, RAD52i 6-hydroxy-DL-dopa, ATMi KU-60019, ATRi VE-821 and/or DNA-PKi NU7026 for 6 days following cell survival analysis and sctDNA-seq. (**A, E**) Sensitivity to the indicated concentrations of the inhibitors. Results represent mean % ± SD of living cells compared to vehicle-treated control. (**B, F**) *Left* - The phylogenetic tree visualizes the predicted clonal structure based on sctDNA-seq data. *Right* - The proportion of clones with a different combination of mutations after the treatment with the red-marked concentrations of the inhibitors indicated in panel A. (**C, G**) The fish plots reflect number of cells before (0 days) and 6 days after the treatment and the inferred clonal evolution pattern based on sctDNA-seq data. Chi-Square goodness of fit p-value = 0.0456 and p = 0.0227 for P349 and P350, respectively, showing treatment-induced clonal diversity. (**D**) Sensitivity to 10 μM 6-hydroxy-DL-dopa, 10 μM KU-60019, 5 μM VE-821 and the indicated combinations. (**H**) Sensitivity to 20 μM olaparib, 20 μM 6-hydroxy-DL-dopa, 20 μM KU-60019, 10 μM VE-821 and the indicated combinations. Results in **D** and **H** represent mean number of colonies ± SD; ***$p \leq 0.001$ and *$p \leq 0.05$ compared to other groups using one-way Anova.

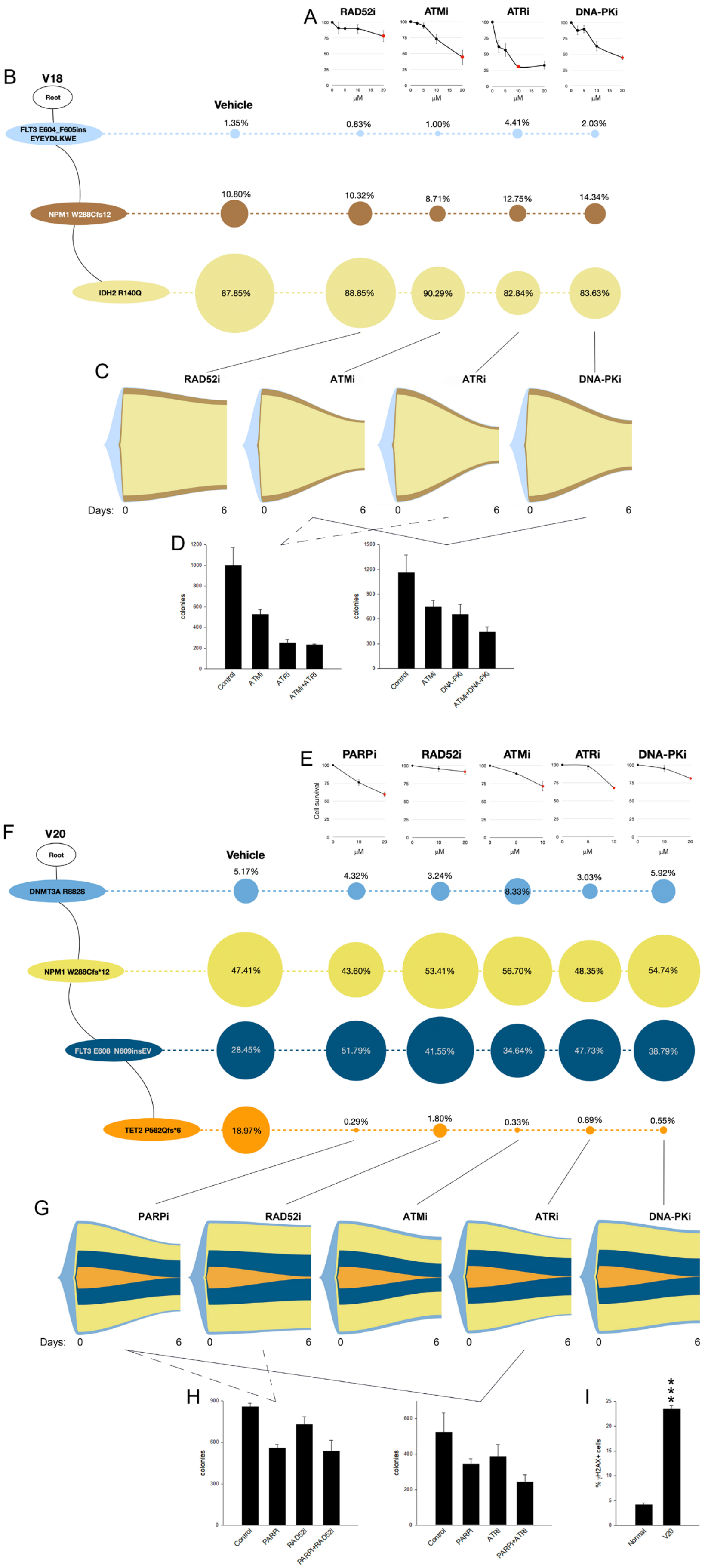

A
RAD52i
ATMi
ATRi
DNA-PKi
μM
B
V18
Root
FLT3 E604_F605ins EYEYDLKWE
NPM1 W288Cfs12
IDH2 R140Q
Vehicle
1.35%
0.83%
1.00%
4.41%
2.03%
10.80%
10.32%
8.71%
12.75%
14.34%
87.85%
88.85%
90.29%
82.84%
83.63%
C
RAD52i
ATMi
ATRi
DNA-PKi
Days: 0
6
D
colonies
Control
ATMi
ATRi
ATMi+ATRi
Control
ATMi
DNA-PKi
ATMi+DNA-PKi
E
PARPi
RAD52i
ATMi
ATRi
DNA-PKi
Cell survival
μM
F
V20
Root
DNMT3A R882S
NPM1 W288Cfs*12
FLT3 E608_N609insEV
TET2 P562Qfs*6
Vehicle
5.17%
4.32%
3.24%
8.33%
3.03%
5.92%
47.41%
43.60%
53.41%
56.70%
48.35%
54.74%
28.45%
51.79%
41.55%
34.64%
47.73%
38.79%
18.97%
0.29%
1.80%
0.33%
0.89%
0.55%
G
PARPi
RAD52i
ATMi
ATRi
DNA-PKi
Days: 0
6
H
colonies
Control
PARPi
RAD52i
PARPi+RAD52i
Control
PARPi
ATRi
PARPi+ATRi
I
% γH2AX+ cells
Normal
V20
***

Supplemental Figure S5. Clonal sensitivity of AML V18 and V20 cells to DSBR inhibitors. Lin-CD34+ AML patient cells were treated with PARPi olaparib, RAD52i 6-hydroxy-DL-dopa, ATMi KU-60019, ATRi VE-821 and DNA-PKi NU7026 for 6 days following cell survival analysis and sctDNA-seq. (**A, E**) Sensitivity to the indicated concentrations of the inhibitors. Results represent mean % ± SD of living cells compared to vehicle-treated control. (**B, F**) *Left* - The phylogenetic tree visualizes the predicted clonal structure based on sctDNA-seq data. *Right* - The proportion of clones with a different combination of mutations after the treatment with the red-marked concentrations of the inhibitors indicated in panel A. (**C, G**) The fish plots reflect number of cells before (0 days) and 6 days after the treatment and the inferred clonal evolution pattern based on sctDNA-seq data. Chi-Square goodness of fit p-value >0.05 for V18 and V20. (**D**) Sensitivity to 20 μM 6-hydroxy-DL-dopa, 20 μM KU-60019, 10 μM VE-821, 20 μM NU7026 and the indicated combinations. (**H**) Sensitivity to 20 μM olaparib, 20 μM 6-hydroxy-DL-dopa, 10 μM VE-821 and the indicated combinations. Results in **D** and **H** represent mean number of colonies ± SD. (**I**) Mean % of γH2AX-positive cells in Ki67+ cells ± SD from healthy donor (Normal) and V20 patient sample; ***p<0.001 using Student t test.

Materials and Methods

Cell culture and treatment

Cells were maintained in StemSpanSFEM media (StemCell Technologies) supplemented with 100 ng/ml SCF, 20 ng/ml IL-3, 100 ng/ml FLT3 ligand, 20 ng/ml G-CSF, 20 ng/ml IL-6 (all purchased from Peprotech). All further procedures were performed on Lin-CD34+ cells obtained using EasySep™ Human Progenitor Cell Enrichment Kit with Platelet Depletion (StemCell Technologies # 19356) and EasySep™ Human CD34 Positive Selection Kit II (StemCell Technologies # 17856) as described before (7). Cells were treated with DSBR inhibitors and analyzed by sctDNA-seq myeloid platform as indicated in Supplemental Figures S1 and S2. Library preparation and sequencing were performed by Single Cell Transcriptomics and Deep Sequencing Core at Johns Hopkins University.

γH2AX

AML-MD2 and healthy donor Lin-CD34+ cells were treated with single drugs and combinations of the inhibitors for 24h and DSBs were measured by detecting γH2AX in Ki67+ population using Alexa Fluor 647-Mouse anti-γH2AX (pS139) antibody (Becton Dickinson) and PE-Anti-Mouse/Rat Ki67 (Invitrogen). Flow cytometric analysis was performed using the FACSymphony A5 (Becton Dickinson).

In vivo treatment

NRGS mice (NOD.Cg-*Rag1*^{tm1Mom} *Il2rg*^{tm1Wjl} Tg(CMV-IL3,CSF2,KITLG)1Eav/J, The Jackson Laboratories) were total body irradiated (600 rads) and injected i.v. with 5 x 10^6 AML-MD2 AML primary cells as described before (8). Treatment started when 3-5% of human hCD45+ leukemia cells were detected in bone marrow. Mice were treated for 14 consecutive days with vehicle (control), olaparib [30mg/kg by intraperitoneal injection, (9)], VE-822 [40mg/kg by oral gavage, (10)], or the combination. Leukemia burden was analyzed 1 month after the end of treatment by flow cytometry detecting hCD45+ cells in bone marrow and spleen as described before (11).

Statistical Analysis

Data are expressed as mean ± standard deviation (SD) from at least 3 independent experiments unless stated otherwise. Comparisons between two groups were done via student's t-test and for multigroup comparisons we used one-way Anova. The statistically significant difference between the clonal proportions was assessed by a Chi-Square test. * p<0.05; ** p<0.01, *** p<0.001 and **** p<0.0001.

Study Approval

Studies involving primary AML and MPN samples were approved by the ethics committee of Temple University Lewis Katz School of Medicine and met all requirements of the Declaration of Helsinki. Animal studies were approved by the Temple University Institutional Animal Care and Use Committee.

Data Availability

The data generated in this study are available upon request from the corresponding author.

References

1. Jackson SP. Sensing and repairing DNA double-strand breaks. Carcinogenesis **2002**;23:687-96.
2. Sonoda E, Sasaki MS, Buerstedde JM, Bezzubova O, Shinohara A, Ogawa H*, et al.* Rad51-deficient vertebrate cells accumulate chromosomal breaks prior to cell death. EMBO J **1998**;17:598-608
3. Salehi S, Kabeer F, Ceglia N, Andronescu M, Williams MJ, Campbell KR*, et al.* Clonal fitness inferred from time-series modelling of single-cell cancer genomes. Nature **2021**;595:585-90
4. Robinson JT, Thorvaldsdottir H, Winckler W, Guttman M, Lander ES, Getz G*, et al.* Integrative genomics viewer. Nature biotechnology **2011**;29:24-6
5. Morita K, Wang F, Jahn K, Hu T, Tanaka T, Sasaki Y*, et al.* Author Correction: Clonal evolution of acute myeloid leukemia revealed by high-throughput single-cell genomics. Nature communications **2021**;12:2823
6. Wang J, Sun Q, Morita Y, Jiang H, Gross A, Lechel A*, et al.* A differentiation checkpoint limits hematopoietic stem cell self-renewal in response to DNA damage. Cell **2012**;148:1001-14
7. Nieborowska-Skorska M, Sullivan K, Dasgupta Y, Podszywalow-Bartnicka P, Hoser G, Maifrede S*, et al.* Gene expression and mutation-guided synthetic lethality eradicates proliferating and quiescent leukemia cells. J Clin Invest **2017**;127:2392-406
8. Maifrede S, Nieborowska-Skorska M, Sullivan-Reed K, Dasgupta Y, Podszywalow-Bartnicka P, Le BV*, et al.* Tyrosine kinase inhibitor-induced defects in DNA repair sensitize FLT3(ITD)-positive leukemia cells to PARP1 inhibitors. Blood **2018**;132:67-77
9. Esposito MT, Zhao L, Fung TK, Rane JK, Wilson A, Martin N*, et al.* Synthetic lethal targeting of oncogenic transcription factors in acute leukemia by PARP inhibitors. Nat Med **2015**;21:1481-90
10. Le TM, Poddar S, Capri JR, Abt ER, Kim W, Wei L*, et al.* ATR inhibition facilitates targeting of leukemia dependence on convergent nucleotide biosynthetic pathways. Nature communications **2017**;8:241
11. Maifrede S, Le BV, Nieborowska-Skorska M, Golovine K, Sullivan-Reed K, Dunuwille WMB*, et al.* TET2 and DNMT3A Mutations Exert Divergent Effects on DNA Repair and Sensitivity of Leukemia Cells to PARP Inhibitors. Cancer research **2021**;81:5089-101

Supplemental Table S1

ANKRD26	MPL
ASXL1	MUC16
ATM	MYC
BCOR	MYD88
BCORL1	NF1
BRAF	NOTCH1
CALR	NPM1
CBL	NRAS
CEBPA	PHF6
CHEK2	PPM1D
CSF3R	PTEN
CUX1	PTPN11
DNMT3A	RAD21
ERG	RUNX1
ETV6	SETBP1
EZH2	SF3B1
FLT3	SMC1A
GATA2	SMC3
GNAS	SRSF2
GNB1	STAG2
IDH1	STAT3
IDH2	TET2
JAK2	TP53
KDM6A	TTN
KIT	U2AF1
KMT2A	WT1
KRAS	ZRSR2

Supplemental Table S2

AmpID	chr	amplicon_s	insert_star	insert_end	amplicon_	Ref_Genor	fwd_seq	rev_seq	amplicon_s	review
AMPL2355	chr1	1737715	1737735	1737956	1737975	hg19	CTGGTCCA	TTCTCGTC	NA	0
AMPL1374	chr1	36932094	36932118	36932342	36932362	hg19	AACCAGAC	GAAAAGA	NA	1
AMPL1374	chr1	36932363	36932384	36932529	36932552	hg19	ATCCTCCT	CCCTTTGT	NA	0
AMPL1212	chr1	36933031	36933051	36933262	36933282	hg19	GGGCTGG	TAACTTCT	NA	0
AMPL2227	chr1	36933313	36933334	36933539	36933559	hg19	CCTCCGAC	CATCCTGA	NA	0
AMPL3592	chr1	43814903	43814923	43815083	43815107	hg19	GGGCCGA	CGAACCAA	NA	1
AMPL1178	chr1	1.15E+08	1.15E+08	1.15E+08	1.15E+08	hg19	TGCATAAC	ATTTGCCA	NA	0
AMPL1129	chr1	1.15E+08	1.15E+08	1.15E+08	1.15E+08	hg19	TCAAACAA	CTGTTTGT	NA	0
AMPL1134	chr1	1.15E+08	1.15E+08	1.15E+08	1.15E+08	hg19	AAAATGAA	AACTGGTG	NA	0
AMPL2355	chr2	25457019	25457044	25457246	25457271	hg19	AAATTCCT	CCACTATA	NA	0
AMPL1212	chr2	25458424	25458445	25458669	25458693	hg19	GAGTCTGC	TCAGCAAA	NA	0
AMPL1212	chr2	25459597	25459617	25459840	25459863	hg19	CGACACTC	CCACTGTG	NA	1
AMPL1374	chr2	25461820	25461845	25462068	25462089	hg19	TTTTCTATA	TGTAGTCC	NA	0
AMPL2355	chr2	25463086	25463107	25463317	25463340	hg19	CCACAATG	CTTTCTCTT	NA	0
AMPL2355	chr2	25463346	25463366	25463588	25463610	hg19	AGCTGGG	TATCCTCC	NA	0
AMPL2356	chr2	25464226	25464247	25464460	25464483	hg19	CCATGCCT	ACCAGGG	NA	0
AMPL1138	chr2	25466552	25466572	25466801	25466821	hg19	GGCTGCTT	GCTGAGA	NA	0
AMPL1374	chr2	25467008	25467028	25467188	25467208	hg19	CAGGCCCA	GGTGCTTT	NA	1
AMPL1212	chr2	25467231	25467251	25467480	25467500	hg19	AGGAGAC	TACCAGTA	NA	1
AMPL2439	chr2	25467931	25467956	25468180	25468200	hg19	GAGAGCA	CATCTGCA	NA	0
AMPL1374	chr2	25468677	25468697	25468892	25468911	hg19	GGAGTCCC	GCAGAAG	NA	0
AMPL2356	chr2	25469005	25469025	25469172	25469194	hg19	CCTCTCCA	TGCTTTCC	NA	0
AMPL1137	chr2	25469465	25469485	25469661	25469681	hg19	AGAAAGC	TCCTGACA	NA	1
AMPL1137	chr2	25469766	25469786	25470011	25470032	hg19	GGAGGAG	CCCAGGTG	NA	0
AMPL1042	chr2	25470404	25470425	25470621	25470641	hg19	CCCTGGGA	ATTTCTGC	NA	1
AMPL1212	chr2	25470886	25470905	25471109	25471130	hg19	CTGGTGAA	TTTCCCCA	NA	0
AMPL1212	chr2	25505239	25505259	25505485	25505508	hg19	GAGCCAAC	GCCTCAGA	NA	1
AMPL1374	chr2	25536656	25536676	25536899	25536922	hg19	CACACCCT	ATAATTCC	NA	1
AMPL2337	chr2	1.79E+08	1.79E+08	1.79E+08	1.79E+08	hg19	GTGAATTT	CCAAGTGA	NA	0
AMPL2356	chr2	1.79E+08	1.79E+08	1.79E+08	1.79E+08	hg19	CAGGGAG	GAAATTGA	NA	0
AMPL2337	chr2	1.79E+08	1.79E+08	1.79E+08	1.79E+08	hg19	ACTGAAGA	GAAGTAGA	NA	0
AMPL2356	chr2	1.79E+08	1.79E+08	1.79E+08	1.79E+08	hg19	ACAGAACC	GTGGAATT	NA	0
AMPL2356	chr2	1.79E+08	1.79E+08	1.79E+08	1.79E+08	hg19	TGTATCCG	GCAATAAC	NA	0
AMPL2337	chr2	1.79E+08	1.79E+08	1.79E+08	1.79E+08	hg19	TTGGTTGG	TGTAGATG	NA	0
AMPL2356	chr2	1.79E+08	1.79E+08	1.79E+08	1.79E+08	hg19	TCCTATGA	TTTCAGGT	NA	0
AMPL2356	chr2	1.8E+08	1.8E+08	1.8E+08	1.8E+08	hg19	TTGGACCT	TGACTGCA	NA	0
AMPL2410	chr2	1.98E+08	1.98E+08	1.98E+08	1.98E+08	hg19	CACCCAAA	GCAGATAT	NA	0
AMPL1374	chr2	1.98E+08	1.98E+08	1.98E+08	1.98E+08	hg19	CAAAATCA	GTGTAGAA	NA	0
AMPL1212	chr2	1.98E+08	1.98E+08	1.98E+08	1.98E+08	hg19	CTGACCTG	GCCAACTA	NA	0
AMPL2410	chr2	1.98E+08	1.98E+08	1.98E+08	1.98E+08	hg19	AATAGCCT	GGTCTTGT	NA	0
AMPL2356	chr2	1.98E+08	1.98E+08	1.98E+08	1.98E+08	hg19	CAACTTAC	CTACCATG	NA	0
AMPL1212	chr2	1.98E+08	1.98E+08	1.98E+08	1.98E+08	hg19	GATCATAG	TGTGAAAC	NA	0
AMPL1134	chr2	2.09E+08	2.09E+08	2.09E+08	2.09E+08	hg19	TTATTGCC	AGTCACCA	NA	0
AMPL1374	chr3	38182045	38182068	38182292	38182314	hg19	GCTCATCG	GGTCTGGA	NA	0
AMPL1178	chr3	38182512	38182532	38182761	38182781	hg19	CCAGGGG	GTCTTCAG	NA	0
AMPL1374	chr3	1.28E+08	1.28E+08	1.28E+08	1.28E+08	hg19	CACCTCCT	GTCAGACG	NA	1
AMPL2356	chr3	1.28E+08	1.28E+08	1.28E+08	1.28E+08	hg19	GGAAATCT	GGCCACTA	NA	0
AMPL1631	chr4	55561577	55561604	55561801	55561825	hg19	TATTGTAG	TCCAGGAT	NA	0
AMPL1137	chr4	55589556	55589575	55589778	55589797	hg19	AGCCTTTC	GGAGCATC	NA	0
AMPL1375	chr4	55592005	55592032	55592226	55592255	hg19	TTGTTTTCT	CCTAAACA	NA	0
AMPL2410	chr4	55593430	55593453	55593679	55593699	hg19	CGTAATCG	GCCTGTTT	NA	0
AMPL2410	chr4	55594013	55594033	55594262	55594282	hg19	TCGGGAAC	ATGGTGCA	NA	0
AMPL1375	chr4	55599194	55599226	55599360	55599383	hg19	AAATGAAT	TGTCAAGC	NA	0
AMPL1213	chr4	1.06E+08	1.06E+08	1.06E+08	1.06E+08	hg19	ATGGAACA	ACACTTGG	NA	0
AMPL2356	chr4	1.06E+08	1.06E+08	1.06E+08	1.06E+08	hg19	TTGCAAAA	TGCATTTT	NA	0
AMPL2356	chr4	1.06E+08	1.06E+08	1.06E+08	1.06E+08	hg19	GCAGTGCT	GAGTTAGA	NA	0
AMPL1213	chr4	1.06E+08	1.06E+08	1.06E+08	1.06E+08	hg19	TGATAATG	GAATCCTT	NA	0
AMPL1829	chr4	1.06E+08	1.06E+08	1.06E+08	1.06E+08	hg19	CTTTTCTG	AGAAGGG	NA	0
AMPL2315	chr4	1.06E+08	1.06E+08	1.06E+08	1.06E+08	hg19	CCTCAGAA	GGCCTTCA	NA	0
AMPL2337	chr4	1.06E+08	1.06E+08	1.06E+08	1.06E+08	hg19	GCATCACT	TTTTGGAG	NA	0
AMPL2356	chr4	1.06E+08	1.06E+08	1.06E+08	1.06E+08	hg19	CAGACCAT	TTTGTGCT	NA	0
AMPL1138	chr4	1.06E+08	1.06E+08	1.06E+08	1.06E+08	hg19	TCACATCT	GGTCTGAC	NA	0
AMPL1403	chr4	1.06E+08	1.06E+08	1.06E+08	1.06E+08	hg19	AAACAATA	GCATGGTT	NA	0
AMPL2337	chr4	1.06E+08	1.06E+08	1.06E+08	1.06E+08	hg19	ACTCAGAC	GGTCTCAA	NA	0
AMPL2337	chr4	1.06E+08	1.06E+08	1.06E+08	1.06E+08	hg19	ATGGAGCA	GGTGTCTT	NA	0
AMPL1301	chr4	1.06E+08	1.06E+08	1.06E+08	1.06E+08	hg19	AGATCAGT	CCCTGTGC	NA	0
AMPL1375	chr4	1.06E+08	1.06E+08	1.06E+08	1.06E+08	hg19	GCTTGGCG	AGGCTTTA	NA	0
AMPL1375	chr4	1.06E+08	1.06E+08	1.06E+08	1.06E+08	hg19	GGGGTGTT	ACTTACTC	NA	0
AMPL2356	chr4	1.06E+08	1.06E+08	1.06E+08	1.06E+08	hg19	TATAGACA	GCGATTAT	NA	0
AMPL1152	chr4	1.06E+08	1.06E+08	1.06E+08	1.06E+08	hg19	TACTAGCA	CCTGATTA	NA	0
AMPL1375	chr4	1.06E+08	1.06E+08	1.06E+08	1.06E+08	hg19	TTTAAAGT	GCTGCCAT	NA	0

AMPL1835	chr4	1.06E+08	1.06E+08	1.06E+08	1.06E+08	hg19	TGGAGGA	GGGGCTG	NA	0
AMPL2337	chr4	1.06E+08	1.06E+08	1.06E+08	1.06E+08	hg19	TTCTCTCTT	GGGCTGG	NA	0
AMPL2337	chr4	1.06E+08	1.06E+08	1.06E+08	1.06E+08	hg19	AGTGGAC	TCATGAGT	NA	0
AMPL2316	chr4	1.06E+08	1.06E+08	1.06E+08	1.06E+08	hg19	TCTAGATT	CTTTTCCT	NA	0
AMPL2356	chr4	1.06E+08	1.06E+08	1.06E+08	1.06E+08	hg19	CCATTGGC	AGAGCCA	NA	0
AMPL2356	chr4	1.06E+08	1.06E+08	1.06E+08	1.06E+08	hg19	AAAAGCCC	TGTAGTTA	NA	0
AMPL1301	chr5	1.71E+08	1.71E+08	1.71E+08	1.71E+08	hg19	AAATTACA	CTGTTACA	NA	0
AMPL2439	chr7	1.02E+08	1.02E+08	1.02E+08	1.02E+08	hg19	GGGGGAG	CTGGGCTT	NA	0
AMPL2356	chr7	1.02E+08	1.02E+08	1.02E+08	1.02E+08	hg19	GGGAAGG	CAACATAC	NA	0
AMPL1822	chr7	1.02E+08	1.02E+08	1.02E+08	1.02E+08	hg19	TGGCAGA	ATCAGAG	NA	0
AMPL2439	chr7	1.02E+08	1.02E+08	1.02E+08	1.02E+08	hg19	CCGTGGTT	TAGCTCGC	NA	0
AMPL2338	chr7	1.02E+08	1.02E+08	1.02E+08	1.02E+08	hg19	AAAATATG	AACTCATC	NA	0
AMPL2439	chr7	1.02E+08	1.02E+08	1.02E+08	1.02E+08	hg19	GCTAGCG	GGGTATG	NA	1
AMPL1818	chr7	1.02E+08	1.02E+08	1.02E+08	1.02E+08	hg19	CATTTGCA	CTGTCCAG	NA	1
AMPL1376	chr7	1.4E+08	1.4E+08	1.4E+08	1.4E+08	hg19	AAAATCAA	GCACCAG	NA	0
AMPL1156	chr7	1.4E+08	1.4E+08	1.4E+08	1.4E+08	hg19	TGATTTTT	TGTTTTCC	NA	0
AMPL1213	chr7	1.4E+08	1.4E+08	1.4E+08	1.4E+08	hg19	CATTAGTT	TTCCACAA	NA	0
AMPL1178	chr7	1.4E+08	1.4E+08	1.4E+08	1.4E+08	hg19	TTGCATAC	CCTTTTAG	NA	0
AMPL1213	chr7	1.4E+08	1.4E+08	1.4E+08	1.4E+08	hg19	ATGGTAG	GCAGATTA	NA	0
AMPL1213	chr7	1.4E+08	1.4E+08	1.4E+08	1.4E+08	hg19	TATTTAAT	CAGGTTTG	NA	0
AMPL1376	chr7	1.4E+08	1.4E+08	1.4E+08	1.4E+08	hg19	CCTTTTAT	ACTATTTA	NA	0
AMPL1213	chr7	1.41E+08	1.41E+08	1.41E+08	1.41E+08	hg19	GTAAATAT	AGTTTGCT	NA	0
AMPL2228	chr7	1.41E+08	1.41E+08	1.41E+08	1.41E+08	hg19	AATTTAAG	CAGTTTCT	NA	0
AMPL1376	chr7	1.41E+08	1.41E+08	1.41E+08	1.41E+08	hg19	AAGAAAC	CACTCCAA	NA	0
AMPL1376	chr7	1.41E+08	1.41E+08	1.41E+08	1.41E+08	hg19	TTAACAAG	ACAAATG	NA	0
AMPL2357	chr7	1.49E+08	1.49E+08	1.49E+08	1.49E+08	hg19	TGGTACAA	CCTGTTGT	NA	0
AMPL2228	chr7	1.49E+08	1.49E+08	1.49E+08	1.49E+08	hg19	GGGCTTTC	AGAGAGC	NA	0
AMPL1127	chr7	1.49E+08	1.49E+08	1.49E+08	1.49E+08	hg19	ATAACTGC	CACTGGG	NA	0
AMPL1131	chr7	1.49E+08	1.49E+08	1.49E+08	1.49E+08	hg19	CCCCATGC	CAGGCTTG	NA	0
AMPL1137	chr7	1.49E+08	1.49E+08	1.49E+08	1.49E+08	hg19	GCACAATC	CTATTGCT	NA	0
AMPL1376	chr7	1.49E+08	1.49E+08	1.49E+08	1.49E+08	hg19	CCCCAGCT	AGGTCAA	NA	0
AMPL1376	chr7	1.49E+08	1.49E+08	1.49E+08	1.49E+08	hg19	GGCCAGG	CGGCAGC	NA	0
AMPL1213	chr7	1.49E+08	1.49E+08	1.49E+08	1.49E+08	hg19	CTTCAACT	TTTTGCAG	NA	0
AMPL1376	chr7	1.49E+08	1.49E+08	1.49E+08	1.49E+08	hg19	TTTATGAC	ATGTGGAT	NA	0
AMPL1213	chr7	1.49E+08	1.49E+08	1.49E+08	1.49E+08	hg19	ACCAAGA	GTTTTGTA	NA	0
AMPL1213	chr7	1.49E+08	1.49E+08	1.49E+08	1.49E+08	hg19	TATTCAAT	ACCAAAAC	NA	0
AMPL1179	chr7	1.49E+08	1.49E+08	1.49E+08	1.49E+08	hg19	CATTAATG	AGAACAC	NA	0
AMPL1213	chr7	1.49E+08	1.49E+08	1.49E+08	1.49E+08	hg19	AAATGAA	GGAAACC	NA	0
AMPL1213	chr7	1.49E+08	1.49E+08	1.49E+08	1.49E+08	hg19	CAAGTACA	TTCATATT	NA	0
AMPL1213	chr7	1.49E+08	1.49E+08	1.49E+08	1.49E+08	hg19	GCCACCCT	ATGCCCTT	NA	0
AMPL2357	chr7	1.49E+08	1.49E+08	1.49E+08	1.49E+08	hg19	GGCCCAG	AAGATGA	NA	0
AMPL1376	chr7	1.49E+08	1.49E+08	1.49E+08	1.49E+08	hg19	AAGAGTA	CAAGTCAT	NA	0
AMPL2357	chr7	1.49E+08	1.49E+08	1.49E+08	1.49E+08	hg19	AGCAGTC	TTCCAATC	NA	0
AMPL1376	chr7	1.49E+08	1.49E+08	1.49E+08	1.49E+08	hg19	GGTATAAT	GAAGAAA	NA	0
AMPL1213	chr8	1.18E+08	1.18E+08	1.18E+08	1.18E+08	hg19	AAAAGAG	TCAGGGA	NA	0
AMPL1376	chr8	1.18E+08	1.18E+08	1.18E+08	1.18E+08	hg19	TTCCAACA	GAAGATA	NA	0
AMPL2228	chr8	1.18E+08	1.18E+08	1.18E+08	1.18E+08	hg19	AAACTACA	CTAACTTA	NA	0
AMPL2228	chr8	1.18E+08	1.18E+08	1.18E+08	1.18E+08	hg19	TCATTCCC	GTGCTATT	NA	0
AMPL1131	chr8	1.29E+08	1.29E+08	1.29E+08	1.29E+08	hg19	GACGATG	AACGTAG	NA	1
AMPL1213	chr8	1.29E+08	1.29E+08	1.29E+08	1.29E+08	hg19	GCTGGGA	TCGATGCA	NA	1
AMPL1213	chr8	1.29E+08	1.29E+08	1.29E+08	1.29E+08	hg19	GTGGTCTT	TTCGCTTA	NA	1
AMPL1213	chr8	1.29E+08	1.29E+08	1.29E+08	1.29E+08	hg19	ATCTGGTA	GATAGTCC	NA	0
AMPL5572	chr8	1.29E+08	1.29E+08	1.29E+08	1.29E+08	hg19	CTGCTGCC	AACTCCGG	NA	0
AMPL1213	chr9	5069814	5069846	5070049	5070080	hg19	GAGTTATT	AGACAGT	NA	0
AMPL1213	chr9	5073530	5073549	5073773	5073799	hg19	TGATGGC	GTAGTTTT	NA	0
AMPL1134	chr9	5078120	5078152	5078362	5078386	hg19	TGTTATTT	ATTTCCTG	NA	0
AMPL1314	chr9	1.39E+08	1.39E+08	1.39E+08	1.39E+08	hg19	CTCAGGCA	TTCCTGAG	NA	1
AMPL2357	chr9	1.39E+08	1.39E+08	1.39E+08	1.39E+08	hg19	CTTTGCTG	GAACCAAT	NA	1
AMPL1172	chr9	1.39E+08	1.39E+08	1.39E+08	1.39E+08	hg19	CTCACACC	ATTGTCCG	NA	1
AMPL1172	chr9	1.39E+08	1.39E+08	1.39E+08	1.39E+08	hg19	CACGCTTG	CGGAGCA	NA	1
AMPL2357	chr10	27389222	27389242	27389433	27389451	hg19	GCGACTCG	TTGCCATC	NA	0
AMPL1241	chr10	89624108	89624126	89624305	89624325	hg19	AGCGAGG	GCAGCCG	NA	0
AMPL2357	chr10	89692736	89692768	89692980	89693002	hg19	TTTTTCTTA	GTCTCTGG	NA	0
AMPL3627	chr10	89717544	89717569	89717771	89717799	hg19	TTTTGACA	CCCAATGA	NA	0
AMPL2411	chr10	89720533	89720562	89720732	89720761	hg19	TGTTGACT	GCAAATG	NA	0
AMPL2411	chr10	89720762	89720787	89720921	89720951	hg19	AGTATAGA	GCTGTACT	NA	0
AMPL1214	chr10	1.12E+08	1.12E+08	1.12E+08	1.12E+08	hg19	TTTTCATC	ACAAAGC	NA	0
AMPL1376	chr10	1.12E+08	1.12E+08	1.12E+08	1.12E+08	hg19	TTTTTCAG	ACTTCCTT	NA	0
AMPL1214	chr10	1.12E+08	1.12E+08	1.12E+08	1.12E+08	hg19	CCTCAAAC	CGAAGTTC	NA	0
AMPL1376	chr10	1.12E+08	1.12E+08	1.12E+08	1.12E+08	hg19	CAGTTTTG	CTGAACTT	NA	0
AMPL2440	chr11	32410597	32410615	32410844	32410865	hg19	AGACCCCT	AGCTCAGT	NA	0
AMPL2411	chr11	32410879	32410898	32411119	32411138	hg19	GCCTTGAA	ATGACCCA	NA	0

AMPL2411	chr11	32411142	32411165	32411343	32411364	hg19	CCCTGGGT	AAAATCTA	NA	0
AMPL2411	chr11	32411384	32411403	32411630	32411649	hg19	TTGAGGCT	CGGGCTCT	NA	0
AMPL2411	chr11	32411660	32411679	32411890	32411909	hg19	TGTATCAC	TGACCTGT	NA	0
AMPL2411	chr11	32411936	32411954	32412168	32412188	hg19	AGCCGTGT	TTGGAGAC	NA	0
AMPL2411	chr11	32412368	32412388	32412610	32412629	hg19	AGAAGCA/	TAGACCTT	NA	0
AMPL2411	chr11	32412817	32412836	32413051	32413070	hg19	CACAGCCG	CGGTCTTG	NA	0
AMPL2411	chr11	32413071	32413096	32413253	32413278	hg19	CTCCTGGT	TGAAAATG	NA	0
AMPL2411	chr11	32413281	32413301	32413527	32413547	hg19	CTGCACAC	CCCACACC	NA	0
AMPL2440	chr11	32413557	32413577	32413725	32413746	hg19	GGTCGGAC	CTAAGGGT	NA	0
AMPL1301	chr11	32414016	32414041	32414263	32414285	hg19	AATGTGGC	GTGTGACT	NA	0
AMPL1214	chr11	32417705	32417727	32417953	32417974	hg19	TCCATCCTC	CCTCTTACT	NA	0
AMPL2357	chr11	1.08E+08	1.08E+08	1.08E+08	1.08E+08	hg19	TTCCATCCT	AGAGCCTC	NA	0
AMPL1136	chr11	1.08E+08	1.08E+08	1.08E+08	1.08E+08	hg19	AAGGCCTT	GATCACAC	NA	0
AMPL1376	chr11	1.18E+08	1.18E+08	1.18E+08	1.18E+08	hg19	GCGGATCA	ACTTCAGG	NA	0
AMPL1376	chr11	1.18E+08	1.18E+08	1.18E+08	1.18E+08	hg19	GCCACAAT	GGCAAGG	NA	0
AMPL1214	chr11	1.18E+08	1.18E+08	1.18E+08	1.18E+08	hg19	AATCGAAC	AGATTCCC	NA	0
AMPL1376	chr11	1.18E+08	1.18E+08	1.18E+08	1.18E+08	hg19	TCAAGTAG	CTTTGGAA	NA	0
AMPL2229	chr11	1.18E+08	1.18E+08	1.18E+08	1.18E+08	hg19	CTGGATCA	ACTACTGC	NA	0
AMPL1376	chr11	1.18E+08	1.18E+08	1.18E+08	1.18E+08	hg19	AATTTGTC	CTCTGATC	NA	0
AMPL1376	chr11	1.18E+08	1.18E+08	1.18E+08	1.18E+08	hg19	GAGCCTTT	GAAAGCA	NA	0
AMPL2411	chr11	1.18E+08	1.18E+08	1.18E+08	1.18E+08	hg19	ATTTTATAC	GCTTACTC	NA	0
AMPL1376	chr11	1.18E+08	1.18E+08	1.18E+08	1.18E+08	hg19	CCCTCTTC	ACTGGAAT	NA	0
AMPL1214	chr11	1.18E+08	1.18E+08	1.18E+08	1.18E+08	hg19	CTGGTCAG	GGGCATTT	NA	0
AMPL1214	chr11	1.18E+08	1.18E+08	1.18E+08	1.18E+08	hg19	TGAAAGA	TTTGGGGC	NA	0
AMPL1214	chr11	1.18E+08	1.18E+08	1.18E+08	1.18E+08	hg19	CTAGAACA	GGCCAACA	NA	0
AMPL1214	chr11	1.18E+08	1.18E+08	1.18E+08	1.18E+08	hg19	AAATGAGC	AGGATATT	NA	0
AMPL1214	chr11	1.18E+08	1.18E+08	1.18E+08	1.18E+08	hg19	AGTTTGAG	CAAGGAGC	NA	0
AMPL2230	chr11	1.18E+08	1.18E+08	1.18E+08	1.18E+08	hg19	TGGTTCAG	TGGAGAAC	NA	0
AMPL1214	chr11	1.18E+08	1.18E+08	1.18E+08	1.18E+08	hg19	TACCCATG	CAATGTTT	NA	0
AMPL1836	chr11	1.18E+08	1.18E+08	1.18E+08	1.18E+08	hg19	TCCCCTCC	AGAGGATC	NA	0
AMPL2411	chr11	1.18E+08	1.18E+08	1.18E+08	1.18E+08	hg19	CCATCTTCT	GGATCTCT	NA	0
AMPL1376	chr11	1.19E+08	1.19E+08	1.19E+08	1.19E+08	hg19	TTTTGAAG	TCTTGGGG	NA	0
AMPL2411	chr11	1.19E+08	1.19E+08	1.19E+08	1.19E+08	hg19	GCAAAATT	TCGCTGTT	NA	0
AMPL1214	chr11	1.19E+08	1.19E+08	1.19E+08	1.19E+08	hg19	AATAACCT	GCCACTCC	NA	0
AMPL1376	chr12	11802933	11802953	11803100	11803123	hg19	CCGGGAG	CACGTAGA	NA	0
AMPL1214	chr12	11905300	11905320	11905513	11905532	hg19	CCACCCCG	TCGGGTCA	NA	0
AMPL1214	chr12	11991980	11992007	11992223	11992246	hg19	TTGTTAAC	ACTCTCAC	NA	0
AMPL1376	chr12	12006217	12006239	12006464	12006486	hg19	CATGAGCT	GGTTCTGA	NA	0
AMPL2230	chr12	12022185	12022214	12022377	12022398	hg19	AAAAATAT	ATTATCCA	NA	0
AMPL1214	chr12	12022445	12022467	12022682	12022700	hg19	CACCTATC	ATCAGCTG	NA	1
AMPL1376	chr12	12022707	12022727	12022878	12022900	hg19	CCCCATCA	CTATTCTC	NA	0
AMPL1214	chr12	12037272	12037292	12037518	12037541	hg19	AACCCAAG	GATATCTG	NA	0
AMPL1130	chr12	12038758	12038786	12038972	12038997	hg19	GCTGAAG	CTCCCAAA	NA	0
AMPL1376	chr12	12043675	12043702	12043919	12043940	hg19	AGCATTTT	TGGGACTC	NA	0
AMPL2412	chr12	25362637	25362670	25362859	25362892	hg19	GGTAATGC	GAAGCCAT	NA	0
AMPL1376	chr12	25378493	25378522	25378720	25378749	hg19	ATAACAGT	GTGTTACT	NA	0
AMPL1301	chr12	25380073	25380106	25380285	25380309	hg19	ATTAAATA	CCTGTCTC	NA	0
AMPL1126	chr12	25398047	25398077	25398285	25398313	hg19	TAATGGTT	TGAATATA	NA	0
AMPL2357	chr12	1.13E+08	1.13E+08	1.13E+08	1.13E+08	hg19	TTCACCCA	GTTCCATG	NA	0
AMPL1376	chr12	1.13E+08	1.13E+08	1.13E+08	1.13E+08	hg19	TCACGCCT	CGGAATAT	NA	0
AMPL1214	chr12	1.13E+08	1.13E+08	1.13E+08	1.13E+08	hg19	ACGTTGTA	TGATGTCA	NA	0
AMPL1377	chr12	1.13E+08	1.13E+08	1.13E+08	1.13E+08	hg19	TTGGTCAG	TGCTGGAC	NA	0
AMPL1134	chr13	28592402	28592433	28592644	28592668	hg19	TATTTTGCT	AGATATGT	NA	0
AMPL2357	chr13	28602132	28602160	28602380	28602401	hg19	TATTTTGAC	AGGCACTC	NA	0
AMPL1809	chr13	28608125	28608147	28608349	28608374	hg19	TTCCCTGC	GACTCATC	NA	0
AMPL1247	chr15	90631705	90631725	90631935	90631960	hg19	CCGGTCTG	GTGGAAA	NA	1
AMPL1377	chr17	7572743	7572764	7572991	7573012	hg19	ACAACAAA	ACAGCCAC	NA	0
AMPL1137	chr17	7573800	7573820	7574037	7574057	hg19	GGGCTGGC	TCTCCCCCT	NA	0
AMPL2440	chr17	7576458	7576480	7576648	7576678	hg19	AAAAAGCA	ACCATAAT	NA	0
AMPL2440	chr17	7576679	7576707	7576855	7576880	hg19	ATAAGTTG	ACTGGATG	NA	0
AMPL2339	chr17	7576901	7576922	7577142	7577169	hg19	AGAGGAGC	CCTATCCTC	NA	0
AMPL1668	chr17	7577337	7577359	7577587	7577606	hg19	AAACTGAC	TGGCTCTG	NA	0
AMPL1214	chr17	7578026	7578053	7578275	7578295	hg19	GCAATAGT	TCTTAGGT	NA	0
AMPL3364	chr17	7578333	7578353	7578554	7578577	hg19	CAACCAGC	CTGTCTCC	NA	1
AMPL2339	chr17	7579259	7579279	7579494	7579514	hg19	GATACGGC	CAGGTCCA	NA	1
AMPL2358	chr17	7579525	7579550	7579699	7579723	hg19	GAACCATT	AGACTTCC	NA	0
AMPL2224	chr17	7579724	7579748	7579929	7579949	hg19	GAAAGAC	CTCTTGCA	NA	0
AMPL1377	chr17	29482905	29482932	29483148	29483173	hg19	TTCCTAAA	CCCCAAAA	NA	0
AMPL1202	chr17	29508599	29508627	29508842	29508868	hg19	GATAGTTT	GGTTTTTA	NA	0
AMPL2230	chr17	29509359	29509384	29509596	29509625	hg19	ATTGCTTG	CACAAGAT	NA	0
AMPL1377	chr17	29527911	29527932	29528148	29528169	hg19	CCACAGTA	GTTGGTTG	NA	0
AMPL1214	chr17	29528331	29528364	29528569	29528596	hg19	TTTTTAAAC	TGAAAACC	NA	0

AMPL1133	chr17	29533143	29533165	29533385	29533412	hg19	TGGTCTTA	TCAAAATT	NA	0
AMPL1214	chr17	29551982	29552005	29552231	29552251	hg19	CAAGTTGG	TTCCCTTC	NA	0
AMPL2230	chr17	29553481	29553499	29553723	29553747	hg19	CCCCGATT	TGCTTTGA	NA	0
AMPL1377	chr17	29554035	29554065	29554284	29554304	hg19	AATAGCTA	TGTTTCCT	NA	0
AMPL2230	chr17	29554351	29554374	29554593	29554620	hg19	CCTCAATT	CCATCTTC	NA	0
AMPL1214	chr17	29556004	29556029	29556248	29556273	hg19	AGGTTTAA	CACTGAAA	NA	0
AMPL1214	chr17	29556640	29556661	29556879	29556909	hg19	TTGCCTCT	CTTCATTA	NA	0
AMPL2230	chr17	29557644	29557667	29557890	29557913	hg19	CCATAACT	GCTTGGTT	NA	0
AMPL1214	chr17	29559829	29559848	29560076	29560098	hg19	GCACTGTA	TCTGTGCC	NA	0
AMPL1133	chr17	29562498	29562525	29562747	29562767	hg19	ATCAGTCA	TACTGGCC	NA	0
AMPL1214	chr17	29562771	29562800	29563008	29563033	hg19	TAATGACA	GGATCCAC	NA	0
AMPL1832	chr17	29575873	29575902	29576118	29576142	hg19	TTTAAAGA	CATACCTG	NA	0
AMPL1215	chr17	29588506	29588528	29588751	29588775	hg19	AAGCTAAG	GTGTTGCC	NA	0
AMPL1377	chr17	29652697	29652726	29652912	29652936	hg19	AAAAATGA	AGGTCCAC	NA	0
AMPL2230	chr17	29654424	29654449	29654673	29654693	hg19	TTTGTTTG	CTGGTGCA	NA	0
AMPL1215	chr17	29657135	29657164	29657375	29657404	hg19	GATTAAGC	GATACATA	NA	0
AMPL1215	chr17	29663498	29663526	29663748	29663767	hg19	ACTTTTCTT	GCATCAGC	NA	0
AMPL1215	chr17	29667283	29667308	29667528	29667549	hg19	AGAAATCA	AAAGAGG	NA	0
AMPL1215	chr17	29683873	29683905	29684113	29684135	hg19	AGTATCTA	GGGGACT	NA	0
AMPL2230	chr17	29684139	29684170	29684304	29684330	hg19	CCTCAAAT	ATTCGTTG	NA	0
AMPL1215	chr17	29685392	29685417	29685632	29685661	hg19	TAATTTTG	TCTCCAGA	NA	0
AMPL1812	chr17	40474240	40474265	40474489	40474509	hg19	ATCCCAAA	AGACCCAG	NA	0
AMPL1215	chr17	40474823	40474845	40475069	40475092	hg19	CTGGAGCA	CCTGCTAA	NA	0
AMPL2412	chr17	40475099	40475120	40475331	40475352	hg19	CCTGGAGC	CTTCTCCT	NA	0
AMPL2339	chr17	58740338	58740365	58740584	58740607	hg19	TACCTTCTT	TGTTGAAT	NA	0
AMPL2358	chr17	58740627	58740653	58740811	58740836	hg19	AAAATTTG	GCATGGTG	NA	0
AMPL1215	chr18	42530222	42530241	42530436	42530456	hg19	CTGAGTGC	CTGGCTGG	NA	0
AMPL1215	chr18	42531778	42531798	42532022	42532040	hg19	GGAACCTG	TTTGTGCT	NA	0
AMPL1107	chr18	42532445	42532467	42532693	42532714	hg19	ACTATGCA	CCTTGGGA	NA	0
AMPL1377	chr18	42532955	42532975	42533165	42533187	hg19	CCGGCCAC	GTCACTGT	NA	0
AMPL1377	chr18	42533188	42533210	42533355	42533377	hg19	GTGAGTGC	AGATGTGT	NA	0
AMPL1643	chr19	8976121	8976143	8976298	8976318	hg19	AGGCAGG	CCATGGTG	NA	0
AMPL2358	chr19	8981980	8982009	8982203	8982223	hg19	ATCTGTAA	CAGCAGAC	NA	0
AMPL2358	chr19	8999243	8999270	8999488	8999509	hg19	ATAGAAAC	GATCCCAA	NA	0
AMPL2339	chr19	9028128	9028153	9028374	9028395	hg19	ATTTTCAA	CCAGAGA	NA	0
AMPL1377	chr19	13054383	13054405	13054628	13054652	hg19	CATCACCA	CTCATCTT	NA	0
AMPL2441	chr19	33792251	33792270	33792438	33792456	hg19	CAGTTGCC	CGCGAGC	NA	1
AMPL2358	chr20	31021004	31021033	31021252	31021273	hg19	TGTTTCTG	CAGATTCC	NA	0
AMPL2358	chr20	31022041	31022061	31022291	31022310	hg19	ACAGCCCT	GGATGATC	NA	0
AMPL2358	chr20	31022351	31022373	31022594	31022613	hg19	GACCCTCG	AAGGCGG	NA	1
AMPL1215	chr20	31022689	31022710	31022937	31022958	hg19	GAAAGGA	GAATGGG	NA	0
AMPL2358	chr20	31022959	31022986	31023132	31023159	hg19	TGTCTCTA	GTCTAGTA	NA	0
AMPL1215	chr20	31023167	31023188	31023411	31023431	hg19	CTTGAAAA	GCCATTGC	NA	0
AMPL5583	chr20	31023499	31023518	31023732	31023753	hg19	ACGGTGAC	GGGAATCT	NA	0
AMPL1215	chr20	31023774	31023793	31024021	31024042	hg19	TACCAGCC	GGCTCCTT	NA	0
AMPL1832	chr20	31024113	31024134	31024344	31024367	hg19	CCCCAAAA	TGGACCA	NA	0
AMPL1215	chr20	31024537	31024558	31024784	31024806	hg19	GCACAAAC	GGAGAGA	NA	0
AMPL1215	chr20	31024807	31024828	31025035	31025055	hg19	CCTCCCAA	TCATGGCT	NA	0
AMPL2441	chr20	57428943	57428963	57429160	57429178	hg19	CAAAGGCT	TTGGGATG	NA	1
AMPL1668	chr20	57484188	57484209	57484422	57484446	hg19	CACCCCAC	CTCAAAGA	NA	0
AMPL2441	chr20	57484901	57484921	57485135	57485157	hg19	GCCCTGGT	AAGAGTG	NA	0
AMPL1378	chr21	36164620	36164642	36164826	36164844	hg19	CCATGGAG	ACCCCCGC	NA	1
AMPL2358	chr21	36171431	36171458	36171674	36171695	hg19	ATGTATCC	CATTGCCT	NA	0
AMPL1215	chr21	36193742	36193767	36193990	36194010	hg19	TTAATCCT	ACACATGG	NA	0
AMPL1823	chr21	36206682	36206702	36206893	36206913	hg19	GCAGTGG	TGCTCCCC	NA	0
AMPL1378	chr21	36231550	36231574	36231792	36231815	hg19	CTTTTGCC	ACAGAGC	NA	0
AMPL1378	chr21	36252712	36252741	36252940	36252963	hg19	TTTTTAAG	GGCTGGC	NA	0
AMPL1215	chr21	36259133	36259154	36259324	36259342	hg19	CCAGTACC	GCAAGATC	NA	1
AMPL1378	chr21	36420930	36420959	36421175	36421198	hg19	TTTATTAA	CGATGGCT	NA	0
AMPL1108	chr21	39755588	39755609	39755837	39755857	hg19	GGAAGTC	TGACCTCA	NA	0
AMPL2412	chr21	44513050	44513079	44513299	44513319	hg19	TAAGTTAC	TCTAGAGA	NA	0
AMPL1138	chr21	44514532	44514553	44514781	44514801	hg19	AGGGGAG	ACTTCAGA	NA	0
AMPL2412	chr21	44514823	44514842	44514986	44515014	hg19	CGTGGATC	CTATTCCA	NA	0
AMPL2231	chr21	44524253	44524274	44524487	44524516	hg19	CTGGAAGT	ACAGAGTC	NA	0
AMPL2264	chr22	29083722	29083742	29083962	29083989	hg19	GCACAAAC	TTCCTTTAT	NA	0
AMPL2358	chr22	29091620	29091643	29091841	29091869	hg19	GACAACAC	AATTTTAG	NA	0
AMPL1215	chrX	15808876	15808896	15809123	15809145	hg19	CTGTCTGG	GTCCATCA	NA	0
AMPL1139	chrX	15817775	15817808	15818015	15818040	hg19	TGATTAAT	TGTTCTTC	NA	0
AMPL1378	chrX	15821651	15821674	15821898	15821920	hg19	TGCTCTTC	CCTCTTGT	NA	0
AMPL2231	chrX	15822080	15822109	15822319	15822341	hg19	AAAAAGAC	ACATCCCA	NA	0
AMPL1378	chrX	15826169	15826199	15826402	15826431	hg19	AAAAGAG	TTGGTCTC	NA	0
AMPL1378	chrX	15827250	15827283	15827433	15827465	hg19	TTAATATA	ACATGATA	NA	0

AMPL2358	chrX	15833718	15833740	15833960	15833979	hg19	CCATGCCT(	TCGGGCAA	NA	0
AMPL1215	chrX	15838171	15838191	15838419	15838440	hg19	GCTTTGGG	CCACAAAT	NA	0
AMPL1378	chrX	15840703	15840730	15840948	15840972	hg19	ACATCAAT	CCGATCTG	NA	0
AMPL1215	chrX	15841060	15841081	15841309	15841329	hg19	AACCCTAG	ATTACCCG	NA	0
AMPL2413	chrX	39911422	39911445	39911657	39911683	hg19	CAGAGTCT	ACTCAAAG	NA	0
AMPL1378	chrX	39912910	39912933	39913153	39913179	hg19	AGTGATGA	CTCTTACC(	NA	0
AMPL2231	chrX	39913263	39913292	39913510	39913532	hg19	CTAAAACA	ACTTCTAT(	NA	0
AMPL1215	chrX	39914509	39914534	39914756	39914777	hg19	AAGCTTTA	TCTCTTCCT	NA	0
AMPL1215	chrX	39916294	39916315	39916535	39916563	hg19	GTCACCCT(	GTACTGCT	NA	0
AMPL1378	chrX	39921256	39921274	39921504	39921524	hg19	ATCCTGTT(	ACAGCTGC	NA	1
AMPL1215	chrX	39922003	39922022	39922245	39922264	hg19	CCATCGGC	GCAAAAA(	NA	0
AMPL1215	chrX	39922851	39922872	39923088	39923113	hg19	CCCCGCAT	GAATTAAC	NA	0
AMPL1215	chrX	39923114	39923134	39923293	39923313	hg19	AATGCACA	AGAGACCA	NA	0
AMPL1215	chrX	39923467	39923487	39923709	39923727	hg19	CTTCCCCT(	TCGGACAT	NA	1
AMPL2231	chrX	39930701	39930730	39930940	39930968	hg19	TGAAGGTA	TTGAGTGC	NA	0
AMPL1378	chrX	39931428	39931448	39931678	39931697	hg19	TCAGTGCT	AACTCAGC	NA	0
AMPL2231	chrX	39931698	39931718	39931909	39931929	hg19	GGCGATTC	GAACCTAG	NA	0
AMPL1215	chrX	39932264	39932287	39932513	39932533	hg19	CATCTGGA	GCAGTCTG	NA	0
AMPL1215	chrX	39932548	39932568	39932798	39932817	hg19	ACTGGGTG	TATTCAGC	NA	0
AMPL2231	chrX	39932818	39932839	39933037	39933060	hg19	ACGGATGC	GCCAAGTC	NA	0
AMPL1139	chrX	39933093	39933123	39933339	39933361	hg19	GATGATTT	CAGCGGTT	NA	0
AMPL1215	chrX	39933652	39933672	39933872	39933891	hg19	GGAACCCT	AGTCTGCA	NA	0
AMPL1215	chrX	39934179	39934200	39934426	39934448	hg19	TTTTCCAG	TGTTCTTCT	NA	0
AMPL1215	chrX	44820414	44820443	44820650	44820683	hg19	TTTTCAATA	CCAACACA	NA	0
AMPL2231	chrX	44833736	44833763	44833974	44833998	hg19	ACCATGGT	AACACAAA	NA	0
AMPL1215	chrX	44879826	44879858	44880035	44880068	hg19	TAAGATAT	CCAACATT	NA	0
AMPL1215	chrX	44896671	44896698	44896915	44896940	hg19	ATAATGAC	ACTTACCT(	NA	0
AMPL1389	chrX	44910801	44910832	44911017	44911043	hg19	TGTTAAAG	CTGTTGTA	NA	0
AMPL2231	chrX	44912917	44912947	44913148	44913170	hg19	GAATTGGC	TTAGGATC	NA	0
AMPL1389	chrX	44918149	44918178	44918338	44918365	hg19	AAAAATTT	TTATACAA	NA	0
AMPL1215	chrX	44919133	44919160	44919382	44919402	hg19	GTCTTCTA	CCTGCTGT	NA	0
AMPL1389	chrX	44921717	44921745	44921960	44921986	hg19	TTCTCAAG	TGTTGCAT	NA	0
AMPL1215	chrX	44922797	44922817	44923036	44923063	hg19	TCTCTCAG	CCTGAGTG	NA	0
AMPL1389	chrX	44928929	44928952	44929171	44929190	hg19	CAGAATCA	GGCTAGGA	NA	0
AMPL1215	chrX	44929240	44929261	44929487	44929509	hg19	CTCTGCCT	AGATCTGT	NA	0
AMPL2413	chrX	44935806	44935827	44936014	44936034	hg19	TGGATCCA	GGCAAGG(	NA	0
AMPL1215	chrX	44938199	44938220	44938447	44938468	hg19	GATTCAGG	GCAACAAC	NA	0
AMPL1215	chrX	44942614	44942638	44942844	44942872	hg19	TCTGATTG	GTTAGTTC	NA	0
AMPL2442	chrX	44948904	44948932	44949144	44949167	hg19	AAAAATTT	GGACCAAC	NA	0
AMPL1389	chrX	44949848	44949877	44950068	44950093	hg19	TTATAGTG	GCTTTGGA	NA	0
AMPL1389	chrX	44966473	44966492	44966717	44966741	hg19	AGGCCTGC	CGCCCATG	NA	0
AMPL1215	chrX	44969244	44969275	44969480	44969513	hg19	TAGAATTC	ACATGTTG	NA	0
AMPL1670	chrX	53408951	53408971	53409199	53409220	hg19	TGCTGAGC	CCTATGGA	NA	0
AMPL1215	chrX	53423306	53423325	53423544	53423564	hg19	AAAGGTCC	TTGTCCTG	NA	0
AMPL1215	chrX	53426368	53426389	53426618	53426637	hg19	TTTTCCCGT	TCAGAAGA	NA	0
AMPL1930	chrX	53431778	53431798	53432016	53432036	hg19	GAGCCAAC	CAGTCTCA	NA	0
AMPL1833	chrX	53432340	53432363	53432580	53432605	hg19	GAAGAGA(	GGTGAAG(	NA	0
AMPL2413	chrX	53441692	53441712	53441942	53441961	hg19	GTTAAAAA	TGAGGACC	NA	0
AMPL1215	chrX	1.23E+08	1.23E+08	1.23E+08	1.23E+08	hg19	GTTATGTA	ACATAAAT	NA	0
AMPL1379	chrX	1.23E+08	1.23E+08	1.23E+08	1.23E+08	hg19	AGGTCATT	GGTCAAGA	NA	0
AMPL1216	chrX	1.23E+08	1.23E+08	1.23E+08	1.23E+08	hg19	TAGAGTAT	AAGTTATC	NA	0
AMPL1379	chrX	1.23E+08	1.23E+08	1.23E+08	1.23E+08	hg19	AGTCATGC	AATATTTCT	NA	0
AMPL2231	chrX	1.23E+08	1.23E+08	1.23E+08	1.23E+08	hg19	TTGTGTCT(	AGTTATAT(	NA	0
AMPL2231	chrX	1.23E+08	1.23E+08	1.23E+08	1.23E+08	hg19	GCATTTGG	GCTCAACT	NA	0
AMPL1379	chrX	1.23E+08	1.23E+08	1.23E+08	1.23E+08	hg19	TTTTTAAAT	GTTTGATT	NA	0
AMPL1216	chrX	1.23E+08	1.23E+08	1.23E+08	1.23E+08	hg19	ATTTTCTAT	AATACATA	NA	0
AMPL1379	chrX	1.23E+08	1.23E+08	1.23E+08	1.23E+08	hg19	CAGATTTCT	CTCAACCA	NA	0
AMPL2413	chrX	1.29E+08	1.29E+08	1.29E+08	1.29E+08	hg19	CTGGGTGA	GCAATGCG	NA	0
AMPL1811	chrX	1.29E+08	1.29E+08	1.29E+08	1.29E+08	hg19	CTCATGCCT	TACCTTCCT	NA	0
AMPL2210	chrX	1.29E+08	1.29E+08	1.29E+08	1.29E+08	hg19	GAAGAAGA	AGTCTGAT	NA	0
AMPL1379	chrX	1.34E+08	1.34E+08	1.34E+08	1.34E+08	hg19	ACTGATTT	TTGCTGCC	NA	0
AMPL1379	chrX	1.34E+08	1.34E+08	1.34E+08	1.34E+08	hg19	ACTTTTCA	TGTAAATT(	NA	0
AMPL2231	chrX	1.34E+08	1.34E+08	1.34E+08	1.34E+08	hg19	CCATTTCA	GCTGAAAC	NA	0
AMPL2231	chrX	1.34E+08	1.34E+08	1.34E+08	1.34E+08	hg19	ATTGGGTG	GGTATCTT(	NA	0
AMPL1216	chrX	1.34E+08	1.34E+08	1.34E+08	1.34E+08	hg19	GGTAAAGT	GCTTAAAA	NA	0
AMPL1258	chrX	1.34E+08	1.34E+08	1.34E+08	1.34E+08	hg19	TCTTTCTTC	GCAAATCA	NA	0
AMPL1216	chrX	1.34E+08	1.34E+08	1.34E+08	1.34E+08	hg19	ATCAAAGT	CTTGTAAA	NA	0
AMPL1216	chrX	1.34E+08	1.34E+08	1.34E+08	1.34E+08	hg19	AGTGTGGA	TCCCATATA	NA	0

Supplemental Table S3. Panel Targets

Target	chr	start	end	bases_covered	bases_missed	coverage_perc	Amino acid	Exon	Gene name
**chr1:1737914-1737916**	chr1	1737914	1737916	3	0	100	K89	6	GNB1
**chr1:1737941-1737943**	chr1	1737941	1737943	3	0	100	I80	6	GNB1
**chr1:1737953-1737955**	chr1	1737953	1737955	3	0	100	D76	6	GNB1
**chr1:36932213-36932213**	chr1	36932213	36932213	1	0	100	Y779	17	CSF3R
**chr1:36932224-36932224**	chr1	36932224	36932224	1	0	100	Q776	17	CSF3R
**chr1:36932242-36932242**	chr1	36932242	36932242	1	0	100	Q770	17	CSF3R
**chr1:36932248-36932248**	chr1	36932248	36932248	1	0	100	Q768	17	CSF3R
**chr1:36932254-36932254**	chr1	36932254	36932254	1	0	100	Q766	17	CSF3R
**chr1:36932341-36932341**	chr1	36932341	36932341	1	0	100	E737	17	CSF3R
**chr1:36932400-36932400**	chr1	36932400	36932400	1	0	100	T717	17	CSF3R
**chr1:36932463-36932463**	chr1	36932463	36932463	1	0	100	M696	17	CSF3R
**chr1:36932503-36932503**	chr1	36932503	36932503	1	0	100	G683	17	CSF3R
**chr1:36933198-36933198**	chr1	36933198	36933198	1	0	100	T640	15	CSF3R
**chr1:36933216-36933216**	chr1	36933216	36933216	1	0	100	G634	15	CSF3R
**chr1:36933246-36933246**	chr1	36933246	36933246	1	0	100	S624	15	CSF3R
**chr1:36933434-36933434**	chr1	36933434	36933434	1	0	100	T618	14	CSF3R
**chr1:36933444-36933444**	chr1	36933444	36933444	1	0	100	T615	14	CSF3R
**chr1:36933539-36933539**	chr1	36933539	36933539	1	0	100	R583	14	CSF3R
**chr1:43814979-43814979**	chr1	43814979	43814979	1	0	100	S505	10	MPL
**chr1:43815008-43815008**	chr1	43815008	43815008	1	0	100	W515	10	MPL
**chr1:43815009-43815009**	chr1	43815009	43815009	1	0	100	W515	10	MPL
**chr1:115252202-11525220**	chr1	115252205	115252207	3	0	100	S146	4	NRAS
**chr1:115256528-11525652**	chr1	115256528	115256529	2	0	100	Q61	3	NRAS
**chr1:115256528-11525653**	chr1	115256528	115256530	3	0	100	Q61	3	NRAS
**chr1:115256532-11525653**	chr1	115256532	115256532	1	0	100	G60	3	NRAS
**chr1:115256534-11525653**	chr1	115256534	115256536	3	0	100	A59	3	NRAS
**chr1:115258730-11525873**	chr1	115258730	115258730	1	0	100	A18	2	NRAS
**chr1:115258744-11525874**	chr1	115258744	115258744	1	0	100	G13	2	NRAS
**chr1:115258745-11525874**	chr1	115258745	115258745	1	0	100	G13	2	NRAS
**chr1:115258747-11525874**	chr1	115258747	115258747	1	0	100	G12	2	NRAS
**chr1:115258748-11525874**	chr1	115258748	115258748	1	0	100	G12	2	NRAS
**chr2:25457158-25457158**	chr2	25457158	25457158	1	0	100	A910	23	DNMT3A
**chr2:25457161-25457161**	chr2	25457161	25457161	1	0	100	F909	23	DNMT3A
**chr2:25457176-25457176**	chr2	25457176	25457176	1	0	100	P904	23	DNMT3A
**chr2:25457209-25457209**	chr2	25457209	25457209	1	0	100	W893	23	DNMT3A
**chr2:25457240-25457246**	chr2	25457240	25457246	7	0	100	R882	23	DNMT3A
**chr2:25457241-25457243**	chr2	25457241	25457243	3	0	100	R882	23	DNMT3A
**chr2:25458593-25458593**	chr2	25458593	25458593	1	0	100	W860	22	DNMT3A
**chr2:25458595-25458595**	chr2	25458595	25458595	1	0	100	W860	22	DNMT3A
**chr2:25458649-25458649**	chr2	25458649	25458649	1	0	100	Q842	22	DNMT3A
**chr2:25458661-25458661**	chr2	25458661	25458661	1	0	100	N832	22	DNMT3A
**chr2:25458669-25458669**	chr2	25458669	25458669	1	0	100	T835	22	DNMT3A
**chr2:25459821-25459821**	chr2	25459821	25459821	1	0	100	H821	21	DNMT3A
**chr2:25459824-25459824**	chr2	25459824	25459824	1	0	100	E820	21	DNMT3A

**chr2:25459840-25459840**	chr2	25459840	25459840	1	0	100	L815	21	DNMT3A
**chr2:25462000-25462000**	chr2	25462000	25462000	1	0	100	R803	20	DNMT3A
**chr2:25462006-25462006**	chr2	25462006	25462006	1	0	100	M801	20	DNMT3A
**chr2:25462011-25462011**	chr2	25462011	25462011	1	0	100	P799	20	DNMT3A
**chr2:25462032-25462032**	chr2	25462032	25462032	1	0	100	R792	20	DNMT3A
**chr2:25462068-25462068**	chr2	25462068	25462068	1	0	100	I780	20	DNMT3A
**chr2:25463182-25463182**	chr2	25463182	25463182	1	0	100	R771	19	DNMT3A
**chr2:25463183-25463185**	chr2	25463183	25463185	3	0	100	S770	19	DNMT3A
**chr2:25463237-25463239**	chr2	25463237	25463239	3	0	100	F752	19	DNMT3A
**chr2:25463248-25463248**	chr2	25463248	25463248	1	0	100	R749	19	DNMT3A
**chr2:25463286-25463286**	chr2	25463286	25463286	1	0	100	R736	19	DNMT3A
**chr2:25463289-25463289**	chr2	25463289	25463289	1	0	100	Y735	19	DNMT3A
**chr2:25463297-25463299**	chr2	25463297	25463299	3	0	100	F732	19	DNMT3A
**chr2:25463308-25463308**	chr2	25463308	25463308	1	0	100	R729	19	DNMT3A
**chr2:25463510-25463510**	chr2	25463510	25463510	1	0	100	Y724	18	DNMT3A
**chr2:25463532-25463532**	chr2	25463532	25463532	1	0	100	N717	18	DNMT3A
**chr2:25463536-25463536**	chr2	25463536	25463536	1	0	100	V716	18	DNMT3A
**chr2:25463541-25463541**	chr2	25463541	25463541	1	0	100	S714	18	DNMT3A
**chr2:25463553-25463553**	chr2	25463553	25463553	1	0	100	C710	18	DNMT3A
**chr2:25463568-25463568**	chr2	25463568	25463568	1	0	100	I705	18	DNMT3A
**chr2:25463586-25463586**	chr2	25463586	25463586	1	0	100	G699	18	DNMT3A
**chr2:25464444-25464444**	chr2	25464444	25464444	1	0	100	V690	17	DNMT3A
**chr2:25464450-25464450**	chr2	25464450	25464450	1	0	100	R688	17	DNMT3A
**chr2:25464451-25464451**	chr2	25464451	25464451	1	0	100	R688	17	DNMT3A
**chr2:25464460-25464460**	chr2	25464460	25464460	1	0	100	G685	17	DNMT3A
**chr2:25466797-25466797**	chr2	25466797	25466797	1	0	100	V636	16	DNMT3A
**chr2:25466799-25466799**	chr2	25466799	25466799	1	0	100	R635	16	DNMT3A
**chr2:25466800-25466800**	chr2	25466800	25466800	1	0	100	R635	16	DNMT3A
**chr2:25467059-25467059**	chr2	25467059	25467059	1	0	100	Q606	15	DNMT3A
**chr2:25467083-25467083**	chr2	25467083	25467083	1	0	100	R598	15	DNMT3A
**chr2:25467132-25467132**	chr2	25467132	25467132	1	0	100	W581	15	DNMT3A
**chr2:25467134-25467134**	chr2	25467134	25467134	1	0	100	W581	15	DNMT3A
**chr2:25467168-25467170**	chr2	25467168	25467170	3	0	100	P569	15	DNMT3A
**chr2:25467188-25467188**	chr2	25467188	25467188	1	0	100	V563	15	DNMT3A
**chr2:25467426-25467428**	chr2	25467426	25467428	3	0	100	G550	14	DNMT3A
**chr2:25467435-25467437**	chr2	25467435	25467437	3	0	100	L547	14	DNMT3A
**chr2:25467449-25467449**	chr2	25467449	25467449	1	0	100	G543	14	DNMT3A
**chr2:25467478-25467478**	chr2	25467478	25467478	1	0	100	Y533	14	DNMT3A
**chr2:25468137-25468137**	chr2	25468137	25468137	1	0	100	M513	13	DNMT3A
**chr2:25468163-25468163**	chr2	25468163	25468163	1	0	100	E505	13	DNMT3A
**chr2:25468174-25468174**	chr2	25468174	25468174	1	0	100	N501	13	DNMT3A
**chr2:25468892-25468892**	chr2	25468892	25468892	1	0	100	E491	12	DNMT3A
**chr2:25468912-25468912**	chr2	25468912	25468912	0	1	0	R484	12	DNMT3A
**chr2:25469028-25469028**	chr2	25469028	25469028	1	0	100	Non-coding		DNMT3A
**chr2:25469065-25469065**	chr2	25469065	25469065	1	0	100	P465	11	DNMT3A
**chr2:25469068-25469068**	chr2	25469068	25469068	1	0	100	K464	11	DNMT3A
**chr2:25469114-25469114**	chr2	25469114	25469114	1	0	100	Y448	11	DNMT3A

**chr2:25469138-25469138**	chr2	25469138	25469138	1	0	100	W440	11	DNMT3A
**chr2:25469158-25469158**	chr2	25469158	25469158	1	0	100	E434	11	DNMT3A
**chr2:25469540-25469540**	chr2	25469540	25469540	1	0	100	A410	10	DNMT3A
**chr2:25469541-25469541**	chr2	25469541	25469541	1	0	100	W409	10	DNMT3A
**chr2:25469564-25469564**	chr2	25469564	25469564	1	0	100	Q402	10	DNMT3A
**chr2:25469630-25469630**	chr2	25469630	25469630	1	0	100	A380	10	DNMT3A
**chr2:25469958-25469958**	chr2	25469958	25469958	1	0	100	Q362	9	DNMT3A
**chr2:25469976-25469976**	chr2	25469976	25469976	1	0	100	Q356	9	DNMT3A
**chr2:25469984-25469984**	chr2	25469984	25469984	1	0	100	A353	9	DNMT3A
**chr2:25470011-25470011**	chr2	25470011	25470011	1	0	100	L344	9	DNMT3A
**chr2:25470516-25470516**	chr2	25470516	25470516	1	0	100	R320	8	DNMT3A
**chr2:25470535-25470535**	chr2	25470535	25470535	1	0	100	W313	8	DNMT3A
**chr2:25470553-25470555**	chr2	25470553	25470555	3	0	100	P307	8	DNMT3A
**chr2:25470559-25470559**	chr2	25470559	25470559	1	0	100	W305	8	DNMT3A
**chr2:25470612-25470612**	chr2	25470612	25470612	1	0	100	R288	8	DNMT3A
**chr2:25470968-25470968**	chr2	25470968	25470968	1	0	100	V265	7	DNMT3A
**chr2:25471070-25471070**	chr2	25471070	25471070	1	0	100	Q231	7	DNMT3A
**chr2:25471094-25471094**	chr2	25471094	25471094	1	0	100	G223	7	DNMT3A
**chr2:25471109-25471109**	chr2	25471109	25471109	1	0	100	A218	7	DNMT3A
**chr2:25505393-25505393**	chr2	25505393	25505393	1	0	100	A122	4	DNMT3A
**chr2:25505402-25505402**	chr2	25505402	25505402	1	0	100	E119	4	DNMT3A
**chr2:25505463-25505463**	chr2	25505463	25505463	1	0	100	P99	4	DNMT3A
**chr2:25505484-25505484**	chr2	25505484	25505484	1	0	100	D92	4	DNMT3A
**chr2:25536789-25536789**	chr2	25536789	25536789	1	0	100	D22	2	DNMT3A
**chr2:25536799-25536799**	chr2	25536799	25536799	1	0	100	R19	2	DNMT3A
**chr2:25536849-25536849**	chr2	25536849	25536849	1	0	100	P2	2	DNMT3A
**chr2:179406190-17940619**	chr2	179406190	179406192	3	0	100	R32538	350	TTN
**chr2:179428314-17942831**	chr2	179428314	179428316	3	0	100	R27515	326	TTN
**chr2:179436018-17943602**	chr2	179436018	179436020	3	0	100	R24947	326	TTN
**chr2:179457146-17945714**	chr2	179457146	179457148	3	0	100	P19862	301	TTN
**chr2:179458590-17945859**	chr2	179458590	179458592	3	0	100	R19479	298	TTN
**chr2:179474667-17947466**	chr2	179474667	179474669	3	0	100	A17161	272	TTN
**chr2:179476543-17947654**	chr2	179476543	179476545	3	0	100	E16831	268	TTN
**chr2:179644853-17964485**	chr2	179644853	179644855	3	0	100	K1201	22	TTN
**chr2:198265475-19826547**	chr2	198265475	198265477	3	0	100	D894	18	SF3B1
**chr2:198266137-19826613**	chr2	198266137	198266137	1	0	100	R828	17	SF3B1
**chr2:198266185-19826618**	chr2	198266185	198266186	2	0	100	P812	17	SF3B1
**chr2:198266193-19826619**	chr2	198266193	198266193	1	0	100	E809	17	SF3B1
**chr2:198266493-19826649**	chr2	198266493	198266495	3	0	100	D781	16	SF3B1
**chr2:198266512-19826651**	chr2	198266512	198266512	1	0	100	R775	16	SF3B1
**chr2:198266518-19826651**	chr2	198266518	198266518	1	0	100	L773	16	SF3B1
**chr2:198266584-19826658**	chr2	198266584	198266584	0	1	0	G751	16	SF3B1
**chr2:198266606-19826660**	chr2	198266606	198266606	0	1	0	A744	16	SF3B1
**chr2:198266611-19826661**	chr2	198266611	198266611	1	0	100	G742	16	SF3B1
**chr2:198266709-19826671**	chr2	198266709	198266711	3	0	100	K741	15	SF3B1
**chr2:198266712-19826671**	chr2	198266712	198266714	3	0	100	G740	15	SF3B1
**chr2:198266768-19826676**	chr2	198266768	198266768	1	0	100	E722	15	SF3B1

**chr2:198266822-19826682**	chr2	198266822	198266822	1	0	100	I704	15	SF3B1
**chr2:198266828-19826682**	chr2	198266828	198266828	1	0	100	R702	15	SF3B1
**chr2:198266831-19826683**	chr2	198266831	198266831	1	0	100	V701	15	SF3B1
**chr2:198266831-19826683**	chr2	198266831	198266837	6	1	85.71428571	K700	15	SF3B1
**chr2:198266834-19826683**	chr2	198266834	198266834	1	0	100	K700	15	SF3B1
**chr2:198267359-19826735**	chr2	198267359	198267359	1	0	100	K666	14	SF3B1
**chr2:198267369-19826736**	chr2	198267369	198267369	1	0	100	T663	14	SF3B1
**chr2:198267371-19826737**	chr2	198267371	198267371	1	0	100	H662	14	SF3B1
**chr2:198267481-19826748**	chr2	198267481	198267481	1	0	100	N626	14	SF3B1
**chr2:198267484-19826748**	chr2	198267484	198267484	1	0	100	R625	14	SF3B1
**chr2:198267489-19826748**	chr2	198267489	198267489	1	0	100	Y623	14	SF3B1
**chr2:198267491-19826749**	chr2	198267491	198267491	1	0	100	E622	14	SF3B1
**chr2:198267695-19826769**	chr2	198267695	198267695	1	0	100	E595	13	SF3B1
**chr2:198267698-19826769**	chr2	198267698	198267698	1	0	100	R594	13	SF3B1
**chr2:198267705-19826770**	chr2	198267705	198267705	1	0	100	E592	13	SF3B1
**chr2:198267710-19826771**	chr2	198267710	198267710	1	0	100	R590	13	SF3B1
**chr2:198267752-19826775**	chr2	198267752	198267752	1	0	100	V576	13	SF3B1
**chr2:209113112-20911311**	chr2	209113112	209113112	1	0	100	R132	4	IDH1
**chr2:209113113-20911311**	chr2	209113113	209113113	1	0	100	R132	4	IDH1
**chr2:209113208-20911320**	chr2	209113208	209113208	1	0	100	R100	4	IDH1
**chr2:209113298-20911329**	chr2	209113298	209113298	1	0	100	G70	4	IDH1
**chr3:38182259-38182259**	chr3	38182259	38182259	1	0	100	M232	4	MYD88
**chr3:38182283-38182283**	chr3	38182283	38182283	1	0	100	Y240	4	MYD88
**chr3:38182292-38182292**	chr3	38182292	38182292	1	0	100	S243	4	MYD88
**chr3:38182727-38182727**	chr3	38182727	38182727	1	0	100	T294	5	MYD88
**chr3:38182744-38182744**	chr3	38182744	38182744	1	0	100	W299	5	MYD88
**chr3:38182748-38182748**	chr3	38182748	38182748	1	0	100	R301	5	MYD88
**chr3:38182749-38182749**	chr3	38182749	38182749	1	0	100	R301	5	MYD88
**chr3:128200691-12820069**	chr3	128200691	128200691	1	0	100	A372	6	GATA2
**chr3:128200720-12820072**	chr3	128200720	128200720	1	0	100	R362	6	GATA2
**chr3:128200723-12820072**	chr3	128200723	128200723	1	0	100	R361	6	GATA2
**chr3:128200730-12820073**	chr3	128200730	128200730	1	0	100	L359	6	GATA2
**chr3:128202731-12820273**	chr3	128202731	128202731	1	0	100	R330	5	GATA2
**chr3:128202753-12820275**	chr3	128202753	128202753	1	0	100	H323	5	GATA2
**chr3:128202759-12820275**	chr3	128202759	128202759	1	0	100	L321	5	GATA2
**chr3:128202761-12820276**	chr3	128202761	128202761	1	0	100	G320	5	GATA2
**chr3:128202767-12820276**	chr3	128202767	128202767	1	0	100	A318	5	GATA2
**chr4:55561756-55561756**	chr4	55561756	55561756	1	0	100	R49	2	KIT
**chr4:55561758-55561758**	chr4	55561758	55561758	1	0	100	V50	2	KIT
**chr4:55561764-55561764**	chr4	55561764	55561764	1	0	100	D52	2	KIT
**chr4:55561767-55561767**	chr4	55561767	55561767	1	0	100	E53	2	KIT
**chr4:55561801-55561801**	chr4	55561801	55561801	1	0	100	V64	2	KIT
**chr4:55589771-55589773**	chr4	55589771	55589773	3	0	100	Y418	8	KIT
**chr4:55589772-55589772**	chr4	55589772	55589772	1	0	100	Y418	8	KIT
**chr4:55589773-55589773**	chr4	55589773	55589773	1	0	100	Y418	9	KIT
**chr4:55589773-55589778**	chr4	55589773	55589778	6	0	100	D419	9	KIT
**chr4:55589774-55589774**	chr4	55589774	55589774	1	0	100	D419	9	KIT

**chr4:55592079-55592079**	chr4	55592079	55592079	1	0	100	P468	9	KIT
**chr4:55592103-55592103**	chr4	55592103	55592103	1	0	100	S476	9	KIT
**chr4:55592144-55592144**	chr4	55592144	55592144	1	0	100	E490	9	KIT
**chr4:55592202-55592202**	chr4	55592202	55592202	1	0	100	K509	9	KIT
**chr4:55592212-55592212**	chr4	55592212	55592212	1	0	100	N512	9	KIT
**chr4:55593464-55593466**	chr4	55593464	55593466	3	0	100	M541	10	KIT
**chr4:55593603-55593605**	chr4	55593603	55593605	3	0	100	W557	11	KIT
**chr4:55593609-55593611**	chr4	55593609	55593611	3	0	100	V559	11	KIT
**chr4:55593613-55593613**	chr4	55593613	55593613	1	0	100	V560	11	KIT
**chr4:55593627-55593627**	chr4	55593627	55593627	1	0	100	G565	11	KIT
**chr4:55593648-55593648**	chr4	55593648	55593648	1	0	100	D572	11	KIT
**chr4:55593652-55593652**	chr4	55593652	55593652	1	0	100	P573	11	KIT
**chr4:55593660-55593660**	chr4	55593660	55593660	1	0	100	L576	11	KIT
**chr4:55593661-55593661**	chr4	55593661	55593661	1	0	100	L576	11	KIT
**chr4:55593663-55593663**	chr4	55593663	55593663	1	0	100	P577	11	KIT
**chr4:55593667-55593669**	chr4	55593667	55593669	3	0	100	Y578	11	KIT
**chr4:55593691-55593691**	chr4	55593691	55593691	0	1	0	R586	11	KIT
**chr4:55594197-55594197**	chr4	55594197	55594197	1	0	100	R634	13	KIT
**chr4:55594221-55594221**	chr4	55594221	55594221	1	0	100	K642	13	KIT
**chr4:55594258-55594258**	chr4	55594258	55594258	1	0	100	V654	13	KIT
**chr4:55594262-55594262**	chr4	55594262	55594262	1	0	100	N655	13	KIT
**chr4:55599261-55599261**	chr4	55599261	55599261	1	0	100	R796	17	KIT
**chr4:55599321-55599321**	chr4	55599321	55599321	1	0	100	D816	17	KIT
**chr4:55599327-55599327**	chr4	55599327	55599327	1	0	100	K818	17	KIT
**chr4:55599332-55599332**	chr4	55599332	55599332	1	0	100	D820	17	KIT
**chr4:55599340-55599340**	chr4	55599340	55599340	1	0	100	N822	17	KIT
**chr4:55599341-55599341**	chr4	55599341	55599341	1	0	100	Y823	17	KIT
**chr4:55599348-55599348**	chr4	55599348	55599348	1	0	100	V825	17	KIT
**chr4:106155172-106155172**	chr4	106155172	106155172	1	0	100	C25	3	TET2
**chr4:106155185-106155185**	chr4	106155185	106155185	1	0	100	P29	3	TET2
**chr4:106155199-106155199**	chr4	106155199	106155199	1	0	100	L34	3	TET2
**chr4:106155337-106155337**	chr4	106155337	106155337	1	0	100	Q80	3	TET2
**chr4:106155421-106155421**	chr4	106155421	106155421	1	0	100	Q108	3	TET2
**chr4:106155502-106155502**	chr4	106155502	106155502	1	0	100	E135	3	TET2
**chr4:106155553-106155553**	chr4	106155553	106155553	1	0	100	S152	3	TET2
**chr4:106155652-106155652**	chr4	106155652	106155652	0	1	0	Q185	3	TET2
**chr4:106155751-106155751**	chr4	106155751	106155751	1	0	100	V218	3	TET2
**chr4:106155751-106155753**	chr4	106155751	106155753	3	0	100	V218	3	TET2
**chr4:106155764-106155764**	chr4	106155764	106155764	1	0	100	H222	3	TET2
**chr4:106155784-106155786**	chr4	106155784	106155786	3	0	100	T229	3	TET2
**chr4:106155793-106155793**	chr4	106155793	106155793	1	0	100	Q232	3	TET2
**chr4:106155883-106155885**	chr4	106155883	106155885	3	0	100	C262	3	TET2
**chr4:106155922-106155924**	chr4	106155922	106155924	3	0	100	N275	3	TET2
**chr4:106156048-106156048**	chr4	106156048	106156048	1	0	100	Q317	3	TET2
**chr4:106156060-106156060**	chr4	106156060	106156060	1	0	100	Q321	3	TET2
**chr4:106156066-106156066**	chr4	106156066	106156066	1	0	100	Q323	3	TET2
**chr4:106156069-106156069**	chr4	106156069	106156069	1	0	100	Q324	3	TET2

**chr4:106156072-10615607**	chr4	106156072	106156072	1	0	100	Q325	3	TET2	
**chr4:106156160-10615616**	chr4	106156160	106156160	1	0	100	S354	3	TET2	
**chr4:106156163-10615616**	chr4	106156163	106156163	1	0	100	G355	3	TET2	
**chr4:106156187-10615618**	chr4	106156187	106156187	1	0	100	P363	3	TET2	
**chr4:106156246-10615624**	chr4	106156246	106156246	1	0	100	Q383	3	TET2	
**chr4:106156339-10615633**	chr4	106156339	106156339	1	0	100	Q414	3	TET2	
**chr4:106156348-10615634**	chr4	106156348	106156348	1	0	100	Q417	3	TET2	
**chr4:106156478-10615647**	chr4	106156478	106156478	1	0	100	S460	3	TET2	
**chr4:106156496-10615649**	chr4	106156496	106156496	1	0	100	S466	3	TET2	
**chr4:106156540-10615654**	chr4	106156540	106156540	0	1	0	Q481	3	TET2	
**chr4:106156624-10615662**	chr4	106156624	106156626	3	0	100	S509	3	TET2	
**chr4:106156729-10615672**	chr4	106156729	106156729	1	0	100	R544	3	TET2	
**chr4:106156747-10615674**	chr4	106156747	106156747	1	0	100	R550	3	TET2	
**chr4:106156763-10615676**	chr4	106156763	106156763	1	0	100	P555	3	TET2	
**chr4:106156768-10615676**	chr4	106156768	106156768	1	0	100	Q557	3	TET2	
**chr4:106156783-10615678**	chr4	106156783	106156783	1	0	100	P562	3	TET2	
**chr4:106156942-10615694**	chr4	106156942	106156944	3	0	100	L615	3	TET2	
**chr4:106156963-10615696**	chr4	106156963	106156963	1	0	100	Q622	3	TET2	
**chr4:106157002-10615700**	chr4	106157002	106157002	1	0	100	Q635	3	TET2	
**chr4:106157053-10615705**	chr4	106157053	106157053	1	0	100	Q652	3	TET2	
**chr4:106157059-10615705**	chr4	106157059	106157059	1	0	100	Q654	3	TET2	
**chr4:106157200-10615720**	chr4	106157200	106157202	3	0	100	Q701	3	TET2	
**chr4:106157329-10615733**	chr4	106157329	106157331	3	0	100	Q744	3	TET2	
**chr4:106157331-10615733**	chr4	106157331	106157337	7	0	100	Q746	3	TET2	
**chr4:106157335-10615733**	chr4	106157335	106157337	3	0	100	Q746	3	TET2	
**chr4:106157371-10615737**	chr4	106157371	106157371	1	0	100	Q758	3	TET2	
**chr4:106157389-10615739**	chr4	106157389	106157391	3	0	100	Q764	3	TET2	
**chr4:106157404-10615740**	chr4	106157404	106157404	1	0	100	Q769	3	TET2	
**chr4:106157480-10615748**	chr4	106157480	106157480	1	0	100	Q794	3	TET2	
**chr4:106157527-10615752**	chr4	106157527	106157527	1	0	100	Q810	3	TET2	
**chr4:106157539-10615753**	chr4	106157539	106157539	1	0	100	R814	3	TET2	
**chr4:106157701-10615770**	chr4	106157701	106157701	1	0	100	F868	3	TET2	
**chr4:106157701-10615770**	chr4	106157701	106157703	3	0	100	F868	3	TET2	
**chr4:106157725-10615772**	chr4	106157725	106157725	1	0	100	Q876	3	TET2	
**chr4:106157773-10615777**	chr4	106157773	106157773	1	0	100	Q892	3	TET2	
**chr4:106157791-10615779**	chr4	106157791	106157791	1	0	100	G898	3	TET2	
**chr4:106157845-10615784**	chr4	106157845	106157847	3	0	100	Q916	3	TET2	
**chr4:106157961-10615796**	chr4	106157961	106157961	1	0	100	W954	3	TET2	
**chr4:106157971-10615797**	chr4	106157971	106157971	1	0	100	Q958	3	TET2	
**chr4:106157986-10615798**	chr4	106157986	106157986	1	0	100	Q963	3	TET2	
**chr4:106157995-10615799**	chr4	106157995	106157995	1	0	100	Q966	3	TET2	
**chr4:106158157-10615815**	chr4	106158157	106158157	1	0	100	Q1020	3	TET2	
**chr4:106158256-10615825**	chr4	106158256	106158256	1	0	100	Q1053	3	TET2	
**chr4:106158275-10615827**	chr4	106158275	106158275	1	0	100	S1059	3	TET2	
**chr4:106158301-10615830**	chr4	106158301	106158301	1	0	100	Q1068	3	TET2	
**chr4:106158346-10615834**	chr4	106158346	106158346	1	0	100	Q1083	3	TET2	
**chr4:106162516-10616251**	chr4	106162516	106162516	1	0	100	E1144	4	TET2	

**chr4:106162530-10616253**	chr4	106162530	106162530	1	0	100	Y1148	4	TET2
**chr4:106162553-10616255**	chr4	106162553	106162553	1	0	100	N1156	4	TET2
**chr4:106162576-10616257**	chr4	106162576	106162578	3	0	100	M1164	4	TET2
**chr4:106162586-10616258**	chr4	106162586	106162586	1	0	100	R1167	4	TET2
**chr4:106163998-10616399**	chr4	106163998	106163998	1	0	100	Q1170	5	TET2
**chr4:106164022-10616402**	chr4	106164022	106164022	1	0	100	Q1191	5	TET2
**chr4:106164061-10616406**	chr4	106164061	106164061	1	0	100	E1178	5	TET2
**chr4:106164067-10616406**	chr4	106164067	106164069	3	0	100	C1193	5	TET2
**chr4:106164083-10616408**	chr4	106164083	106164083	1	0	100	W1198	5	TET2
**chr4:106164772-10616477**	chr4	106164772	106164772	1	0	100	R1214	6	TET2
**chr4:106164773-10616477**	chr4	106164773	106164773	1	0	100	R1216	6	TET2
**chr4:106164778-10616477**	chr4	106164778	106164778	1	0	100	C1221	6	TET2
**chr4:106164794-10616479**	chr4	106164794	106164794	1	0	100	V1227	6	TET2
**chr4:106164812-10616481**	chr4	106164812	106164812	1	0	100	Y1245	6	TET2
**chr4:106164867-10616486**	chr4	106164867	106164867	1	0	100	Y 1255	6	TET2
**chr4:106164897-10616489**	chr4	106164897	106164897	1	0	100	R1261	6	TET2
**chr4:106164913-10616491**	chr4	106164913	106164913	1	0	100	R1261	6	TET2
**chr4:106164914-10616491**	chr4	106164914	106164914	1	0	100	R1261	6	TET2
**chr4:106164916-10616491**	chr4	106164916	106164916	1	0	100	R1262	6	TET2
**chr4:106180785-10618078**	chr4	106180785	106180785	1	0	100	C1271	7	TET2
**chr4:106180789-10618079**	chr4	106180789	106180791	3	0	100	C1273	7	TET2
**chr4:106180792-10618079**	chr4	106180792	106180794	3	0	100	Q1274	7	TET2
**chr4:106180795-10618079**	chr4	106180795	106180797	3	0	100	Q1275	7	TET2
**chr4:106180816-10618081**	chr4	106180816	106180818	3	0	100	G1282	7	TET2
**chr4:106180831-10618083**	chr4	106180831	106180832	2	0	100	F1287	7	TET2
**chr4:106180835-10618083**	chr4	106180835	106180835	1	0	100	G1288	7	TET2
**chr4:106180838-10618083**	chr4	106180838	106180838	1	0	100	C1289	7	TET2
**chr4:106180853-10618085**	chr4	106180853	106180853	1	0	100	Y1294	7	TET2
**chr4:106180865-10618086**	chr4	106180865	106180865	1	0	100	C1298	7	TET2
**chr4:106180867-10618086**	chr4	106180867	106180869	3	0	100	K1299	7	TET2
**chr4:106180925-10618092**	chr4	106180925	106180925	1	0	100	E1318	7	TET2
**chr4:106182926-10618292**	chr4	106182926	106182926	1	0	100	L1322	8	TET2
**chr4:106182928-10618292**	chr4	106182928	106182928	1	0	100	E1323	8	TET2
**chr4:106182940-10618294**	chr4	106182940	106182940	1	0	100	Q1327	8	TET2
**chr4:106182980-10618298**	chr4	106182980	106182980	1	0	100	L1340	8	TET2
**chr4:106190786-10619078**	chr4	106190786	106190786	1	0	100	A1355	9	TET2
**chr4:106190797-10619079**	chr4	106190797	106190797	1	0	100	R1359	9	TET2
**chr4:106190798-10619079**	chr4	106190798	106190798	1	0	100	R1359	9	TET2
**chr4:106190818-10619082**	chr4	106190818	106190820	3	0	100	R1366	9	TET2
**chr4:106190819-10619081**	chr4	106190819	106190819	1	0	100	R1366	9	TET2
**chr4:106190855-10619085**	chr4	106190855	106190855	1	0	100	C1378	9	TET2
**chr4:106190860-10619086**	chr4	106190860	106190860	1	0	100	H1380	9	TET2
**chr4:106190869-10619086**	chr4	106190869	106190869	1	0	100	R1383	9	TET2
**chr4:106193778-10619378**	chr4	106193778	106193780	3	0	100	Q1414	10	TET2
**chr4:106193787-10619378**	chr4	106193787	106193789	3	0	100	V1417	10	TET2
**chr4:106193892-10619389**	chr4	106193892	106193894	3	0	100	R1452	10	TET2
**chr4:106193931-10619393**	chr4	106193931	106193931	1	0	100	R1465	10	TET2

**chr4:106193995-10619399**	chr4	106193995	106193995	1	0	100	S1486	10	TET2
**chr4:106194051-10619405**	chr4	106194051	106194051	0	1	0	A1505	11	TET2
**chr4:106196213-10619621**	chr4	106196213	106196213	1	0	100	R1516	11	TET2
**chr4:106196252-10619625**	chr4	106196252	106196254	3	0	100	Q1529	11	TET2
**chr4:106196282-10619628**	chr4	106196282	106196282	1	0	100	Q1539	11	TET2
**chr4:106196309-10619630**	chr4	106196309	106196309	1	0	100	Q1548	11	TET2
**chr4:106196423-10619642**	chr4	106196423	106196425	3	0	100	S1586	11	TET2
**chr4:106196461-10619646**	chr4	106196461	106196461	0	1	0	Y1598	11	TET2
**chr4:106196483-10619648**	chr4	106196483	106196483	0	1	0	G1606	11	TET2
**chr4:106196521-10619652**	chr4	106196521	106196521	0	1	0	Y1618	11	TET2
**chr4:106196537-10619653**	chr4	106196537	106196537	0	1	0	Q1624	11	TET2
**chr4:106196589-10619658**	chr4	106196589	106196589	0	1	0	N1641	11	TET2
**chr4:106196644-10619664**	chr4	106196644	106196644	1	0	100	Y1659	11	TET2
**chr4:106196726-10619672**	chr4	106196726	106196728	3	0	100	Q1687	11	TET2
**chr4:106196771-10619677**	chr4	106196771	106196771	1	0	100	Q1702	11	TET2
**chr4:106196819-10619681**	chr4	106196819	106196819	1	0	100	V1718	11	TET2
**chr4:106196819-10619682**	chr4	106196819	106196820	2	0	100	V1718	11	TET2
**chr4:106196829-10619682**	chr4	106196829	106196829	1	0	100	L1721	11	TET2
**chr4:106196951-10619695**	chr4	106196951	106196951	1	0	100	I1762	11	TET2
**chr4:106197000-10619700**	chr4	106197000	106197000	1	0	100	H1778	11	TET2
**chr4:106197123-10619712**	chr4	106197123	106197123	1	0	100	L1819	11	TET2
**chr4:106197149-10619714**	chr4	106197149	106197149	0	1	0	Q1828	11	TET2
**chr4:106197248-10619724**	chr4	106197248	106197248	1	0	100	G1861	11	TET2
**chr4:106197269-10619726**	chr4	106197269	106197269	1	0	100	H1868	11	TET2
**chr4:106197273-10619727**	chr4	106197273	106197273	1	0	100	G1869	11	TET2
**chr4:106197276-10619727**	chr4	106197276	106197276	1	0	100	S1870	11	TET2
**chr4:106197285-10619728**	chr4	106197285	106197285	1	0	100	I1873	11	TET2
**chr4:106197309-10619730**	chr4	106197309	106197309	1	0	100	H1881	11	TET2
**chr4:106197317-10619731**	chr4	106197317	106197317	1	0	100	T1884	11	TET2
**chr4:106197332-10619733**	chr4	106197332	106197332	1	0	100	P1889	11	TET2
**chr4:106197348-10619734**	chr4	106197348	106197348	1	0	100	P1894	11	TET2
**chr4:106197353-10619735**	chr4	106197353	106197355	3	0	100	R1896	11	TET2
**chr4:106197360-10619736**	chr4	106197360	106197360	1	0	100	S1898	11	TET2
**chr4:106197378-10619737**	chr4	106197378	106197378	1	0	100	H1904	11	TET2
**chr4:106197384-10619739**	chr4	106197384	106197390	7	0	100	M1907	11	TET2
**chr4:106197386-10619738**	chr4	106197386	106197388	3	0	100	M1907	11	TET2
**chr4:106197552-10619755**	chr4	106197552	106197552	1	0	100	P1962	11	TET2
**chr4:106197563-10619756**	chr4	106197563	106197563	1	0	100	R1966	11	TET2
**chr5:170837540-17083754**	chr5	170837540	170837546	7	0	100	L287	11	NPM1
**chr5:170837544-17083754**	chr5	170837544	170837544	1	0	100	L287	11	NPM1
**chr5:170837548-17083754**	chr5	170837548	170837548	1	0	100	W288	11	NPM1
**chr5:170837549-17083754**	chr5	170837549	170837549	1	0	100	Q289	11	NPM1
**chr5:170837552-17083755**	chr5	170837552	170837552	1	0	100	W290	11	NPM1
**chr5:170837564-17083756**	chr5	170837564	170837564	1	0	100	L294	11	NPM1
**chr7:101840249-10184025**	chr7	101840249	101840251	3	0	100	Q531	15	CUX1
**chr7:101842129-10184213**	chr7	101842129	101842131	3	0	100	R659	16	CUX1
**chr7:101843365-10184336**	chr7	101843365	101843367	3	0	100	R670	17	CUX1

**chr7:101847749-10184775**	chr7	101847749	101847751	3	0	100	R1007	19	CUX1
**chr7:101870682-10187068**	chr7	101870682	101870684	3	0	100	A1067	21	CUX1
**chr7:101882758-10188276**	chr7	101882758	101882760	3	0	100	R1272	23	CUX1
**chr7:101921247-10192124**	chr7	101921247	101921249	3	0	100	Q531	15	CUX1
**chr7:140449171-14044917**	chr7	140449171	140449171	1	0	100	Q636	16	BRAF
**chr7:140449194-14044919**	chr7	140449194	140449194	1	0	100	D629	16	BRAF
**chr7:140453134-14045313**	chr7	140453134	140453134	1	0	100	K601	15	BRAF
**chr7:140453135-14045313**	chr7	140453135	140453136	2	0	100	V600	15	BRAF
**chr7:140453136-14045313**	chr7	140453136	140453137	2	0	100	T599	15	BRAF
**chr7:140453139-14045313**	chr7	140453139	140453139	1	0	100	T599	15	BRAF
**chr7:140453145-14045314**	chr7	140453145	140453145	1	0	100	L597	15	BRAF
**chr7:140453147-14045314**	chr7	140453147	140453149	3	0	100	G596	15	BRAF
**chr7:140453150-14045315**	chr7	140453150	140453150	1	0	100	F595	15	BRAF
**chr7:140453154-14045315**	chr7	140453154	140453154	1	0	100	D594	15	BRAF
**chr7:140453193-14045319**	chr7	140453193	140453193	1	0	100	N581	15	BRAF
**chr7:140476733-14047673**	chr7	140476733	140476733	1	0	100	R558	13	BRAF
**chr7:140476782-14047678**	chr7	140476782	140476782	1	0	100	H542	13	BRAF
**chr7:140476791-14047679**	chr7	140476791	140476791	1	0	100	H539	13	BRAF
**chr7:140476814-14047681**	chr7	140476814	140476814	1	0	100	W531	13	BRAF
**chr7:140476821-14047682**	chr7	140476821	140476821	1	0	100	T529	13	BRAF
**chr7:140477794-14047779**	chr7	140477794	140477794	1	0	100	L505	12	BRAF
**chr7:140477807-14047780**	chr7	140477807	140477807	1	0	100	E501	12	BRAF
**chr7:140477837-14047785**	chr7	140477837	140477851	15	0	100	N486	12	BRAF
**chr7:140477854-14047785**	chr7	140477854	140477854	1	0	100	L485	12	BRAF
**chr7:140477861-14047786**	chr7	140477861	140477861	1	0	100	K483	12	BRAF
**chr7:140481393-14048139**	chr7	140481393	140481393	1	0	100	Y472	11	BRAF
**chr7:140481397-14048139**	chr7	140481397	140481397	1	0	100	V471	11	BRAF
**chr7:140481402-14048140**	chr7	140481402	140481402	1	0	100	G469	11	BRAF
**chr7:140481403-14048140**	chr7	140481403	140481403	1	0	100	G469	11	BRAF
**chr7:140481408-14048140**	chr7	140481408	140481408	1	0	100	S467	11	BRAF
**chr7:140481411-14048141**	chr7	140481411	140481411	1	0	100	G466	11	BRAF
**chr7:140481417-14048141**	chr7	140481417	140481417	1	0	100	G464	11	BRAF
**chr7:140482866-14048286**	chr7	140482866	140482866	1	0	100	Q423	10	BRAF
**chr7:140482879-14048287**	chr7	140482879	140482879	1	0	100	S419	10	BRAF
**chr7:140482918-14048291**	chr7	140482918	140482918	1	0	100	L406	10	BRAF
**chr7:140482927-14048292**	chr7	140482927	140482927	1	0	100	P403	10	BRAF
**chr7:140487360-14048736**	chr7	140487360	140487360	1	0	100	R389	9	BRAF
**chr7:140487368-14048736**	chr7	140487368	140487368	1	0	100	Q386	9	BRAF
**chr7:140500165-14050016**	chr7	140500165	140500165	1	0	100	I326	7	BRAF
**chr7:140500213-14050021**	chr7	140500213	140500213	1	0	100	T310	7	BRAF
**chr7:140500228-14050022**	chr7	140500228	140500228	1	0	100	A305	7	BRAF
**chr7:140500232-14050023**	chr7	140500232	140500232	1	0	100	E304	7	BRAF
**chr7:140500260-14050026**	chr7	140500260	140500260	1	0	100	F294	7	BRAF
**chr7:140501260-14050126**	chr7	140501260	140501260	1	0	100	R271	6	BRAF
**chr7:140501299-14050129**	chr7	140501299	140501299	1	0	100	G258	6	BRAF
**chr7:140501350-14050135**	chr7	140501350	140501350	1	0	100	T241	6	BRAF
**chr7:140534557-14053455**	chr7	140534557	140534557	1	0	100	T119	3	BRAF

**chr7:140534608-14053460**	chr7	140534608	140534608	1	0	100	S102	3	BRAF
**chr7:140549937-14054993**	chr7	140549937	140549937	1	0	100	H72	2	BRAF
**chr7:140549946-14054994**	chr7	140549946	140549946	1	0	100	G69	2	BRAF
**chr7:140549972-14054997**	chr7	140549972	140549972	1	0	100	H60	2	BRAF
**chr7:140624426-14062442**	chr7	140624426	140624426	0	1	0	E26	1	BRAF
**chr7:140624467-14062446**	chr7	140624467	140624467	0	1	0	E13	1	BRAF
**chr7:140624482-14062448**	chr7	140624482	140624482	0	1	0	G8	1	BRAF
**chr7:148504761-14850476**	chr7	148504761	148504761	1	0	100	E745	20	EZH2
**chr7:148504786-14850478**	chr7	148504786	148504788	3	0	100	A736	20	EZH2
**chr7:148506162-14850616**	chr7	148506162	148506162	1	0	100	Non-coding		EZH2
**chr7:148506167-14850616**	chr7	148506167	148506167	1	0	100	Y731	19	EZH2
**chr7:148506185-14850618**	chr7	148506185	148506185	1	0	100	E725	19	EZH2
**chr7:148506428-14850642**	chr7	148506428	148506428	1	0	100	S695	18	EZH2
**chr7:148506433-14850643**	chr7	148506433	148506433	1	0	100	N693	18	EZH2
**chr7:148506436-14850643**	chr7	148506436	148506438	3	0	100	A692	18	EZH2
**chr7:148506443-14850644**	chr7	148506443	148506443	1	0	100	R690	18	EZH2
**chr7:148506461-14850646**	chr7	148506461	148506461	1	0	100	R684	18	EZH2
**chr7:148506467-14850646**	chr7	148506467	148506467	1	0	100	A682	18	EZH2
**chr7:148506470-14850648**	chr7	148506470	148506482	13	0	100	D681	18	EZH2
**chr7:148506477-14850647**	chr7	148506477	148506477	1	0	100	V679	18	EZH2
**chr7:148507429-14850742**	chr7	148507429	148507429	1	0	100	N675	17	EZH2
**chr7:148507454-14850745**	chr7	148507454	148507454	1	0	100	M667	17	EZH2
**chr7:148507463-14850746**	chr7	148507463	148507463	1	0	100	D664	17	EZH2
**chr7:148507476-14850747**	chr7	148507476	148507476	1	0	100	G660	17	EZH2
**chr7:148507487-14850748**	chr7	148507487	148507487	1	0	100	A656	17	EZH2
**chr7:148507497-14850749**	chr7	148507497	148507497	1	0	100	Q653	17	EZH2
**chr7:148508727-14850872**	chr7	148508727	148508727	1	0	100	Y646	16	EZH2
**chr7:148508728-14850872**	chr7	148508728	148508728	1	0	100	Y646	16	EZH2
**chr7:148508733-14850873**	chr7	148508733	148508733	1	0	100	S644	16	EZH2
**chr7:148508788-14850878**	chr7	148508788	148508788	1	0	100	V626	16	EZH2
**chr7:148511153-14851115**	chr7	148511153	148511155	3	0	100	R583	15	EZH2
**chr7:148511172-14851117**	chr7	148511172	148511172	1	0	100	P577	15	EZH2
**chr7:148511184-14851118**	chr7	148511184	148511184	1	0	100	T573	15	EZH2
**chr7:148512029-14851202**	chr7	148512029	148512029	1	0	100	K550	14	EZH2
**chr7:148512036-14851203**	chr7	148512036	148512036	1	0	100	C548	14	EZH2
**chr7:148512065-14851206**	chr7	148512065	148512065	1	0	100	S538	14	EZH2
**chr7:148512600-14851260**	chr7	148512600	148512600	1	0	100	K515	13	EZH2
**chr7:148513776-14851377**	chr7	148513776	148513776	1	0	100	R502	12	EZH2
**chr7:148514325-14851432**	chr7	148514325	148514325	1	0	100	T467	11	EZH2
**chr7:148514396-14851439**	chr7	148514396	148514396	1	0	100	S443	11	EZH2
**chr7:148514466-14851446**	chr7	148514466	148514466	1	0	100	Q420	11	EZH2
**chr7:148515023-14851502**	chr7	148515023	148515023	1	0	100	E396	11	EZH2
**chr7:148515127-14851512**	chr7	148515127	148515127	1	0	100	R361	10	EZH2
**chr7:148516722-14851672**	chr7	148516722	148516722	1	0	100	N322	9	EZH2
**chr7:148523590-14852359**	chr7	148523590	148523590	1	0	100	R288	8	EZH2
**chr7:148523591-14852359**	chr7	148523591	148523591	1	0	100	A255	8	EZH2
**chr7:148523690-14852369**	chr7	148523690	148523690	1	0	100	A255	8	EZH2

**chr7:148523708-14852370**	chr7	148523708	148523708	1	0	100	E249	8	EZH2
**chr7:148523717-14852371**	chr7	148523717	148523717	1	0	100	E246	8	EZH2
**chr7:148524336-14852433**	chr7	148524336	148524338	3	0	100	R216	7	EZH2
**chr7:148524338-14852433**	chr7	148524338	148524338	1	0	100	R216	7	EZH2
**chr7:148524347-14852434**	chr7	148524347	148524347	1	0	100	R213	7	EZH2
**chr7:148525836-14852583**	chr7	148525836	148525838	3	0	100	R207	6	EZH2
**chr7:148525904-14852590**	chr7	148525904	148525904	1	0	100	D185	6	EZH2
**chr7:148526829-14852682**	chr7	148526829	148526829	1	0	100	G159	5	EZH2
**chr7:148526870-14852687**	chr7	148526870	148526870	1	0	100	F145	5	EZH2
**chr7:148526901-14852690**	chr7	148526901	148526901	1	0	100	G135	5	EZH2
**chr7:148526905-14852690**	chr7	148526905	148526907	3	0	100	Y133	5	EZH2
**chr7:148529781-14852978**	chr7	148529781	148529781	1	0	100	A103	4	EZH2
**chr7:148529784-14852978**	chr7	148529784	148529784	1	0	100	N102	4	EZH2
**chr7:148543575-14854357**	chr7	148543575	148543575	1	0	100	R78	3	EZH2
**chr7:148543621-14854362**	chr7	148543621	148543621	1	0	100	R63	3	EZH2
**chr7:148543641-14854365**	chr7	148543641	148543653	13	0	100	L56	3	EZH2
**chr7:148543643-14854364**	chr7	148543643	148543643	1	0	100	I55	3	EZH2
**chr7:148544317-14854431**	chr7	148544317	148544317	1	0	100	R25	2	EZH2
**chr7:148544345-14854434**	chr7	148544345	148544345	1	0	100	R16	2	EZH2
**chr7:148544353-14854435**	chr7	148544353	148544353	1	0	100	V13	2	EZH2
**chr8:117864189-11786418**	chr8	117864189	117864189	1	0	100	P490	11	RAD21
**chr8:117864195-11786420**	chr8	117864195	117864200	6	0	100	V488	11	RAD21
**chr8:117864201-11786420**	chr8	117864201	117864201	1	0	100	E486	11	RAD21
**chr8:117864206-11786420**	chr8	117864206	117864206	1	0	100	D484	11	RAD21
**chr8:117864799-11786479**	chr8	117864799	117864799	1	0	100	R437	10	RAD21
**chr8:117864815-11786481**	chr8	117864815	117864815	1	0	100	Q432	10	RAD21
**chr8:117864824-11786482**	chr8	117864824	117864824	1	0	100	D429	10	RAD21
**chr8:117864871-11786487**	chr8	117864871	117864871	1	0	100	L413	10	RAD21
**chr8:117864881-11786488**	chr8	117864881	117864881	1	0	100	A410	10	RAD21
**chr8:117864931-11786493**	chr8	117864931	117864931	1	0	100	L393	10	RAD21
**chr8:117868887-11786888**	chr8	117868887	117868887	1	0	100	S271	7	RAD21
**chr8:117868912-11786891**	chr8	117868912	117868912	1	0	100	D263	7	RAD21
**chr8:117868915-11786891**	chr8	117868915	117868915	1	0	100	D262	7	RAD21
**chr8:117868983-11786898**	chr8	117868983	117868983	1	0	100	G239	7	RAD21
**chr8:117869671-11786967**	chr8	117869671	117869671	1	0	100	S175	6	RAD21
**chr8:117869692-11786969**	chr8	117869692	117869692	1	0	100	R168	6	RAD21
**chr8:117869704-11786970**	chr8	117869704	117869704	1	0	100	G164	6	RAD21
**chr8:128750512-12875051**	chr8	128750512	128750512	0	1	0	P17	2	MYC
**chr8:128750525-12875052**	chr8	128750525	128750525	1	0	100	S21	2	MYC
**chr8:128750529-12875052**	chr8	128750529	128750529	1	0	100	F22	2	MYC
**chr8:128750534-12875053**	chr8	128750534	128750534	1	0	100	N24	2	MYC
**chr8:128750543-12875054**	chr8	128750543	128750543	1	0	100	Y27	2	MYC
**chr8:128750642-12875064**	chr8	128750642	128750642	1	0	100	A59	2	MYC
**chr8:128750680-12875068**	chr8	128750680	128750680	1	0	100	T73	2	MYC
**chr8:128750684-12875068**	chr8	128750684	128750684	1	0	100	P74	2	MYC
**chr8:128750687-12875068**	chr8	128750687	128750687	1	0	100	P75	2	MYC
**chr8:128750921-12875092**	chr8	128750921	128750921	1	0	100	F153	2	MYC

**chr8:128750945-12875094**	chr8	128750945	128750945	1	0	100	S161	2	MYC
**chr8:128750953-12875095**	chr8	128750953	128750953	1	0	100	L164	2	MYC
**chr8:128751046-12875104**	chr8	128751046	128751046	1	0	100	A180	2	MYC
**chr8:128751185-12875118**	chr8	128751185	128751185	1	0	100	S241	2	MYC
**chr8:128751254-12875125**	chr8	128751254	128751254	1	0	100	S264	2	MYC
**chr8:128752656-12875265**	chr8	128752656	128752656	1	0	100	D273	3	MYC
**chr8:128752800-12875280**	chr8	128752800	128752800	1	0	100	Q231	3	MYC
**chr8:128752803-12875280**	chr8	128752803	128752803	1	0	100	H322	3	MYC
**chr8:128752882-12875288**	chr8	128752882	128752882	1	0	100	L348	3	MYC
**chr9:5070022-5070027**	chr9	5070022	5070027	6	0	100	F537	11	JAK2
**chr9:5070033-5070038**	chr9	5070033	5070038	6	0	100	R541	11	JAK2
**chr9:5070034-5070039**	chr9	5070034	5070039	6	0	100	N542	11	JAK2
**chr9:5070036-5070041**	chr9	5070036	5070041	6	0	100	E543	11	JAK2
**chr9:5073753-5073753**	chr9	5073753	5073753	1	0	100	L611	13	JAK2
**chr9:5073770-5073770**	chr9	5073770	5073770	1	0	100	V617	13	JAK2
**chr9:5073773-5073773**	chr9	5073773	5073773	1	0	100	C618	13	JAK2
**chr9:5078360-5078362**	chr9	5078360	5078362	3	0	100	R683	15	JAK2
**chr9:139390682-13939068**	chr9	139390682	139390684	0	3	0	Q2503	34	NOTCH1
**chr9:139390715-13939071**	chr9	139390715	139390717	3	0	100	S2492	34	NOTCH1
**chr9:139390730-13939073**	chr9	139390730	139390732	3	0	100	Q2487	34	NOTCH1
**chr9:139390790-13939079**	chr9	139390790	139390792	3	0	100	S2467	34	NOTCH1
**chr9:139390814-13939081**	chr9	139390814	139390816	3	0	100	Q2459	34	NOTCH1
**chr9:139390859-13939086**	chr9	139390859	139390861	3	0	100	Q2444	34	NOTCH1
**chr9:139390871-13939087**	chr9	139390871	139390873	3	0	100	Q2440	34	NOTCH1
**chr9:139391018-13939102**	chr9	139391018	139391020	3	0	100	Q2391	34	NOTCH1
**chr9:139397767-13939776**	chr9	139397767	139397769	3	0	100	L1678	27	NOTCH1
**chr9:139399295-13939929**	chr9	139399295	139399297	3	0	100	I1616	26	NOTCH1
**chr9:139399343-13939934**	chr9	139399343	139399345	3	0	100	L1600	26	NOTCH1
**chr9:139399349-13939935**	chr9	139399349	139399351	3	0	100	R1598	26	NOTCH1
**chr9:139399364-13939936**	chr9	139399364	139399366	3	0	100	L1593	26	NOTCH1
**chr9:139399388-13939939**	chr9	139399388	139399390	3	0	100	L1585	26	NOTCH1
**chr9:139399421-13939942**	chr9	139399421	139399423	3	0	100	L1574	26	NOTCH1
**chr10:27389295-27389423**	chr10	27389295	27389423	129	0	100	5'UTR		ANKRD26
**chr10:89624296-89624298**	chr10	89624296	89624298	3	0	100	D24	1	PTEN
**chr10:89692794-89692794**	chr10	89692794	89692794	1	0	100	H93	5	PTEN
**chr10:89692901-89692901**	chr10	89692901	89692901	1	0	100	G129	5	PTEN
**chr10:89692904-89692904**	chr10	89692904	89692904	1	0	100	R130	5	PTEN
**chr10:89692911-89692911**	chr10	89692911	89692911	1	0	100	G132	5	PTEN
**chr10:89692922-89692922**	chr10	89692922	89692922	1	0	100	C136	5	PTEN
**chr10:89692980-89692980**	chr10	89692980	89692980	1	0	100	Y155	5	PTEN
**chr10:89717609-89717609**	chr10	89717609	89717609	1	0	100	Non-coding		PTEN
**chr10:89717615-89717615**	chr10	89717615	89717615	1	0	100	Q214	7	PTEN
**chr10:89717661-89717661**	chr10	89717661	89717661	1	0	100	S229	7	PTEN
**chr10:89717672-89717672**	chr10	89717672	89717672	1	0	100	R233	7	PTEN
**chr10:89717699-89717699**	chr10	89717699	89717699	1	0	100	E242	7	PTEN
**chr10:89717708-89717708**	chr10	89717708	89717708	1	0	100	Q245	7	PTEN
**chr10:89717712-89717712**	chr10	89717712	89717712	1	0	100	P246	7	PTEN

**chr10:89717770-89717770**	chr10	89717770	89717770	1	0	100	K267	7	PTEN
**chr10:89720663-89720665**	chr10	89720663	89720665	3	0	100	H272	8	PTEN
**chr10:89720681-89720683**	chr10	89720681	89720683	3	0	100	F278	8	PTEN
**chr10:89720852-89720854**	chr10	89720852	89720854	3	0	100	R335	8	PTEN
**chr10:112343991-1123439**	chr10	112343991	112343991	1	0	100	R381	13	SMC3
**chr10:112344153-1123441**	chr10	112344153	112344153	1	0	100	N435	13	SMC3
**chr10:112356173-1123561**	chr10	112356173	112356173	1	0	100	R661	19	SMC3
**chr10:112356176-1123561**	chr10	112356176	112356176	1	0	100	G662	19	SMC3
**chr10:112361811-1123618**	chr10	112361811	112361811	1	0	100	S994	25	SMC3
**chr10:112361875-1123618**	chr10	112361875	112361875	1	0	100	M1015	25	SMC3
**chr10:112362979-1123629**	chr10	112362979	112362979	1	0	100	I1171	28	SMC3
**chr10:112363013-1123630**	chr10	112363013	112363013	1	0	100	A1183	28	SMC3
**chr11:32410724-32413527**	chr11	32410724	32413527	1939	865	69.15121255	Non-coding		WT1
**chr11:32413555-32413557**	chr11	32413555	32413557	0	3	0	H465	9	WT1
**chr11:32413557-32413557**	chr11	32413557	32413557	0	1	0	H465	9	WT1
**chr11:32413558-32413560**	chr11	32413558	32413560	0	3	0	D464	9	WT1
**chr11:32413560-32413560**	chr11	32413560	32413560	0	1	0	D464	9	WT1
**chr11:32413563-32413569**	chr11	32413563	32413569	0	7	0	R462	9	WT1
**chr11:32413564-32413566**	chr11	32413564	32413566	0	3	0	R462	9	WT1
**chr11:32413566-32413566**	chr11	32413566	32413566	0	1	0	R462	9	WT1
**chr11:32413576-32413578**	chr11	32413576	32413578	2	1	66.66666667	R458	9	WT1
**chr11:32413578-32413578**	chr11	32413578	32413578	1	0	100	R458	9	WT1
**chr11:32413610-32413610**	chr11	32413610	32413610	1	0	100	G447	9	WT1
**chr11:32414249-32414251**	chr11	32414249	32414251	3	0	100	R434	8	WT1
**chr11:32414250-32414250**	chr11	32414250	32414250	1	0	100	R434	8	WT1
**chr11:32414251-32414251**	chr11	32414251	32414251	1	0	100	R434	8	WT1
**chr11:32414261-32414263**	chr11	32414261	32414263	3	0	100	R430	8	WT1
**chr11:32414263-32414263**	chr11	32414263	32414263	1	0	100	R430	8	WT1
**chr11:32417827-32417827**	chr11	32417827	32417827	1	0	100	Q409	7	WT1
**chr11:32417836-32417836**	chr11	32417836	32417836	1	0	100	S406	7	WT1
**chr11:32417850-32417850**	chr11	32417850	32417850	1	0	100	R401	7	WT1
**chr11:32417853-32417853**	chr11	32417853	32417853	1	0	100	K400	7	WT1
**chr11:32417856-32417856**	chr11	32417856	32417856	1	0	100	N399	7	WT1
**chr11:32417894-32417894**	chr11	32417894	32417894	1	0	100	S386	7	WT1
**chr11:32417906-32417908**	chr11	32417906	32417908	3	0	100	A382	7	WT1
**chr11:32417909-32417911**	chr11	32417909	32417911	3	0	100	S381	7	WT1
**chr11:32417910-32417910**	chr11	32417910	32417910	1	0	100	S381	7	WT1
**chr11:32417912-32417914**	chr11	32417912	32417914	3	0	100	R380	7	WT1
**chr11:32417914-32417914**	chr11	32417914	32417914	1	0	100	R380	7	WT1
**chr11:32417942-32417944**	chr11	32417942	32417944	3	0	100	R370	7	WT1
**chr11:32417945-32417947**	chr11	32417945	32417947	3	0	100	R369	7	WT1
**chr11:32417947-32417947**	chr11	32417947	32417947	1	0	100	R369	7	WT1
**chr11:108183167-1081831**	chr11	108183167	108183167	1	0	100	N1983	40	ATM
**chr11:108183177-1081831**	chr11	108183177	108183177	1	0	100	I1986	40	ATM
**chr11:108183182-1081831**	chr11	108183182	108183182	1	0	100	S1988	40	ATM
**chr11:108183186-1081831**	chr11	108183186	108183186	1	0	100	L1989	40	ATM
**chr11:108183192-1081831**	chr11	108183192	108183192	1	0	100	E1991	40	ATM

**chr11:108183212-1081832**	chr11	108183212	108183212	1	0	100	G1998	40	ATM
**chr11:108236086-1082360**	chr11	108236086	108236086	1	0	100	R3008	63	ATM
**chr11:108236087-1082360**	chr11	108236087	108236087	1	0	100	R3008	63	ATM
**chr11:108236096-1082360**	chr11	108236096	108236096	1	0	100	M3011	63	ATM
**chr11:108236203-1082362**	chr11	108236203	108236203	1	0	100	R3047	63	ATM
**chr11:108236216-1082362**	chr11	108236216	108236216	1	0	100	G3051	63	ATM
**chr11:118343306-1183433**	chr11	118343306	118343306	1	0	100	R478	3	KMT2A
**chr11:118343381-1183433**	chr11	118343381	118343381	1	0	100	R503	3	KMT2A
**chr11:118343997-1183439**	chr11	118343997	118343997	1	0	100	H708	3	KMT2A
**chr11:118344076-1183440**	chr11	118344076	118344076	1	0	100	R734	3	KMT2A
**chr11:118344107-1183441**	chr11	118344107	118344107	1	0	100	R745	3	KMT2A
**chr11:118344186-1183441**	chr11	118344186	118344186	1	0	100	T771	3	KMT2A
**chr11:118344222-1183442**	chr11	118344222	118344223	2	0	100	S783	3	KMT2A
**chr11:118344279-1183442**	chr11	118344279	118344279	1	0	100	S802	3	KMT2A
**chr11:118344314-1183443**	chr11	118344314	118344314	0	1	0	Q814	3	KMT2A
**chr11:118344450-1183444**	chr11	118344450	118344450	1	0	100	S859	3	KMT2A
**chr11:118344458-1183444**	chr11	118344458	118344458	1	0	100	R862	3	KMT2A
**chr11:118344530-1183445**	chr11	118344530	118344530	1	0	100	R886	3	KMT2A
**chr11:118344593-1183445**	chr11	118344593	118344593	1	0	100	P907	3	KMT2A
**chr11:118352446-1183524**	chr11	118352446	118352446	1	0	100	E1217	7	KMT2A
**chr11:118352528-1183525**	chr11	118352528	118352528	1	0	100	T1245	7	KMT2A
**chr11:118352550-1183525**	chr11	118352550	118352550	1	0	100	P1252	7	KMT2A
**chr11:118354989-1183549**	chr11	118354989	118354989	1	0	100	I1393	9	KMT2A
**chr11:118354997-1183549**	chr11	118354997	118354997	1	0	100	D1396	9	KMT2A
**chr11:118355592-1183555**	chr11	118355592	118355592	1	0	100	E1412	10	KMT2A
**chr11:118355604-1183556**	chr11	118355604	118355604	1	0	100	E1416	10	KMT2A
**chr11:118355626-1183556**	chr11	118355626	118355626	1	0	100	L1423	10	KMT2A
**chr11:118355640-1183556**	chr11	118355640	118355640	1	0	100	I1428	10	KMT2A
**chr11:118366578-1183665**	chr11	118366578	118366580	3	0	100	P1843	19	KMT2A
**chr11:118366587-1183665**	chr11	118366587	118366589	3	0	100	P1846	19	KMT2A
**chr11:118373709-1183737**	chr11	118373709	118373709	1	0	100	L2368	27	KMT2A
**chr11:118373727-1183737**	chr11	118373727	118373727	1	0	100	H2374	27	KMT2A
**chr11:118373751-1183737**	chr11	118373751	118373751	1	0	100	R2382	27	KMT2A
**chr11:118373919-1183739**	chr11	118373919	118373919	1	0	100	E2438	27	KMT2A
**chr11:118373928-1183739**	chr11	118373928	118373928	0	1	0	K2441	27	KMT2A
**chr11:118374172-1183741**	chr11	118374172	118374172	1	0	100	T2522	27	KMT2A
**chr11:118374197-1183741**	chr11	118374197	118374197	1	0	100	L2530	27	KMT2A
**chr11:118374411-1183744**	chr11	118374411	118374411	1	0	100	M2602	27	KMT2A
**chr11:118374769-1183747**	chr11	118374769	118374769	1	0	100	D2721	27	KMT2A
**chr11:118374913-1183749**	chr11	118374913	118374913	1	0	100	H2769	27	KMT2A
**chr11:118375065-1183750**	chr11	118375065	118375065	1	0	100	D2820	27	KMT2A
**chr11:118375411-1183754**	chr11	118375411	118375411	1	0	100	S2935	27	KMT2A
**chr11:118375474-1183754**	chr11	118375474	118375474	1	0	100	S2956	27	KMT2A
**chr11:118375621-1183756**	chr11	118375621	118375621	1	0	100	D3005	27	KMT2A
**chr11:118375666-1183756**	chr11	118375666	118375666	1	0	100	G3020	27	KMT2A
**chr11:118375798-1183757**	chr11	118375798	118375798	1	0	100	N3064	27	KMT2A
**chr11:118375867-1183758**	chr11	118375867	118375867	1	0	100	N3087	27	KMT2A

**chr11:118376290-1183762**	chr11	118376290	118376290	1	0	100	R3228	27	KMT2A
**chr11:118376322-1183763**	chr11	118376322	118376322	1	0	100	P3239	27	KMT2A
**chr11:118376934-1183769**	chr11	118376934	118376936	3	0	100	A3443	27	KMT2A
**chr11:118376991-1183769**	chr11	118376991	118376991	1	0	100	L3462	27	KMT2A
**chr11:118377127-1183771**	chr11	118377127	118377127	1	0	100	A3507	27	KMT2A
**chr11:118377208-1183772**	chr11	118377208	118377208	1	0	100	P3534	27	KMT2A
**chr11:119148467-1191484**	chr11	119148467	119148467	1	0	100	Non-coding		CBL
**chr11:119148486-1191484**	chr11	119148486	119148486	1	0	100	R343	7	CBL
**chr11:119148513-1191485**	chr11	119148513	119148513	1	0	100	L352	7	CBL
**chr11:119148875-1191488**	chr11	119148875	119148875	1	0	100	Non-coding		CBL
**chr11:119148891-1191488**	chr11	119148891	119148891	1	0	100	Y371	8	CBL
**chr11:119148919-1191489**	chr11	119148919	119148919	1	0	100	L380	8	CBL
**chr11:119148922-1191489**	chr11	119148922	119148922	1	0	100	C381	8	CBL
**chr11:119148930-1191489**	chr11	119148930	119148930	1	0	100	C384	8	CBL
**chr11:119148966-1191489**	chr11	119148966	119148966	1	0	100	C396	8	CBL
**chr11:119148973-1191489**	chr11	119148973	119148973	1	0	100	H398	8	CBL
**chr11:119148981-1191489**	chr11	119148981	119148983	3	0	100	C401	8	CBL
**chr11:119148991-1191489**	chr11	119148991	119148991	1	0	100	C404	8	CBL
**chr11:119149002-1191490**	chr11	119149002	119149004	3	0	100	W408	8	CBL
**chr11:119149218-1191492**	chr11	119149218	119149218	1	0	100	Non-coding		CBL
**chr11:119149238-1191492**	chr11	119149238	119149238	1	0	100	C416	9	CBL
**chr11:119149242-1191492**	chr11	119149242	119149242	1	0	100	P417	9	CBL
**chr11:119149245-1191492**	chr11	119149245	119149245	1	0	100	F418	9	CBL
**chr11:119149247-1191492**	chr11	119149247	119149249	3	0	100	C419	9	CBL
**chr11:119149251-1191492**	chr11	119149251	119149251	1	0	100	R420	9	CBL
**chr11:119149290-1191492**	chr11	119149290	119149290	1	0	100	P433	9	CBL
**chr12:11803080-11803080**	chr12	11803080	11803080	1	0	100	Q7	1	ETV6
**chr12:11803084-11803084**	chr12	11803084	11803084	1	0	100	C8	1	ETV6
**chr12:11905391-11905391**	chr12	11905391	11905391	1	0	100	R14	2	ETV6
**chr12:11905397-11905397**	chr12	11905397	11905397	1	0	100	S16	2	ETV6
**chr12:11905406-11905406**	chr12	11905406	11905406	1	0	100	P19	2	ETV6
**chr12:11905424-11905424**	chr12	11905424	11905424	1	0	100	P25	2	ETV6
**chr12:11905465-11905465**	chr12	11905465	11905465	1	0	100	R39	2	ETV6
**chr12:11905513-11905513**	chr12	11905513	11905513	1	0	100	R55	2	ETV6
**chr12:11992136-11992136**	chr12	11992136	11992136	1	0	100	E76	3	ETV6
**chr12:11992143-11992143**	chr12	11992143	11992143	1	0	100	S78	3	ETV6
**chr12:11992208-11992208**	chr12	11992208	11992208	1	0	100	E100	3	ETV6
**chr12:11992217-11992217**	chr12	11992217	11992217	1	0	100	R103	3	ETV6
**chr12:11992223-11992223**	chr12	11992223	11992223	1	0	100	R105	3	ETV6
**chr12:12006436-12006436**	chr12	12006436	12006436	1	0	100	H135	4	ETV6
**chr12:12006464-12006464**	chr12	12006464	12006464	1	0	100	E145	4	ETV6
**chr12:12022361-12022361**	chr12	12022361	12022361	1	0	100	N156	5	ETV6
**chr12:12022435-12022435**	chr12	12022435	12022435	0	1	0	R181	5	ETV6
**chr12:12022498-12022498**	chr12	12022498	12022498	1	0	100	R202	5	ETV6
**chr12:12022504-12022504**	chr12	12022504	12022504	1	0	100	P204	5	ETV6
**chr12:12022522-12022522**	chr12	12022522	12022522	1	0	100	R210	5	ETV6
**chr12:12022535-12022535**	chr12	12022535	12022535	1	0	100	P214	5	ETV6

**chr12:12022544-12022544**	chr12	12022544	12022544	1	0	100	R217	5	ETV6
**chr12:12022560-12022560**	chr12	12022560	12022560	1	0	100	R222	5	ETV6
**chr12:12022577-12022577**	chr12	12022577	12022577	1	0	100	N228	5	ETV6
**chr12:12022644-12022644**	chr12	12022644	12022644	1	0	100	E250	5	ETV6
**chr12:12022670-12022670**	chr12	12022670	12022670	1	0	100	R259	5	ETV6
**chr12:12022672-12022672**	chr12	12022672	12022672	1	0	100	Q260	5	ETV6
**chr12:12022819-12022819**	chr12	12022819	12022819	1	0	100	R309	5	ETV6
**chr12:12022825-12022825**	chr12	12022825	12022825	1	0	100	D311	5	ETV6
**chr12:12022849-12022849**	chr12	12022849	12022849	1	0	100	M319	5	ETV6
**chr12:12022865-12022865**	chr12	12022865	12022865	1	0	100	P324	5	ETV6
**chr12:12022873-12022873**	chr12	12022873	12022873	1	0	100	E327	5	ETV6
**chr12:12037423-12037423**	chr12	12037423	12037423	1	0	100	S352	6	ETV6
**chr12:12037445-12037445**	chr12	12037445	12037445	1	0	100	R359	6	ETV6
**chr12:12037474-12037474**	chr12	12037474	12037474	1	0	100	R369	6	ETV6
**chr12:12037501-12037501**	chr12	12037501	12037501	1	0	100	R378	6	ETV6
**chr12:12037511-12037511**	chr12	12037511	12037511	1	0	100	G381	6	ETV6
**chr12:12037517-12037517**	chr12	12037517	12037517	1	0	100	H383	6	ETV6
**chr12:12038880-12038880**	chr12	12038880	12038880	1	0	100	Y391	7	ETV6
**chr12:12038893-12038893**	chr12	12038893	12038893	1	0	100	R396	7	ETV6
**chr12:12038903-12038903**	chr12	12038903	12038903	1	0	100	R399	7	ETV6
**chr12:12038909-12038909**	chr12	12038909	12038909	1	0	100	Y401	7	ETV6
**chr12:12038912-12038912**	chr12	12038912	12038912	1	0	100	Y402	7	ETV6
**chr12:12038914-12038914**	chr12	12038914	12038914	1	0	100	K403	7	ETV6
**chr12:12038932-12038932**	chr12	12038932	12038932	1	0	100	K409	7	ETV6
**chr12:12038950-12038950**	chr12	12038950	12038950	1	0	100	L415	7	ETV6
**chr12:12043910-12043910**	chr12	12043910	12043910	1	0	100	R430	8	ETV6
**chr12:12043919-12043919**	chr12	12043919	12043919	1	0	100	R433	8	ETV6
**chr12:25362837-25362839**	chr12	25362837	25362839	3	0	100	D153	5	KRAS
**chr12:25378560-25378562**	chr12	25378560	25378562	3	0	100	A146	4	KRAS
**chr12:25378571-25378571**	chr12	25378571	25378571	1	0	100	E143	4	KRAS
**chr12:25378580-25378580**	chr12	25378580	25378580	1	0	100	P140	4	KRAS
**chr12:25378585-25378585**	chr12	25378585	25378585	1	0	100	G138	4	KRAS
**chr12:25378591-25378591**	chr12	25378591	25378591	1	0	100	S136	4	KRAS
**chr12:25378609-25378609**	chr12	25378609	25378609	1	0	100	A130	4	KRAS
**chr12:25378641-25378643**	chr12	25378641	25378643	3	0	100	D119	4	KRAS
**chr12:25378647-25378647**	chr12	25378647	25378647	1	0	100	K117	4	KRAS
**chr12:25378651-25378651**	chr12	25378651	25378651	1	0	100	N116	4	KRAS
**chr12:25378676-25378676**	chr12	25378676	25378676	1	0	100	D108	4	KRAS
**chr12:25378706-25378706**	chr12	25378706	25378706	1	0	100	E98	4	KRAS
**chr12:25380266-25380268**	chr12	25380266	25380268	3	0	100	Y64	3	KRAS
**chr12:25380271-25380271**	chr12	25380271	25380271	1	0	100	E63	3	KRAS
**chr12:25380275-25380275**	chr12	25380275	25380275	1	0	100	Q61	3	KRAS
**chr12:25380276-25380276**	chr12	25380276	25380276	1	0	100	Q61	3	KRAS
**chr12:25380279-25380279**	chr12	25380279	25380279	1	0	100	G60	3	KRAS
**chr12:25380283-25380283**	chr12	25380283	25380283	1	0	100	A59	3	KRAS
**chr12:25380285-25380285**	chr12	25380285	25380285	1	0	100	T58	3	KRAS
**chr12:25398217-25398219**	chr12	25398217	25398219	3	0	100	R34	2	KRAS

**chr12:25398244-25398246**	chr12	25398244	25398246	3	0	100	Q25	2	KRAS	
**chr12:25398255-25398255**	chr12	25398255	25398255	1	0	100	Q22	2	KRAS	
**chr12:25398262-25398262**	chr12	25398262	25398262	1	0	100	L19	2	KRAS	
**chr12:25398265-25398267**	chr12	25398265	25398267	3	0	100	A18	2	KRAS	
**chr12:25398279-25398279**	chr12	25398279	25398279	1	0	100	V14	2	KRAS	
**chr12:25398281-25398281**	chr12	25398281	25398281	1	0	100	G13	2	KRAS	
**chr12:25398282-25398282**	chr12	25398282	25398282	1	0	100	G13	2	KRAS	
**chr12:25398284-25398284**	chr12	25398284	25398284	1	0	100	G13	2	KRAS	
**chr12:25398285-25398285**	chr12	25398285	25398285	1	0	100	G12	2	KRAS	
**chr12:112888156-1128881**	chr12	112888156	112888156	1	0	100	N58	3	PTPN11	
**chr12:112888163-1128881**	chr12	112888163	112888163	1	0	100	G60	3	PTPN11	
**chr12:112888165-1128881**	chr12	112888165	112888165	1	0	100	D61	3	PTPN11	
**chr12:112888189-1128881**	chr12	112888189	112888189	1	0	100	E69	3	PTPN11	
**chr12:112888195-1128881**	chr12	112888195	112888195	1	0	100	F71	3	PTPN11	
**chr12:112888199-1128881**	chr12	112888199	112888199	1	0	100	A72	3	PTPN11	
**chr12:112888202-1128882**	chr12	112888202	112888202	1	0	100	T73	3	PTPN11	
**chr12:112888210-1128882**	chr12	112888210	112888210	1	0	100	E76	3	PTPN11	
**chr12:112910785-1129107**	chr12	112910785	112910785	1	0	100	R265	7	PTPN11	
**chr12:112910827-1129108**	chr12	112910827	112910827	1	0	100	Y279	7	PTPN11	
**chr12:112910837-1129108**	chr12	112910837	112910837	1	0	100	I282	7	PTPN11	
**chr12:112926248-1129262**	chr12	112926248	112926248	1	0	100	A461	12	PTPN11	
**chr12:112926249-1129262**	chr12	112926249	112926249	1	0	100	A461	12	PTPN11	
**chr12:112926270-1129262**	chr12	112926270	112926270	1	0	100	T468	12	PTPN11	
**chr12:112926851-1129268**	chr12	112926851	112926851	1	0	100	P491	13	PTPN11	
**chr12:112926852-1129268**	chr12	112926852	112926852	1	0	100	P491	13	PTPN11	
**chr12:112926872-1129268**	chr12	112926872	112926872	1	0	100	R498	13	PTPN11	
**chr12:112926885-1129268**	chr12	112926885	112926885	1	0	100	S502	13	PTPN11	
**chr12:112926888-1129268**	chr12	112926888	112926888	1	0	100	G503	13	PTPN11	
**chr12:112926898-1129268**	chr12	112926898	112926898	1	0	100	Q510	13	PTPN11	
**chr12:112926900-1129269**	chr12	112926900	112926900	1	0	100	T511	13	PTPN11	
**chr12:112926915-1129269**	chr12	112926915	112926915	1	0	100	R512	13	PTPN11	
**chr13:28592614-28592626**	chr13	28592614	28592626	13	0	100	S840-V843	20	FLT3	
**chr13:28592623-28592623**	chr13	28592623	28592623	1	0	100	N841	20	FLT3	
**chr13:28592629-28592629**	chr13	28592629	28592629	1	0	100	D839	20	FLT3	
**chr13:28592635-28592637**	chr13	28592635	28592637	3	0	100	M837	20	FLT3	
**chr13:28592642-28592642**	chr13	28592642	28592642	1	0	100	D835	20	FLT3	
**chr13:28592644-28592644**	chr13	28592644	28592644	1	0	100	R834	20	FLT3	
**chr13:28602340-28602340**	chr13	28602340	28602340	1	0	100	N676	16	FLT3	
**chr13:28602380-28602380**	chr13	28602380	28602380	1	0	100	K663	16	FLT3	
**chr13:28608107-28608107**	chr13	28608107	28608107	0	1	0	A620	15	FLT3	
**chr13:28608218-28608218**	chr13	28608218	28608218	1	0	100	G611	14	FLT3	
**chr13:28608218-28608318**	chr13	28608218	28608318	101	0	100	Q580-G611	14	FLT3	
**chr13:28608221-28608221**	chr13	28608221	28608221	1	0	100	F612	14	FLT3	
**chr13:28608225-28608225**	chr13	28608225	28608225	1	0	100	E611	14	FLT3	
**chr13:28608231-28608231**	chr13	28608231	28608231	1	0	100	N609	14	FLT3	
**chr13:28608242-28608242**	chr13	28608242	28608242	1	0	100	F605	14	FLT3	
**chr13:28608255-28608255**	chr13	28608255	28608255	1	0	100	L601	14	FLT3	

**chr13:28608260-28608260**	chr13	28608260	28608260	1	0	100	Y599	14	FLT3
**chr13:28608270-28608270**	chr13	28608270	28608270	1	0	100	E596	14	FLT3
**chr13:28608276-28608276**	chr13	28608276	28608276	1	0	100	F594	14	FLT3
**chr13:28608281-28608281**	chr13	28608281	28608281	1	0	100	V592	14	FLT3
**chr13:28608317-28608317**	chr13	28608317	28608317	1	0	100	Q580	14	FLT3
**chr13:28608329-28608329**	chr13	28608329	28608329	1	0	100	L576	14	FLT3
**chr13:28608341-28608341**	chr13	28608341	28608341	1	0	100	Y572	14	FLT3
**chr15:90631837-90631837**	chr15	90631837	90631837	1	0	100	R172	4	IDH2
**chr15:90631837-90631839**	chr15	90631837	90631839	3	0	100	R172	4	IDH2
**chr15:90631838-90631838**	chr15	90631838	90631838	1	0	100	R172	4	IDH2
**chr15:90631933-90631935**	chr15	90631933	90631935	3	0	100	R140	4	IDH2
**chr15:90631934-90631934**	chr15	90631934	90631934	1	0	100	R140	4	IDH2
**chr17:7572955-7572955**	chr17	7572955	7572955	1	0	100	F346	11	TP53
**chr17:7572984-7572984**	chr17	7572984	7572984	1	0	100	S337	11	TP53
**chr17:7572987-7572987**	chr17	7572987	7572987	1	0	100	Q336	11	TP53
**chr17:7572989-7572989**	chr17	7572989	7572989	1	0	100	G335	11	TP53
**chr17:7573931-7573931**	chr17	7573931	7573931	1	0	100	S327	10	TP53
**chr17:7573954-7573954**	chr17	7573954	7573954	1	0	100	E319	10	TP53
**chr17:7574003-7574003**	chr17	7574003	7574003	1	0	100	R303	10	TP53
**chr17:7574018-7574018**	chr17	7574018	7574018	1	0	100	R298	10	TP53
**chr17:7576544-7576544**	chr17	7576544	7576544	1	0	100	Q292	10	TP53
**chr17:7576550-7576550**	chr17	7576550	7576550	1	0	100	Q278	10	TP53
**chr17:7576563-7576563**	chr17	7576563	7576563	1	0	100	Non-coding		TP53
**chr17:7576572-7576572**	chr17	7576572	7576572	1	0	100	Non-coding		TP53
**chr17:7576646-7576648**	chr17	7576646	7576648	3	0	100	Non-coding		TP53
**chr17:7576852-7576852**	chr17	7576852	7576852	1	0	100	Non-coding		TP53
**chr17:7576855-7576855**	chr17	7576855	7576855	1	0	100	Q292	9	TP53
**chr17:7576897-7576897**	chr17	7576897	7576897	0	1	0	Q278	9	TP53
**chr17:7577022-7577022**	chr17	7577022	7577022	1	0	100	R267	8	TP53
**chr17:7577046-7577046**	chr17	7577046	7577046	1	0	100	E259	8	TP53
**chr17:7577082-7577082**	chr17	7577082	7577082	1	0	100	E247	8	TP53
**chr17:7577083-7577085**	chr17	7577083	7577085	3	0	100	E246	8	TP53
**chr17:7577094-7577094**	chr17	7577094	7577094	1	0	100	R243	8	TP53
**chr17:7577095-7577097**	chr17	7577095	7577097	3	0	100	D242	8	TP53
**chr17:7577100-7577100**	chr17	7577100	7577100	1	0	100	R241	8	TP53
**chr17:7577105-7577105**	chr17	7577105	7577105	1	0	100	P239	8	TP53
**chr17:7577106-7577106**	chr17	7577106	7577106	1	0	100	P239	8	TP53
**chr17:7577107-7577109**	chr17	7577107	7577109	3	0	100	C238	8	TP53
**chr17:7577112-7577112**	chr17	7577112	7577112	1	0	100	A237	8	TP53
**chr17:7577113-7577115**	chr17	7577113	7577115	3	0	100	C236	8	TP53
**chr17:7577120-7577120**	chr17	7577120	7577120	1	0	100	R234	8	TP53
**chr17:7577124-7577124**	chr17	7577124	7577124	1	0	100	V233	8	TP53
**chr17:7577137-7577139**	chr17	7577137	7577139	3	0	100	R228	8	TP53
**chr17:7577140-7577142**	chr17	7577140	7577142	3	0	100	G227	8	TP53
**chr17:7577507-7577509**	chr17	7577507	7577509	3	0	100	E219	7	TP53
**chr17:7577516-7577518**	chr17	7577516	7577518	3	0	100	I216	7	TP53
**chr17:7577519-7577521**	chr17	7577519	7577521	3	0	100	I215	7	TP53

**chr17:7577530-7577530**	chr17	7577530	7577530	1	0	100	I212	7	TP53
**chr17:7577535-7577535**	chr17	7577535	7577535	1	0	100	R210	7	TP53
**chr17:7577538-7577539**	chr17	7577538	7577539	2	0	100	R209	7	TP53
**chr17:7577543-7577545**	chr17	7577543	7577545	3	0	100	G206	7	TP53
**chr17:7577547-7577547**	chr17	7577547	7577547	1	0	100	G206	7	TP53
**chr17:7577548-7577548**	chr17	7577548	7577548	1	0	100	G206	7	TP53
**chr17:7577549-7577551**	chr17	7577549	7577551	3	0	100	G205	7	TP53
**chr17:7577553-7577553**	chr17	7577553	7577553	1	0	100	M204	7	TP53
**chr17:7577555-7577557**	chr17	7577555	7577557	3	0	100	C203	7	TP53
**chr17:7577559-7577559**	chr17	7577559	7577559	1	0	100	S202	7	TP53
**chr17:7577561-7577563**	chr17	7577561	7577563	3	0	100	S201	7	TP53
**chr17:7577564-7577566**	chr17	7577564	7577566	3	0	100	N200	7	TP53
**chr17:7577567-7577569**	chr17	7577567	7577569	3	0	100	C199	7	TP53
**chr17:7577568-7577568**	chr17	7577568	7577568	1	0	100	C199	7	TP53
**chr17:7577570-7577570**	chr17	7577570	7577570	1	0	100	M198	7	TP53
**chr17:7577574-7577574**	chr17	7577574	7577574	1	0	100	Y197	7	TP53
**chr17:7577580-7577580**	chr17	7577580	7577580	1	0	100	Y195	7	TP53
**chr17:7577585-7577587**	chr17	7577585	7577587	3	0	100	I193	7	TP53
**chr17:7578181-7578181**	chr17	7578181	7578181	1	0	100	P184	6	TP53
**chr17:7578190-7578190**	chr17	7578190	7578190	1	0	100	Y181	6	TP53
**chr17:7578193-7578193**	chr17	7578193	7578193	1	0	100	P180	6	TP53
**chr17:7578203-7578203**	chr17	7578203	7578203	1	0	100	V177	6	TP53
**chr17:7578204-7578206**	chr17	7578204	7578206	3	0	100	S176	6	TP53
**chr17:7578208-7578208**	chr17	7578208	7578208	1	0	100	H175	6	TP53
**chr17:7578209-7578209**	chr17	7578209	7578209	1	0	100	H175	6	TP53
**chr17:7578212-7578212**	chr17	7578212	7578212	1	0	100	R174	6	TP53
**chr17:7578234-7578234**	chr17	7578234	7578234	1	0	100	Y166	6	TP53
**chr17:7578249-7578251**	chr17	7578249	7578251	3	0	100	N161	6	TP53
**chr17:7578263-7578263**	chr17	7578263	7578263	1	0	100	R157	6	TP53
**chr17:7578265-7578265**	chr17	7578265	7578265	1	0	100	I156	6	TP53
**chr17:7578268-7578268**	chr17	7578268	7578268	1	0	100	L155	6	TP53
**chr17:7578270-7578272**	chr17	7578270	7578272	3	0	100	H154	6	TP53
**chr17:7578275-7578275**	chr17	7578275	7578275	1	0	100	Q153	6	TP53
**chr17:7578394-7578394**	chr17	7578394	7578394	1	0	100	H140	5	TP53
**chr17:7578403-7578403**	chr17	7578403	7578403	1	0	100	C137	5	TP53
**chr17:7578406-7578406**	chr17	7578406	7578406	1	0	100	R136	5	TP53
**chr17:7578411-7578413**	chr17	7578411	7578413	3	0	100	V134	5	TP53
**chr17:7578435-7578437**	chr17	7578435	7578437	3	0	100	Q126	5	TP53
**chr17:7578442-7578442**	chr17	7578442	7578442	1	0	100	Y124	5	TP53
**chr17:7578444-7578446**	chr17	7578444	7578446	3	0	100	I123	5	TP53
**chr17:7578455-7578455**	chr17	7578455	7578455	1	0	100	A120	5	TP53
**chr17:7578457-7578457**	chr17	7578457	7578457	1	0	100	R119	5	TP53
**chr17:7578461-7578461**	chr17	7578461	7578461	1	0	100	V118	5	TP53
**chr17:7578475-7578475**	chr17	7578475	7578475	1	0	100	P113	5	TP53
**chr17:7578479-7578479**	chr17	7578479	7578479	1	0	100	P112	5	TP53
**chr17:7578492-7578492**	chr17	7578492	7578492	1	0	100	W107	5	TP53
**chr17:7578501-7578503**	chr17	7578501	7578503	3	0	100	V104	5	TP53

**chr17:7578508-7578508**	chr17	7578508	7578508	1	0	100	C102	5	TP53
**chr17:7578516-7578518**	chr17	7578516	7578518	3	0	100	A99	5	TP53
**chr17:7578522-7578524**	chr17	7578522	7578524	3	0	100	Q97	5	TP53
**chr17:7578525-7578527**	chr17	7578525	7578527	3	0	100	C96	5	TP53
**chr17:7578528-7578530**	chr17	7578528	7578530	3	0	100	F95	5	TP53
**chr17:7578535-7578535**	chr17	7578535	7578535	1	0	100	K93	5	TP53
**chr17:7578540-7578542**	chr17	7578540	7578542	3	0	100	L91	5	TP53
**chr17:7578549-7578550**	chr17	7578549	7578550	2	0	100	S88	5	TP53
**chr17:7578552-7578554**	chr17	7578552	7578554	3	0	100	Y87	5	TP53
**chr17:7579312-7579314**	chr17	7579312	7579314	3	0	100	T86	4	TP53
**chr17:7579330-7579331**	chr17	7579330	7579331	2	0	100	A80	4	TP53
**chr17:7579354-7579356**	chr17	7579354	7579356	3	0	100	L72	4	TP53
**chr17:7579358-7579358**	chr17	7579358	7579358	1	0	100	R71	4	TP53
**chr17:7579406-7579406**	chr17	7579406	7579406	1	0	100	S55	4	TP53
**chr17:7579415-7579415**	chr17	7579415	7579415	1	0	100	W52	4	TP53
**chr17:7579470-7579470**	chr17	7579470	7579470	1	0	100	V34	4	TP53
**chr17:7579472-7579472**	chr17	7579472	7579472	1	0	100	P33	4	TP53
**chr17:7579494-7579494**	chr17	7579494	7579494	1	0	100	R26	4	TP53
**chr17:7579541-7579541**	chr17	7579541	7579541	0	1	0	D10	4	TP53
**chr17:7579547-7579547**	chr17	7579547	7579547	0	1	0	D9	4	TP53
**chr17:7579558-7579558**	chr17	7579558	7579558	1	0	100	L4	4	TP53
**chr17:7579562-7579562**	chr17	7579562	7579562	1	0	100	D3	4	TP53
**chr17:7579584-7579584**	chr17	7579584	7579584	1	0	100	L35	4	TP53
**chr17:7579699-7579699**	chr17	7579699	7579699	1	0	100	Non-coding		TP53
**chr17:7579862-7579862**	chr17	7579862	7579862	1	0	100	Non-coding		TP53
**chr17:7579878-7579878**	chr17	7579878	7579878	1	0	100	Non-coding		TP53
**chr17:7579892-7579892**	chr17	7579892	7579892	1	0	100	Non-coding		TP53
**chr17:7579905-7579905**	chr17	7579905	7579905	1	0	100	Non-coding		TP53
**chr17:29483087-29483087**	chr17	29483087	29483087	1	0	100	Y49	2	TP53
**chr17:29483115-29483115**	chr17	29483115	29483115	1	0	100	T59	2	TP53
**chr17:29483145-29483145**	chr17	29483145	29483145	1	0	100	Non-coding		TP53
**chr17:29483145-29483148**	chr17	29483145	29483148	4	0	100	Non-coding		TP53
**chr17:29508733-29508738**	chr17	29508733	29508738	6	0	100	W221	7	TP53
**chr17:29508736-29508736**	chr17	29508736	29508736	1	0	100	N222	7	TP53
**chr17:29508805-29508805**	chr17	29508805	29508805	1	0	100	Non-coding		TP53
**chr17:29509552-29509590**	chr17	29509552	29509590	39	0	100	E258	8	TP53
**chr17:29509567-29509567**	chr17	29509567	29509567	1	0	100	E259	8	TP53
**chr17:29509596-29509596**	chr17	29509596	29509596	1	0	100	W267	8	TP53
**chr17:29528146-29528146**	chr17	29528146	29528146	1	0	100	R385	10	TP53
**chr17:29528489-29528489**	chr17	29528489	29528489	1	0	100	R416	11	TP53
**chr17:29528504-29528504**	chr17	29528504	29528504	1	0	100	Non-coding		TP53
**chr17:29533267-29533267**	chr17	29533267	29533267	1	0	100	D424	12	TP53
**chr17:29533355-29533355**	chr17	29533355	29533355	1	0	100	G453	12	TP53
**chr17:29533378-29533378**	chr17	29533378	29533378	1	0	100	R461	12	TP53
**chr17:29552113-29552113**	chr17	29552113	29552113	1	0	100	Q616	17	TP53
**chr17:29552221-29552221**	chr17	29552221	29552221	1	0	100	R462	17	TP53
**chr17:29553697-29553697**	chr17	29553697	29553697	1	0	100	S749	18	TP53

**chr17:29554277-29554277**	chr17	29554277	29554277	1	0	100	R765	19	TP53
**chr17:29554278-29554284**	chr17	29554278	29554284	7	0	100	I766	19	TP53
**chr17:29554283-29554283**	chr17	29554283	29554283	1	0	100	E767	19	TP53
**chr17:29554554-29554571**	chr17	29554554	29554571	18	0	100	H781-E785	20	TP53
**chr17:29554556-29554573**	chr17	29554556	29554573	18	0	100	H781-E785	20	TP53
**chr17:29554571-29554571**	chr17	29554571	29554571	1	0	100	Q786	20	TP53
**chr17:29556167-29556167**	chr17	29556167	29556167	1	0	100	C845	21	TP53
**chr17:29556167-29556190**	chr17	29556167	29556190	24	0	100	A846-L852	21	TP53
**chr17:29556247-29556247**	chr17	29556247	29556247	1	0	100	E872	21	TP53
**chr17:29556875-29556879**	chr17	29556875	29556879	5	0	100	T958-Q959	22	TP53
**chr17:29556877-29556877**	chr17	29556877	29556877	1	0	100	Q959	22	TP53
**chr17:29557871-29557871**	chr17	29557871	29557871	1	0	100	V1042	24	TP53
**chr17:29557890-29557890**	chr17	29557890	29557890	1	0	100	W1048	24	TP53
**chr17:29560043-29560043**	chr17	29560043	29560043	1	0	100	Q1174	27	TP53
**chr17:29560071-29560071**	chr17	29560071	29560071	1	0	100	L1183	27	TP53
**chr17:29562638-29562641**	chr17	29562638	29562641	4	0	100	A1240	28	TP53
**chr17:29562641-29562641**	chr17	29562641	29562641	1	0	100	R1241	28	TP53
**chr17:29562747-29562747**	chr17	29562747	29562747	1	0	100	R1276	28	TP53
**chr17:29562981-29562981**	chr17	29562981	29562981	1	0	100	R1306	29	TP53
**chr17:29563007-29563007**	chr17	29563007	29563007	1	0	100	W1314	29	TP53
**chr17:29576111-29576111**	chr17	29576111	29576111	1	0	100	R1362	30	TP53
**chr17:29588751-29588751**	chr17	29588751	29588751	1	0	100	R1534	35	TP53
**chr17:29652868-29652912**	chr17	29652868	29652912	45	0	100	L1623	37	TP53
**chr17:29652882-29652882**	chr17	29652882	29652882	1	0	100	V1627	37	TP53
**chr17:29654548-29654565**	chr17	29654548	29654565	18	0	100	A1767	38	TP53
**chr17:29654553-29654553**	chr17	29654553	29654553	1	0	100	R1769	38	TP53
**chr17:29654664-29654664**	chr17	29654664	29654664	1	0	100	Q1806	38	TP53
**chr17:29657375-29657375**	chr17	29657375	29657375	1	0	100	Q1891	39	TP53
**chr17:29663734-29663734**	chr17	29663734	29663734	1	0	100	D2077	42	TP53
**chr17:29667527-29667527**	chr17	29667527	29667527	1	0	100	S2309	47	TP53
**chr17:29684004-29684004**	chr17	29684004	29684004	1	0	100	Q2589	53	TP53
**chr17:29684085-29684085**	chr17	29684085	29684085	1	0	100	Q2616	53	TP53
**chr17:29684113-29684113**	chr17	29684113	29684113	1	0	100	Non-coding		TP53
**chr17:29684287-29684287**	chr17	29684287	29684287	1	0	100	A2624	54	TP53
**chr17:29685616-29685631**	chr17	29685616	29685631	16	0	100	Q2697-S270	55	TP53
**chr17:29685622-29685622**	chr17	29685622	29685622	1	0	100	Q2699		TP53
**chr17:40474418-40474420**	chr17	40474418	40474420	3	0	100	D661	21	STAT3
**chr17:40474460-40474462**	chr17	40474460	40474462	3	0	100	N647	21	STAT3
**chr17:40474481-40474483**	chr17	40474481	40474483	3	0	100	Y640	21	STAT3
**chr17:40475058-40475058**	chr17	40475058	40475058	1	0	100	G618	20	STAT3
**chr17:40475064-40475066**	chr17	40475064	40475066	3	0	100	K615	20	STAT3
**chr17:40475068-40475068**	chr17	40475068	40475068	1	0	100	S614	20	STAT3
**chr17:40475328-40475330**	chr17	40475328	40475330	3	0	100	D566	19	STAT3
**chr17:58740432-58740432**	chr17	58740432	58740432	1	0	100	S446	6	PPM1D
**chr17:58740527-58740529**	chr17	58740527	58740529	3	0	100	C478	6	PPM1D
**chr17:58740545-58740547**	chr17	58740545	58740547	3	0	100	L484	6	PPM1D
**chr17:58740749-58740749**	chr17	58740749	58740749	1	0	100	R552	6	PPM1D

**chr17:58740749-58740751**	chr17	58740749	58740751	3	0	100	R552	6	PPM1D	
**chr17:58740809-58740811**	chr17	58740809	58740811	3	0	100	R572	6	PPM1D	
**chr17:74732956-74732961**	chr17	74732956	74732961	0	6	0	P95	1	SRSF2	
**chr18:42530432-42530432**	chr18	42530432	42530432	1	0	100	S376	4	SETBP1	
**chr18:42531877-42531877**	chr18	42531877	42531877	1	0	100	E858	4	SETBP1	
**chr18:42531906-42531906**	chr18	42531906	42531906	1	0	100	S867	4	SETBP1	
**chr18:42531907-42531907**	chr18	42531907	42531907	1	0	100	D868	4	SETBP1	
**chr18:42531910-42531912**	chr18	42531910	42531912	3	0	100	S869	4	SETBP1	
**chr18:42531913-42531913**	chr18	42531913	42531913	1	0	100	G870	4	SETBP1	
**chr18:42531917-42531917**	chr18	42531917	42531917	1	0	100	I871	4	SETBP1	
**chr18:42531925-42531927**	chr18	42531925	42531927	3	0	100	D874	4	SETBP1	
**chr18:42532022-42532022**	chr18	42532022	42532022	1	0	100	P906	4	SETBP1	
**chr18:42532606-42532606**	chr18	42532606	42532606	1	0	100	V1101	4	SETBP1	
**chr18:42532693-42532693**	chr18	42532693	42532693	1	0	100	P1130	4	SETBP1	
**chr18:42533036-42533036**	chr18	42533036	42533036	1	0	100	Q1244	4	SETBP1	
**chr18:42533044-42533044**	chr18	42533044	42533044	1	0	100	E1247	4	SETBP1	
**chr18:42533111-42533111**	chr18	42533111	42533111	1	0	100	D1269	4	SETBP1	
**chr18:42533165-42533165**	chr18	42533165	42533165	1	0	100	S12187	4	SETBP1	
**chr18:42533293-42533293**	chr18	42533293	42533293	1	0	100	S1330	4	SETBP1	
**chr19:8976273-8976275**	chr19	8976273	8976275	3	0	100	S14185	75	MUC16	
**chr19:8982188-8982190**	chr19	8982188	8982190	3	0	100	Y14029	70	MUC16	
**chr19:8999450-8999452**	chr19	8999450	8999452	3	0	100	T13575	56	MUC16	
**chr19:8999486-8999488**	chr19	8999486	8999488	3	0	100	D13563	56	MUC16	
**chr19:9028372-9028374**	chr19	9028372	9028374	3	0	100	T12140	11	MUC16	
**chr19:13054564-13054615**	chr19	13054564	13054615	52	0	100	E364-E381	9	CALR	
**chr19:13054566-13054568**	chr19	13054566	13054568	3	0	100	Q365	9	CALR	
**chr19:13054593-13054596**	chr19	13054593	13054596	4	0	100	K374	9	CALR	
**chr19:13054628-13054628**	chr19	13054628	13054628	1	0	100	K385	9	CALR	
**chr19:33792358-33792360**	chr19	33792358	33792360	3	0	100	N321	1	CEBPA	
**chr19:33792385-33792387**	chr19	33792385	33792387	3	0	100	Q312	1	CEBPA	
**chr19:33792397-33792399**	chr19	33792397	33792399	3	0	100	V308	1	CEBPA	
**chr19:33792421-33792423**	chr19	33792421	33792423	3	0	100	R300	1	CEBPA	
**chr19:33792430-33792432**	chr19	33792430	33792432	3	0	100	R297	1	CEBPA	
**chr19:33792436-33792438**	chr19	33792436	33792438	3	0	100	A295	1	CEBPA	
**chr19:33792739-33792741**	chr19	33792739	33792741	0	3	0	P194	1	CEBPA	
**chr19:33792778-33792780**	chr19	33792778	33792780	0	3	0	Y181	1	CEBPA	
**chr19:33792781-33792783**	chr19	33792781	33792783	0	3	0	P180	1	CEBPA	
**chr19:33792886-33792888**	chr19	33792886	33792888	0	3	0	P145	1	CEBPA	
**chr19:33793072-33793074**	chr19	33793072	33793074	0	3	0	Q83	1	CEBPA	
**chr19:33793144-33793146**	chr19	33793144	33793146	0	3	0	E59	1	CEBPA	
**chr20:31021211-31021213**	chr20	31021211	31021213	3	0	100	R404	12	ASXL1	
**chr20:31021250-31021252**	chr20	31021250	31021252	3	0	100	R417	12	ASXL1	
**chr20:31022277-31022279**	chr20	31022277	31022279	3	0	100	Q588	13	ASXL1	
**chr20:31022286-31022288**	chr20	31022286	31022288	3	0	100	Y591	13	ASXL1	
**chr20:31022289-31022291**	chr20	31022289	31022291	3	0	100	Q592	13	ASXL1	
**chr20:31022450-31022450**	chr20	31022450	31022450	1	0	100	G645	13	ASXL1	
**chr20:31022469-31022469**	chr20	31022469	31022469	1	0	100	G652	13	ASXL1	

**chr20:31022484-31022484**	chr20	31022484	31022484	1	0	100	E657	13	ASXL1
**chr20:31022487-31022487**	chr20	31022487	31022487	1	0	100	G658	13	ASXL1
**chr20:31022550-31022550**	chr20	31022550	31022550	1	0	100	G679	13	ASXL1
**chr20:31022592-31022594**	chr20	31022592	31022594	3	0	100	R693	13	ASXL1
**chr20:31022712-31022712**	chr20	31022712	31022712	1	0	100	Q733	13	ASXL1
**chr20:31022757-31022757**	chr20	31022757	31022757	1	0	100	Q748	13	ASXL1
**chr20:31022817-31022817**	chr20	31022817	31022817	1	0	100	Q768	13	ASXL1
**chr20:31022839-31022839**	chr20	31022839	31022839	1	0	100	L775	13	ASXL1
**chr20:31022847-31022847**	chr20	31022847	31022847	1	0	100	Q778	13	ASXL1
**chr20:31022899-31022899**	chr20	31022899	31022899	1	0	100	S795	13	ASXL1
**chr20:31022937-31022937**	chr20	31022937	31022937	1	0	100	P808	13	ASXL1
**chr20:31023000-31023000**	chr20	31023000	31023000	1	0	100	Q829	13	ASXL1
**chr20:31023027-31023027**	chr20	31023027	31023027	1	0	100	K838	13	ASXL1
**chr20:31023087-31023087**	chr20	31023087	31023087	1	0	100	Q858	13	ASXL1
**chr20:31023240-31023240**	chr20	31023240	31023240	1	0	100	K909	13	ASXL1
**chr20:31023294-31023294**	chr20	31023294	31023294	1	0	100	G927	13	ASXL1
**chr20:31023385-31023385**	chr20	31023385	31023385	1	0	100	T957	13	ASXL1
**chr20:31023395-31023395**	chr20	31023395	31023395	1	0	100	W960	13	ASXL1
**chr20:31023408-31023408**	chr20	31023408	31023408	1	0	100	R965	13	ASXL1
**chr20:31023409-31023411**	chr20	31023409	31023411	3	0	100	G967	13	ASXL1
**chr20:31023527-31023527**	chr20	31023527	31023527	1	0	100	D1004	13	ASXL1
**chr20:31023598-31023598**	chr20	31023598	31023598	1	0	100	S1028	13	ASXL1
**chr20:31023630-31023630**	chr20	31023630	31023630	1	0	100	Q1039	13	ASXL1
**chr20:31023717-31023717**	chr20	31023717	31023717	1	0	100	Q1063	13	ASXL1
**chr20:31023732-31023732**	chr20	31023732	31023732	1	0	100	R1068	13	ASXL1
**chr20:31023821-31023821**	chr20	31023821	31023821	1	0	100	E1102	13	ASXL1
**chr20:31023834-31023834**	chr20	31023834	31023834	1	0	100	L1107	13	ASXL1
**chr20:31024018-31024018**	chr20	31024018	31024018	1	0	100	S1168	13	ASXL1
**chr20:31024021-31024021**	chr20	31024021	31024021	1	0	100	S1169	13	ASXL1
**chr20:31024212-31024212**	chr20	31024212	31024212	1	0	100	E1228	13	ASXL1
**chr20:31024251-31024251**	chr20	31024251	31024251	1	0	100	E1241	13	ASXL1
**chr20:31024291-31024291**	chr20	31024291	31024291	1	0	100	P1259	13	ASXL1
**chr20:31024332-31024332**	chr20	31024332	31024332	1	0	100	R1273	13	ASXL1
**chr20:31024339-31024339**	chr20	31024339	31024339	1	0	100	S1275	13	ASXL1
**chr20:31024698-31024698**	chr20	31024698	31024698	1	0	100	L1395	13	ASXL1
**chr20:31024704-31024704**	chr20	31024704	31024704	1	0	100	G1397	13	ASXL1
**chr20:31024758-31024758**	chr20	31024758	31024758	1	0	100	R1415	13	ASXL1
**chr20:31025035-31025035**	chr20	31025035	31025035	1	0	100	A1507	13	ASXL1
**chr20:57429108-57429110**	chr20	57429108	57429110	3	0	100	A263	1	GNAS
**chr20:57484406-57484406**	chr20	57484406	57484406	1	0	100	D839	8	GNAS
**chr20:57484414-57484414**	chr20	57484414	57484414	1	0	100	R842	8	GNAS
**chr20:57484418-57484422**	chr20	57484418	57484422	5	0	100	R844	8	GNAS
**chr20:57484420-57484420**	chr20	57484420	57484420	1	0	100	C843-R844	8	GNAS
**chr20:57484420-57484422**	chr20	57484420	57484422	3	0	100	R844	8	GNAS
**chr20:57484421-57484421**	chr20	57484421	57484421	1	0	100	R844	8	GNAS
**chr20:57485133-57485135**	chr20	57485133	57485135	3	0	100	D966	11	GNAS
**chr21:36164685-36164685**	chr21	36164685	36164685	1	0	100	Q397	9	RUNX1

**chr21:36164703-36164703**	chr21	36164703	36164703	1	0	100	A391	9	RUNX1
**chr21:36164727-36164739**	chr21	36164727	36164739	13	0	100	T379-P383	9	RUNX1
**chr21:36164805-36164805**	chr21	36164805	36164805	1	0	100	P357	9	RUNX1
**chr21:36171600-36171600**	chr21	36171600	36171600	1	0	100	S322	8	RUNX1
**chr21:36171607-36171607**	chr21	36171607	36171607	1	0	100	R320	8	RUNX1
**chr21:36171623-36171624**	chr21	36171623	36171624	2	0	100	S314	8	RUNX1
**chr21:36171674-36171674**	chr21	36171674	36171674	1	0	100	H297	8	RUNX1
**chr21:36193979-36193979**	chr21	36193979	36193979	1	0	100	Non-coding		RUNX1
**chr21:36206706-36206706**	chr21	36206706	36206706	1	0	100	Non-coding		RUNX1
**chr21:36206711-36206711**	chr21	36206711	36206711	1	0	100	M267	7	RUNX1
**chr21:36206716-36206716**	chr21	36206716	36206716	1	0	100	Q266	7	RUNX1
**chr21:36206723-36206725**	chr21	36206723	36206725	3	0	100	P263	7	RUNX1
**chr21:36206762-36206764**	chr21	36206762	36206764	3	0	100	R250	7	RUNX1
**chr21:36206764-36206764**	chr21	36206764	36206764	1	0	100	R250	7	RUNX1
**chr21:36206775-36206775**	chr21	36206775	36206775	1	0	100	T246	7	RUNX1
**chr21:36206818-36206818**	chr21	36206818	36206818	1	0	100	R232	7	RUNX1
**chr21:36206861-36206863**	chr21	36206861	36206863	3	0	100	G217	7	RUNX1
**chr21:36206891-36206893**	chr21	36206891	36206893	3	0	100	R207	7	RUNX1
**chr21:36231774-36231774**	chr21	36231774	36231774	1	0	100	R204	6	RUNX1
**chr21:36231783-36231783**	chr21	36231783	36231783	1	0	100	R201	6	RUNX1
**chr21:36231791-36231791**	chr21	36231791	36231791	1	0	100	D198	6	RUNX1
**chr21:36231792-36231792**	chr21	36231792	36231792	1	0	100	D198	6	RUNX1
**chr21:36252853-36252853**	chr21	36252853	36252853	1	0	100	Non-coding		RUNX1
**chr21:36252858-36252860**	chr21	36252858	36252860	3	0	100	G168	5	RUNX1
**chr21:36252866-36252866**	chr21	36252866	36252866	1	0	100	R166	5	RUNX1
**chr21:36252869-36252869**	chr21	36252869	36252869	1	0	100	G165	5	RUNX1
**chr21:36252877-36252877**	chr21	36252877	36252877	1	0	100	R162	5	RUNX1
**chr21:36252878-36252878**	chr21	36252878	36252878	1	0	100	R162	5	RUNX1
**chr21:36252880-36252880**	chr21	36252880	36252880	1	0	100	L161	5	RUNX1
**chr21:36252882-36252884**	chr21	36252882	36252884	3	0	100	D160	5	RUNX1
**chr21:36252924-36252926**	chr21	36252924	36252926	3	0	100	N146	5	RUNX1
**chr21:36252940-36252940**	chr21	36252940	36252940	1	0	100	S141	5	RUNX1
**chr21:36259152-36259154**	chr21	36259152	36259154	1	2	33.33333333	P113	4	RUNX1
**chr21:36259161-36259163**	chr21	36259161	36259163	3	0	100	K110	4	RUNX1
**chr21:36259172-36259172**	chr21	36259172	36259172	1	0	100	R107	4	RUNX1
**chr21:36259173-36259173**	chr21	36259173	36259173	1	0	100	W106	4	RUNX1
**chr21:36259192-36259192**	chr21	36259192	36259192	1	0	100	S100	4	RUNX1
**chr21:36259199-36259199**	chr21	36259199	36259199	1	0	100	L98	4	RUNX1
**chr21:36259236-36259238**	chr21	36259236	36259238	3	0	100	H85	4	RUNX1
**chr21:36259294-36259294**	chr21	36259294	36259294	1	0	100	A66	4	RUNX1
**chr21:36259308-36259310**	chr21	36259308	36259310	3	0	100	P61	4	RUNX1
**chr21:36259324-36259324**	chr21	36259324	36259324	1	0	100	L56	4	RUNX1
**chr21:36421143-36421143**	chr21	36421143	36421143	1	0	100	M18	2	RUNX1
**chr21:36421162-36421162**	chr21	36421162	36421162	1	0	100	S12	2	RUNX1
**chr21:36421175-36421175**	chr21	36421175	36421175	1	0	100	E8	2	RUNX1
**chr21:39755585-39755585**	chr21	39755585	39755585	0	1	0	G394	10	ERG
**chr21:39755678-39755678**	chr21	39755678	39755678	1	0	100	D363	10	ERG

**chr21:39755732-39755732**	chr21	39755732	39755732	1	0	100	D345	10	ERG
**chr21:39755810-39755810**	chr21	39755810	39755810	1	0	100	E319	10	ERG
**chr21:39755822-39755822**	chr21	39755822	39755822	1	0	100	Q315	10	ERG
**chr21:39755827-39755827**	chr21	39755827	39755827	1	0	100	L313	10	ERG
**chr21:44513242-44513244**	chr21	44513242	44513244	3	0	100	S231	6	U2AF1
**chr21:44514604-44514606**	chr21	44514604	44514606	3	0	100	E184	6	U2AF1
**chr21:44514777-44514777**	chr21	44514777	44514777	1	0	100	Q157	6	U2AF1
**chr21:44514780-44514780**	chr21	44514780	44514780	1	0	100	R156	6	U2AF1
**chr21:44514831-44514831**	chr21	44514831	44514831	0	1	0	P139	6	U2AF1
**chr21:44514845-44514845**	chr21	44514845	44514845	1	0	100	W134	6	U2AF1
**chr21:44524453-44524453**	chr21	44524453	44524453	1	0	100	R35	2	U2AF1
**chr21:44524456-44524456**	chr21	44524456	44524456	1	0	100	S34	2	U2AF1
**chr21:44524472-44524472**	chr21	44524472	44524472	1	0	100	H29	2	U2AF1
**chr21:44524474-44524474**	chr21	44524474	44524474	1	0	100	R28	2	U2AF1
**chr21:44524486-44524486**	chr21	44524486	44524486	1	0	100	I24	2	U2AF1
**chr22:29083910-29083910**	chr22	29083910	29083910	1	0	100	P579	16	CHEK2
**chr22:29083949-29083949**	chr22	29083949	29083949	1	0	100	R566	16	CHEK2
**chr22:29083956-29083956**	chr22	29083956	29083956	1	0	100	R564	16	CHEK2
**chr22:29083961-29083961**	chr22	29083961	29083961	1	0	100	R562	16	CHEK2
**chr22:29083962-29083962**	chr22	29083962	29083962	1	0	100	R562	16	CHEK2
**chr22:29091724-29091724**	chr22	29091724	29091724	1	0	100	W545	12	CHEK2
**chr22:29091764-29091764**	chr22	29091764	29091764	1	0	100	S441	12	CHEK2
**chr22:29091788-29091788**	chr22	29091788	29091788	1	0	100	Y433	12	CHEK2
**chr22:29091816-29091816**	chr22	29091816	29091816	1	0	100	M424	12	CHEK2
**chr22:29091840-29091840**	chr22	29091840	29091840	1	0	100	K416	12	CHEK2
**chrX:15809095-15809095**	chrX	15809095	15809095	1	0	100	R27	2	ZRSR2
**chrX:15809121-15809121**	chrX	15809121	15809121	1	0	100	R36	2	ZRSR2
**chrX:15818015-15818015**	chrX	15818015	15818015	1	0	100	E48	3	ZRSR2
**chrX:15821884-15821884**	chrX	15821884	15821884	1	0	100	E93	4	ZRSR2
**chrX:15821891-15821891**	chrX	15821891	15821891	1	0	100	A95	4	ZRSR2
**chrX:15822289-15822289**	chrX	15822289	15822289	1	0	100	E123	5	ZRSR2
**chrX:15822297-15822297**	chrX	15822297	15822297	1	0	100	R126	5	ZRSR2
**chrX:15822319-15822319**	chrX	15822319	15822319	1	0	100	E133	5	ZRSR2
**chrX:15826383-15826383**	chrX	15826383	15826383	1	0	100	A143	6	ZRSR2
**chrX:15827343-15827343**	chrX	15827343	15827343	1	0	100	W153	7	ZRSR2
**chrX:15827344-15827344**	chrX	15827344	15827344	1	0	100	Q154	7	ZRSR2
**chrX:15827389-15827389**	chrX	15827389	15827389	1	0	100	R169	7	ZRSR2
**chrX:15827426-15827426**	chrX	15827426	15827426	1	0	100	C181	7	ZRSR2
**chrX:15827433-15827433**	chrX	15827433	15827433	1	0	100	F183	7	ZRSR2
**chrX:15833873-15833873**	chrX	15833873	15833873	1	0	100	M211	8	ZRSR2
**chrX:15833879-15833879**	chrX	15833879	15833879	1	0	100	Q213	8	ZRSR2
**chrX:15833945-15833945**	chrX	15833945	15833945	1	0	100	Q235	8	ZRSR2
**chrX:15833957-15833959**	chrX	15833957	15833959	3	0	100	F239	8	ZRSR2
**chrX:15838340-15838340**	chrX	15838340	15838340	1	0	100	C280	10	ZRSR2
**chrX:15838343-15838343**	chrX	15838343	15838343	1	0	100	Q281	10	ZRSR2
**chrX:15838370-15838372**	chrX	15838370	15838372	3	0	100	R290	10	ZRSR2
**chrX:15838385-15838385**	chrX	15838385	15838385	1	0	100	R295	10	ZRSR2

**chrX:15838410-15838410**	chrX	15838410	15838410	1	0	100	P303	10	ZRSR2
**chrX:15838412-15838412**	chrX	15838412	15838412	1	0	100	V304	10	ZRSR2
**chrX:15838419-15838419**	chrX	15838419	15838419	1	0	100	R306	10	ZRSR2
**chrX:15840936-15840936**	chrX	15840936	15840936	1	0	100	W340	11	ZRSR2
**chrX:15841096-15841096**	chrX	15841096	15841096	1	0	100	E394	11	ZRSR2
**chrX:15841213-15841213**	chrX	15841213	15841213	1	0	100	R433	11	ZRSR2
**chrX:15841227-15841233**	chrX	15841227	15841233	7	0	100	G438	11	ZRSR2
**chrX:15841228-15841228**	chrX	15841228	15841228	1	0	100	G438	11	ZRSR2
**chrX:15841255-15841257**	chrX	15841255	15841257	3	0	100	S447	11	ZRSR2
**chrX:15841258-15841260**	chrX	15841258	15841260	3	0	100	R448	11	ZRSR2
**chrX:15841270-15841270**	chrX	15841270	15841270	1	0	100	R452	11	ZRSR2
**chrX:15841301-15841301**	chrX	15841301	15841301	1	0	100	R462	11	ZRSR2
**chrX:15841306-15841306**	chrX	15841306	15841306	1	0	100	R464	11	ZRSR2
**chrX:39911589-39911589**	chrX	39911589	39911589	1	0	100	R1681	15	BCOR
**chrX:39911597-39911597**	chrX	39911597	39911597	1	0	100	R1678	15	BCOR
**chrX:39911647-39911649**	chrX	39911647	39911649	3	0	100	R1661	15	BCOR
**chrX:39913151-39913151**	chrX	39913151	39913151	1	0	100	S1655	14	BCOR
**chrX:39913510-39913510**	chrX	39913510	39913510	1	0	100	C1606	13	BCOR
**chrX:39914631-39914631**	chrX	39914631	39914631	1	0	100	K1577	12	BCOR
**chrX:39914723-39914723**	chrX	39914723	39914723	1	0	100	R1547	12	BCOR
**chrX:39914756-39914756**	chrX	39914756	39914756	1	0	100	D1536	12	BCOR
**chrX:39916463-39916463**	chrX	39916463	39916463	1	0	100	R1514	11	BCOR
**chrX:39916531-39916531**	chrX	39916531	39916531	1	0	100	N1491	11	BCOR
**chrX:39921444-39921444**	chrX	39921444	39921444	1	0	100	N1459	11	BCOR
**chrX:39921477-39921480**	chrX	39921477	39921480	4	0	100	R1447-P144	10	BCOR
**chrX:39921504-39921504**	chrX	39921504	39921504	1	0	100	S1439	10	BCOR
**chrX:39922048-39922048**	chrX	39922048	39922048	1	0	100	R1375	9	BCOR
**chrX:39922052-39922052**	chrX	39922052	39922052	1	0	100	R1374	9	BCOR
**chrX:39922061-39922061**	chrX	39922061	39922061	1	0	100	Q1371	9	BCOR
**chrX:39922141-39922141**	chrX	39922141	39922141	1	0	100	A1344	9	BCOR
**chrX:39922198-39922198**	chrX	39922198	39922198	1	0	100	K1325	9	BCOR
**chrX:39922208-39922208**	chrX	39922208	39922208	1	0	100	Q1322	9	BCOR
**chrX:39922231-39922231**	chrX	39922231	39922231	1	0	100	A1314	9	BCOR
**chrX:39922906-39922906**	chrX	39922906	39922906	1	0	100	R1268	8	BCOR
**chrX:39923055-39923055**	chrX	39923055	39923055	1	0	100	W1218	8	BCOR
**chrX:39923059-39923059**	chrX	39923059	39923059	1	0	100	R1217	8	BCOR
**chrX:39923087-39923087**	chrX	39923087	39923087	1	0	100	K1207	8	BCOR
**chrX:39923143-39923143**	chrX	39923143	39923143	1	0	100	S1189	8	BCOR
**chrX:39923601-39923601**	chrX	39923601	39923601	1	0	100	R1164	7	BCOR
**chrX:39923684-39923684**	chrX	39923684	39923684	1	0	100	R1136	7	BCOR
**chrX:39923700-39923700**	chrX	39923700	39923700	1	0	100	R1131	7	BCOR
**chrX:39923763-39923763**	chrX	39923763	39923763	0	1	0	Q1110	7	BCOR
**chrX:39930904-39930904**	chrX	39930904	39930904	1	0	100	A1013	5	BCOR
**chrX:39930913-39930913**	chrX	39930913	39930913	1	0	100	C1010	5	BCOR
**chrX:39930940-39930940**	chrX	39930940	39930940	1	0	100	E1001	5	BCOR
**chrX:39931619-39931619**	chrX	39931619	39931619	1	0	100	E994	4	BCOR
**chrX:39931668-39931668**	chrX	39931668	39931668	1	0	100	F977	4	BCOR

**chrX:39931909-39931909**	chrX	39931909	39931909	1	0	100	S897	4	BCOR
**chrX:39932320-39932320**	chrX	39932320	39932320	1	0	100	L760	4	BCOR
**chrX:39932323-39932323**	chrX	39932323	39932323	1	0	100	T759	4	BCOR
**chrX:39932333-39932333**	chrX	39932333	39932333	1	0	100	E756	4	BCOR
**chrX:39932496-39932496**	chrX	39932496	39932496	1	0	100	L702	4	BCOR
**chrX:39932513-39932513**	chrX	39932513	39932513	1	0	100	L696	4	BCOR
**chrX:39932591-39932591**	chrX	39932591	39932591	1	0	100	P670	4	BCOR
**chrX:39932628-39932628**	chrX	39932628	39932628	1	0	100	Y657	4	BCOR
**chrX:39932638-39932638**	chrX	39932638	39932638	1	0	100	P654	4	BCOR
**chrX:39932711-39932711**	chrX	39932711	39932711	1	0	100	E630	4	BCOR
**chrX:39932750-39932750**	chrX	39932750	39932750	1	0	100	A617	4	BCOR
**chrX:39932794-39932794**	chrX	39932794	39932794	1	0	100	P602	4	BCOR
**chrX:39932953-39932953**	chrX	39932953	39932953	1	0	100	G549	4	BCOR
**chrX:39932980-39932980**	chrX	39932980	39932980	1	0	100	R540	4	BCOR
**chrX:39933022-39933022**	chrX	39933022	39933022	1	0	100	S526	4	BCOR
**chrX:39933036-39933036**	chrX	39933036	39933036	1	0	100	N521	4	BCOR
**chrX:39933338-39933338**	chrX	39933338	39933338	1	0	100	G421	4	BCOR
**chrX:39933535-39933535**	chrX	39933535	39933535	0	1	0	S355	4	BCOR
**chrX:39933563-39933563**	chrX	39933563	39933563	0	1	0	P346	4	BCOR
**chrX:39933575-39933575**	chrX	39933575	39933575	0	1	0	R342	4	BCOR
**chrX:39933809-39933809**	chrX	39933809	39933809	1	0	100	P264	4	BCOR
**chrX:39933842-39933842**	chrX	39933842	39933842	1	0	100	V253	4	BCOR
**chrX:39933858-39933858**	chrX	39933858	39933858	1	0	100	P248	4	BCOR
**chrX:39933871-39933871**	chrX	39933871	39933871	1	0	100	R243	4	BCOR
**chrX:39934301-39934302**	chrX	39934301	39934302	2	0	100	K100	4	BCOR
**chrX:39934426-39934426**	chrX	39934426	39934426	1	0	100	A58	4	BCOR
**chrX:44820637-44820637**	chrX	44820637	44820637	1	0	100	A112	3	KDM6A
**chrX:44833919-44833919**	chrX	44833919	44833919	1	0	100	A115	4	KDM6A
**chrX:44833925-44833925**	chrX	44833925	44833925	1	0	100	Q117	4	KDM6A
**chrX:44833943-44833943**	chrX	44833943	44833943	1	0	100	Q123	4	KDM6A
**chrX:44833954-44833954**	chrX	44833954	44833954	1	0	100	Y126	4	KDM6A
**chrX:44833960-44833960**	chrX	44833960	44833960	1	0	100	K128	4	KDM6A
**chrX:44879904-44879904**	chrX	44879904	44879904	1	0	100	R165	6	KDM6A
**chrX:44879925-44879925**	chrX	44879925	44879925	1	0	100	R172	6	KDM6A
**chrX:44879971-44879971**	chrX	44879971	44879971	1	0	100	L187	6	KDM6A
**chrX:44896915-44896915**	chrX	44896915	44896915	1	0	100	A212	8	KDM6A
**chrX:44911017-44911017**	chrX	44911017	44911017	1	0	100	Q240	9	KDM6A
**chrX:44913136-44913136**	chrX	44913136	44913136	1	0	100	Q271	10	KDM6A
**chrX:44913148-44913148**	chrX	44913148	44913148	1	0	100	K275	10	KDM6A
**chrX:44918259-44918259**	chrX	44918259	44918259	1	0	100	S295	11	KDM6A
**chrX:44918269-44918269**	chrX	44918269	44918269	1	0	100	V300	11	KDM6A
**chrX:44918276-44918276**	chrX	44918276	44918276	1	0	100	Q301	11	KDM6A
**chrX:44918277-44918277**	chrX	44918277	44918277	1	0	100	Q301	11	KDM6A
**chrX:44918312-44918312**	chrX	44918312	44918312	1	0	100	K313	11	KDM6A
**chrX:44918338-44918338**	chrX	44918338	44918338	1	0	100	W321	11	KDM6A
**chrX:44919382-44919382**	chrX	44919382	44919382	1	0	100	A437	13	KDM6A
**chrX:44921908-44921908**	chrX	44921908	44921908	1	0	100	R481	15	KDM6A

**chrX:44921916-44921916**	chrX	44921916	44921916	1	0	100	R484	15	KDM6A
**chrX:44921960-44921960**	chrX	44921960	44921960	1	0	100	M505	15	KDM6A
**chrX:44922931-44922931**	chrX	44922931	44922931	1	0	100	I598	16	KDM6A
**chrX:44922958-44922958**	chrX	44922958	44922958	1	0	100	V607	16	KDM6A
**chrX:44922970-44922970**	chrX	44922970	44922970	1	0	100	Q611	16	KDM6A
**chrX:44923000-44923000**	chrX	44923000	44923000	1	0	100	R621	16	KDM6A
**chrX:44923035-44923035**	chrX	44923035	44923035	1	0	100	W632	16	KDM6A
**chrX:44928935-44928935**	chrX	44928935	44928935	0	1	0	Q679	17	KDM6A
**chrX:44929008-44929008**	chrX	44929008	44929008	1	0	100	T703	17	KDM6A
**chrX:44929028-44929028**	chrX	44929028	44929028	1	0	100	Q710	17	KDM6A
**chrX:44929099-44929099**	chrX	44929099	44929099	1	0	100	H733	17	KDM6A
**chrX:44929160-44929160**	chrX	44929160	44929160	1	0	100	M754	17	KDM6A
**chrX:44929164-44929164**	chrX	44929164	44929164	1	0	100	T755	17	KDM6A
**chrX:44929280-44929280**	chrX	44929280	44929280	1	0	100	T794	17	KDM6A
**chrX:44929487-44929487**	chrX	44929487	44929487	1	0	100	Q863	17	KDM6A
**chrX:44936012-44936014**	chrX	44936012	44936014	3	0	100	S925	18	KDM6A
**chrX:44938447-44938447**	chrX	44938447	44938447	1	0	100	E999	20	KDM6A
**chrX:44942710-44942713**	chrX	44942710	44942713	4	0	100	K1097-L109	23	KDM6A
**chrX:44942724-44942724**	chrX	44942724	44942724	1	0	100	E1102	23	KDM6A
**chrX:44942752-44942752**	chrX	44942752	44942752	1	0	100	R1111	23	KDM6A
**chrX:44942757-44942759**	chrX	44942757	44942759	3	0	100	V1113	23	KDM6A
**chrX:44942761-44942761**	chrX	44942761	44942761	1	0	100	S1114	23	KDM6A
**chrX:44942817-44942817**	chrX	44942817	44942817	1	0	100	Q1133	23	KDM6A
**chrX:44942839-44942839**	chrX	44942839	44942839	1	0	100	G1140	23	KDM6A
**chrX:44949021-44949021**	chrX	44949021	44949021	1	0	100	W1194	25	KDM6A
**chrX:44949053-44949053**	chrX	44949053	44949053	1	0	100	V1205	25	KDM6A
**chrX:44949073-44949073**	chrX	44949073	44949073	1	0	100	Q1212	25	KDM6A
**chrX:44949107-44949107**	chrX	44949107	44949107	1	0	100	G1223	25	KDM6A
**chrX:44949124-44949124**	chrX	44949124	44949124	1	0	100	Q1229	25	KDM6A
**chrX:44949139-44949141**	chrX	44949139	44949141	3	0	100	C1234	25	KDM6A
**chrX:44949994-44949994**	chrX	44949994	44949994	1	0	100	R1255	26	KDM6A
**chrX:44949995-44949995**	chrX	44949995	44949995	1	0	100	R1255	26	KDM6A
**chrX:44949999-44949999**	chrX	44949999	44949999	1	0	100	Y1256	26	KDM6A
**chrX:44950066-44950066**	chrX	44950066	44950066	1	0	100	R1279	26	KDM6A
**chrX:44950067-44950067**	chrX	44950067	44950067	1	0	100	R1279	26	KDM6A
**chrX:44966680-44966680**	chrX	44966680	44966680	1	0	100	Q1302	27	KDM6A
**chrX:44966716-44966716**	chrX	44966716	44966716	1	0	100	G1314	27	KDM6A
**chrX:44969370-44969370**	chrX	44969370	44969370	1	0	100	G1321	27	KDM6A
**chrX:44969396-44969396**	chrX	44969396	44969396	1	0	100	R1351	28	KDM6A
**chrX:44969447-44969447**	chrX	44969447	44969447	1	0	100	G1367	28	KDM6A
**chrX:44969479-44969480**	chrX	44969479	44969480	2	0	100	Y1387	28	KDM6A
**chrX:53409197-53409199**	chrX	53409197	53409199	3	0	100	G1131	22	SMC1A
**chrX:53423405-53423405**	chrX	53423405	53423405	1	0	100	G899	17	SMC1A
**chrX:53423416-53423416**	chrX	53423416	53423416	1	0	100	R895	17	SMC1A
**chrX:53423473-53423473**	chrX	53423473	53423473	1	0	100	K854	17	SMC1A
**chrX:53423538-53423538**	chrX	53423538	53423538	1	0	100	Non-coding		SMC1A
**chrX:53426514-53426514**	chrX	53426514	53426514	1	0	100	K853	16	SMC1A

**chrX:53426586-53426586**	chrX	53426586	53426586	1	0	100	K829	16	SMC1A
**chrX:53426617-53426617**	chrX	53426617	53426617	1	0	100	I819	16	SMC1A
**chrX:53432007-53432009**	chrX	53432007	53432009	3	0	100	R711	14	SMC1A
**chrX:53432579-53432579**	chrX	53432579	53432579	1	0	100	R586	11	SMC1A
**chrX:53432580-53432580**	chrX	53432580	53432580	1	0	100	R586	11	SMC1A
**chrX:53441940-53441942**	chrX	53441940	53441942	3	0	100	R96	2	SMC1A
**chrX:123164892-12316489**	chrX	123164892	123164892	1	0	100	R69	5	STAG2
**chrX:123164893-12316489**	chrX	123164893	123164893	1	0	100	R69	5	STAG2
**chrX:123164963-12316496**	chrX	123164963	123164963	1	0	100	K92	5	STAG2
**chrX:123171394-12317139**	chrX	123171394	123171394	1	0	100	W102	7	STAG2
**chrX:123176440-12317644**	chrX	123176440	123176447	8	0	100	F136	7	STAG2
**chrX:123176451-12317645**	chrX	123176451	123176451	1	0	100	Q140	7	STAG2
**chrX:123176470-12317647**	chrX	123176470	123176470	1	0	100	R146	7	STAG2
**chrX:123176490-12317649**	chrX	123176490	123176490	1	0	100	D153	7	STAG2
**chrX:123176495-12317649**	chrX	123176495	123176495	1	0	100	E154	7	STAG2
**chrX:123179017-12317901**	chrX	123179017	123179017	1	0	100	S156	8	STAG2
**chrX:123179050-12317905**	chrX	123179050	123179050	1	0	100	Q167	8	STAG2
**chrX:123179092-12317909**	chrX	123179092	123179092	1	0	100	V181	8	STAG2
**chrX:123179101-12317910**	chrX	123179101	123179101	1	0	100	R184	8	STAG2
**chrX:123179212-12317921**	chrX	123179212	123179212	1	0	100	L221	8	STAG2
**chrX:123179216-12317921**	chrX	123179216	123179216	0	1	0	A222	8	STAG2
**chrX:123181208-12318120**	chrX	123181208	123181208	1	0	100	M224	9	STAG2
**chrX:123181218-12318121**	chrX	123181218	123181218	1	0	100	T228	9	STAG2
**chrX:123181240-12318124**	chrX	123181240	123181240	1	0	100	L235	9	STAG2
**chrX:123181245-12318124**	chrX	123181245	123181245	1	0	100	L237	9	STAG2
**chrX:123181255-12318125**	chrX	123181255	123181255	1	0	100	N240	9	STAG2
**chrX:123181287-12318128**	chrX	123181287	123181287	1	0	100	E251	9	STAG2
**chrX:123181346-12318134**	chrX	123181346	123181346	1	0	100	K270	9	STAG2
**chrX:123181348-12318134**	chrX	123181348	123181348	1	0	100	R271	9	STAG2
**chrX:123181394-12318139**	chrX	123181394	123181394	1	0	100	E273	9	STAG2
**chrX:123197784-12319778**	chrX	123197784	123197784	1	0	100	Y636	20	STAG2
**chrX:123197812-12319781**	chrX	123197812	123197812	1	0	100	I646	20	STAG2
**chrX:123197834-12319783**	chrX	123197834	123197834	1	0	100	S653	20	STAG2
**chrX:123197842-12319784**	chrX	123197842	123197842	1	0	100	Q656	20	STAG2
**chrX:123197864-12319786**	chrX	123197864	123197864	1	0	100	D663	20	STAG2
**chrX:123197867-12319786**	chrX	123197867	123197867	1	0	100	K664	20	STAG2
**chrX:123197901-12319790**	chrX	123197901	123197901	1	0	100	E675	20	STAG2
**chrX:123205041-12320504**	chrX	123205041	123205041	1	0	100	Q801	25	STAG2
**chrX:123205051-12320505**	chrX	123205051	123205051	1	0	100	S804	25	STAG2
**chrX:123205083-12320508**	chrX	123205083	123205083	1	0	100	Y815	25	STAG2
**chrX:123205173-12320517**	chrX	123205173	123205173	1	0	100	D845	25	STAG2
**chrX:123217296-12321729**	chrX	123217296	123217296	1	0	100	E984	29	STAG2
**chrX:123217337-12321733**	chrX	123217337	123217337	1	0	100	L997	29	STAG2
**chrX:123217380-12321738**	chrX	123217380	123217380	1	0	100	R1012	29	STAG2
**chrX:123220440-12322044**	chrX	123220440	123220440	1	0	100	R1033	30	STAG2
**chrX:123220476-12322047**	chrX	123220476	123220476	1	0	100	R1045	30	STAG2
**chrX:123220477-12322047**	chrX	123220477	123220477	1	0	100	R1045	30	STAG2

**chrX:123220516-12322051**	chrX	123220516	123220516	1	0	100	S1058	30	STAG2
**chrX:123220578-12322057**	chrX	123220578	123220578	1	0	100	T1079	30	STAG2
**chrX:129148873-12914887**	chrX	129148873	129148875	3	0	100	L709	3	BCORL1
**chrX:129149098-12914910**	chrX	129149098	129149100	3	0	100	R784	3	BCORL1
**chrX:129155104-12915510**	chrX	129155104	129155106	3	0	100	R1196	4	BCORL1
**chrX:129159276-12915927**	chrX	129159276	129159278	3	0	100	R1334	6	BCORL1
**chrX:133511649-13351164**	chrX	133511649	133511649	1	0	100	M1	2	PHF6
**chrX:133511652-13351165**	chrX	133511652	133511652	1	0	100	S2	2	PHF6
**chrX:133511717-13351171**	chrX	133511717	133511717	1	0	100	R24	2	PHF6
**chrX:133511782-13351178**	chrX	133511782	133511782	1	0	100	C45	2	PHF6
**chrX:133527603-13352760**	chrX	133527603	133527603	1	0	100	Y105	4	PHF6
**chrX:133527636-13352763**	chrX	133527636	133527636	1	0	100	R116	4	PHF6
**chrX:133527637-13352763**	chrX	133527637	133527637	1	0	100	R116	4	PHF6
**chrX:133527960-13352796**	chrX	133527960	133527960	1	0	100	K132	5	PHF6
**chrX:133527979-13352797**	chrX	133527979	133527979	1	0	100	E139	5	PHF6
**chrX:133527982-13352798**	chrX	133527982	133527982	1	0	100	A140	5	PHF6
**chrX:133547589-13354758**	chrX	133547589	133547589	1	0	100	R164	6	PHF6
**chrX:133547614-13354761**	chrX	133547614	133547614	1	0	100	N171	6	PHF6
**chrX:133547852-13354785**	chrX	133547852	133547852	1	0	100	Non-coding		PHF6
**chrX:133547940-13354794**	chrX	133547940	133547940	1	0	100	R225	7	PHF6
**chrX:133547941-13354794**	chrX	133547941	133547941	1	0	100	R225	7	PHF6
**chrX:133547983-13354798**	chrX	133547983	133547983	1	0	100	H239	7	PHF6
**chrX:133547992-13354799**	chrX	133547992	133547992	1	0	100	C242	7	PHF6
**chrX:133549101-13354910**	chrX	133549101	133549101	1	0	100	D262	8	PHF6
**chrX:133549136-13354913**	chrX	133549136	133549136	1	0	100	R274	8	PHF6
**chrX:133549139-13354913**	chrX	133549139	133549139	1	0	100	G275	8	PHF6
**chrX:133549151-13354915**	chrX	133549151	133549151	1	0	100	Non-coding	9	PHF6
**chrX:133551224-13355122**	chrX	133551224	133551224	1	0	100	G287	9	PHF6
**chrX:133551254-13355125**	chrX	133551254	133551254	1	0	100	C297	9	PHF6
**chrX:133551305-13355130**	chrX	133551305	133551305	1	0	100	I314	10	PHF6
**chrX:133559245-13355924**	chrX	133559245	133559245	1	0	100	N328	10	PHF6
**chrX:133559304-13355930**	chrX	133559304	133559304	1	0	100	G348	10	PHF6
**chrX:133559338-13355933**	chrX	133559338	133559338	1	0	100	Q359	10	PHF6
**chrX:133559350-13355935**	chrX	133559350	133559350	1	0	100	N363		PHF6

Supplemental Table 4-Mutations

Patient ID	Diagnosis	Additional information	scDNA seg	Function	Protein	Coding impact	ClinVar	DANN	Variant type	RefSeq transcript id	Gene	cDNA	dbSNP rsids	# Genotyped Cells	# Mutated Cells	AML-MD-Vehicle.cells
**AML-MD2**	AML	Kariotypically normal acute myeloid leukemia with monocytes differentiation and at least single lineage dysplasia (44% Blasts by morphology, 63% atypical mieloblast by flow cytometry).	FLT3:chr13:28608341:T/C	coding	FLT3:p.Y572C	missense	Likely Pathogenic	1	SNV	NM_004119.3	FLT3	c.1715A>G	rs121913491	2105 (93%)	98 (5%)	2.4% VAF by cell count 2.0% VAF by read count
			FLT3:chr13:28592642:C/A	coding	FLT3:p.D835Y	missense	Pathogenic	1	SNV	NM_004119.3	FLT3	c.2503G>T	rs121913488	2060 (91%)	158 (8%)	4.0% VAF by cell count 3.3% VAF by read count
			EZH2:chr7:148543648:C/A	NMD, coding	EZH2:p.E54*	nonsense		1	SNV	NM_004456.5	EZH2	c.160G>T		1879 (80%)	943 (50%)	25.8% VAF by cell count 22.6% VAF by read count
			EZH2:chr7:148506477:C/T	coding, splicing	EZH2:p.V679M	missense		1	SNV	NM_004456.5	EZH2	c.2035G>A	rs1321951994	2060 (91%)	2038 (99%)	70.2% VAF by cell count 78.4% VAF by read count
			NRAS:chr1:115258745:C/G	coding	NRAS:p.G13R	missense	Likely Pathogenic, Pathogenic	1	SNV	NM_002524.5	NRAS	c.37G>C	rs121434595	1957 (86%)	290 (15%)	7.5% VAF by cell count 6.0% VAF by read count
			RUNX1:chr21:36252884:C/A	coding	RUNX1:p.D160Y	missense		0.99	SNV	NM_001754.5	RUNX1	c.478G>T		1964 (87%)	1255 (64%)	43.7% VAF by cell count 44.5% VAF by read count
			TET2:chr4:106196829:T/G	coding	TET2:p.L1721W	missense	Not Provided	0.93	SNV	NM_001127208.3	TET2	c.5162T>G	rs34402524	2056 (91%)	5803 (96%)	50.0% VAF by cell count 50.4% VAF by read count
			BCORL1:chrX:129159274:G/GA	NMD, coding	BCORL1:p.R1334Tfs*32	frameshift			Insertion	NM_021946.5	BCORL1	c.3999dup		1980 (87%)	713 (36%)	18.3% VAF by cell count 17.5% VAF by read count
**V18**	AML	Kariotypically normal	NPM1:chr5:170837543:C/CTCTG	coding	NPM1:p.W288Cfs*12	frameshift	Pathogenic		Insertion	NM_002520.7	NPM1	c.860_863dup	rs587776806	2054 (70%)	1750 (85%)	47.9% VAF by cell count 51.6% VAF by read count
			FLT3:chr13:28608243:T/TGAGATCATATTCATATTC	coding	FLT3:p.E604_F605insEYEYDLKWE	In frame			Insertion	NM_004119.3	FLT3			2869 (98%)	2831 (99%)	96.0% VAF by cell count 95.5% VAF by read count
			IDH2:chr15:90631934:C/T	coding	IDH2:p.R140Q	missense	Likely Pathogenic, Pathogenic	1	SNV	NM_002168.4	IDH2	c.419G>A	rs121913502	1521 (52%)	1387 (91%)	49.3% VAF by cell count 50.6% VAF by read count
**V20**	AML	Kariotypically normal	DNMT3A:chr2:25457243:G/T	coding	DNMT3A:p.R882S	missense	Likely Pathogenic, Pathogenic	1	SNV	NM_022552.5	DNMT3A	c.2644C>A	rs377577594	215 (96%)	208 (97%)	51.6% VAF by cell count 65.6% VAF by read count
			TET2:chr4:106156782:AC/A	NMD, coding	TET2:p.P562Qfs*6	frameshift			Deletion	NM_001127208.3	TET2	c.1685del		163 (73%)	45 (28%)	13.8% VAF by cell count 10.8% VAF by read count
			NPM1:chr5:170837545:C/CTGCA	coding	NPM1:p.W288Cfs*12	frameshift	Pathogenic		Insertion	NM_002520.7	NPM1	c.863_864insCATG	rs1554138188	203 (91%)	181 (89%)	48.3% VAF by cell count 48.9% VAF by read count
			FLT3:chr13:28608231:A/AGAAGTA	coding	FLT3:p.E608_N609insEV	in frame			Insertion	NM_004119.3	FLT3			224 (100%)	101 (45%)	22.8% VAF by cell count 23.1% VAF by read count
**P349**	MPN	Kariotypically normal, triple-negative	KMT2A:chr11:118373725:T/A	coding	KMT2A:p.L2373H	missense		0.93	SNV	NM_001197104.2	KMT2A	c.7118T>A		4912 (80%)	4894 (100%)	49.9% VAF by cell count 42.4% VAF by read count
			FLT3:chr13:28592644:C/A	coding	FLT3:p.R834L	missense		1	SNV	NM_004119.3	FLT3	c.2501G>T		1903 (38%)	250 (13%)	6.6% VAF by cell count 5.6% VAF by read count
			SETBP1:chr18:42532604:A/G	coding	SETBP1:p.H1100R	missense	Benign, Likely Benign	0.99	SNV	NM_015559.3	SETBP1	c.3299A>G	rs149162154	4358 (86%)	1434 (33%)	16.7% VAF by cell count 14.8% VAF by read count
**P350**	MPN	Kariotypically normal, triple-negative	NRAS:chr1:115258747:C/T	coding	NRAS:p.G12D	missense	Likely Pathogenic, Pathogenic	1	SNV	NM_002524.5	NRAS	c.35G>A	rs121913237	3169 (81%)	2486 (78%)	41.1% VAF by cell count 40.4% VAF by read count
			DNMT3A:chr2:25470484:C/A	coding	DNMT3A:p.W330C	missense		0.99	SNV	NM_022552.5	DNMT3A	c.990G>T		585 (24%)	164 (28%)	14.0% VAF by cell count 7.4% VAF by read count
			IDH1:chr2:209113113:G/A	coding	IDH1:p.R132C	missense	Likely Pathogenic, Pathogenic	1	SNV	NM_005896.4	IDH1	c.394C>T	rs121913499	2317 (94%)	241 (10%)	5.5% VAF by cell count 5.3% VAF by read count
			TET2:chr4:106156187:C/T	coding	TET2:p.P363L	missense	Not Provided	1	SNV	NM_001127208.3	TET2	c.1088C>T	rs17253672	2125 (86%)	1994 (94%)	49.9% VAF by cell count 51.8% VAF by read count

Human primary AML-MD2 sample was obtained from the Division of Hematology Biorepository at University of Utah. AML V18 and V20 samples were from the Department of Internal Medicine I, Division of Hematology & Hemostaseology, Medical University of Vienna, Austria. Triple-negative MPN samples P349 and P350 were provided by the Department of Hematology, Transplantation and Internal Medicine, University Clinical Centre, Medical University of Warsaw and Department of Hematology, Institute of Hematology and Blood Transfusion, Warsaw, Poland. Additional information about the samples including somatic mutations detected by sctDNA-seq are included in Supplemental Table S4.
